# Aerobic copper-promoted oxidative dehydrosulfurative carbon–oxygen cross-coupling of 3,4-dihydropyrimidine-1*H*-2-thiones with alcohols†

**DOI:** 10.1039/d1ra07713a

**Published:** 2021-11-17

**Authors:** Jihong Lee, Yujeong Kwon, Dong-Chan Lee, Jeong-Hun Sohn

**Affiliations:** Department of Chemistry, Chungnam National University Daejeon 34134 Republic of Korea sohnjh@cnu.ac.kr; Department of Chemistry and Biochemistry, University of Nevada Las Vegas 4505 S. Maryland Parkway, Box 454003 Las Vegas Nevada 89154-4003 USA Dong-Chan.Lee@unlv.edu

## Abstract

An aerobic Cu-promoted oxidative dehydrosulfurative carbon–oxygen cross-coupling of 3,4-dihydropyrimidin-1*H*-2-thiones (DHPMs) with both aliphatic and aromatic alcohols is described. Together with the ready availability of DHPMs and both alcohols, the method furnishes facile access to biologically valuable 2-alkoxypyrimidines with rapid diversification.

## Abstract 2

A wide range of readily available DHPMs and alcohols makes the presented reaction an attractive method to access biologically valuable 2-alkoxypyrimidine derivatives with rapid diversification.

## Graphical abstract

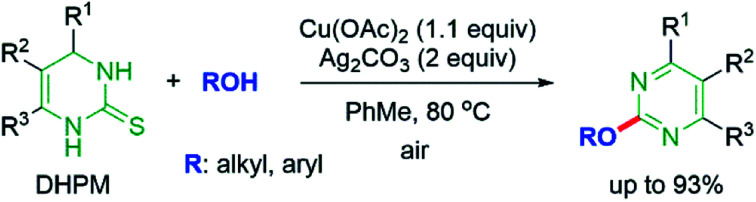

Pyrimidine motifs have received great interest from organic and medicinal chemists owing to their intriguing biological profile as well as being parts of DNA and RNA.^1^ Extensive research on this privileged scaffold has brought to the market dozens of drugs with highly potent activity against disease-causing microbes, viruses, and mycobacteria as well as cancer, inflammation, hypertension, and diabetes.^2^ In particular, the 2-alkoxypyrimidine structure has been incorporated into commercial herbicides such as bispyribac-sodium^3^ and pyriminobac-methyl,^4^ and osteogenesis inducers including purmorphamine (Fig. 1).^5^ In addition, 2-alkoxypyrimidine compounds are known to be selective PDGFRα inhibitors which induce apoptosis and autophagy in carcinoma cells^6^ and potent NAD^+^-dependent DNA ligase (LigA) inhibitors as potential antibacterial agents.^7^

**Fig. 1:**
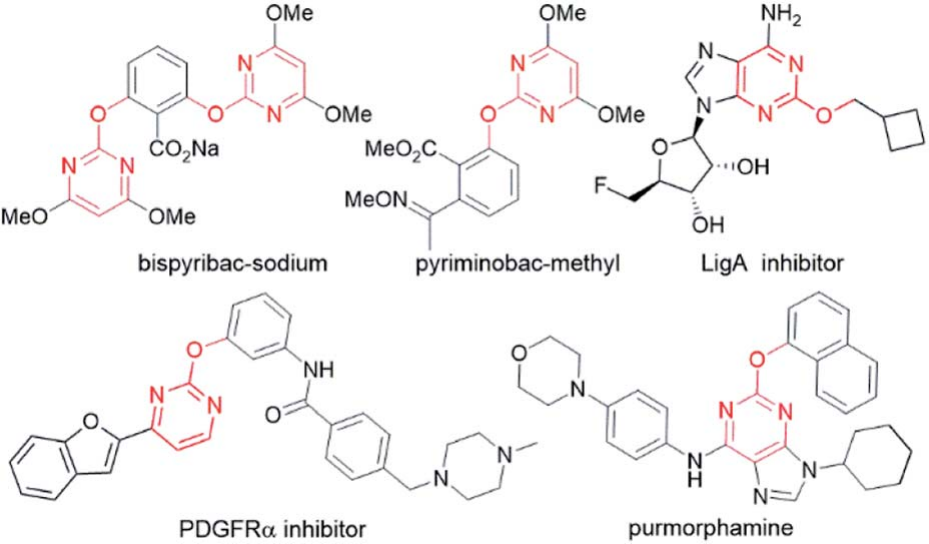
Biologically valuable 2-alkoxypyrimidine derivatives.

For the preparation of 2-alkoxypyrimidines, nucleophilic aromatic substitution^8^ or Pd-catalyzed C–O cross-coupling of 2-(pseudo)halopyrimidine compounds with alcohols has been commonly performed (Scheme 1A).^9^ In most cases, these strategies require tedious multistep synthesis of the densely substituted pyrimidine partners and, thus have limitation in preparing the diverse pyrimidine derivatives rapidly. In addition, competing β-hydride elimination reactions are an additional huddle in metal-catalyzed C–O coupling of (pseudo)halides with aliphatic 1° or 2° alcohols.

**Scheme 1:**
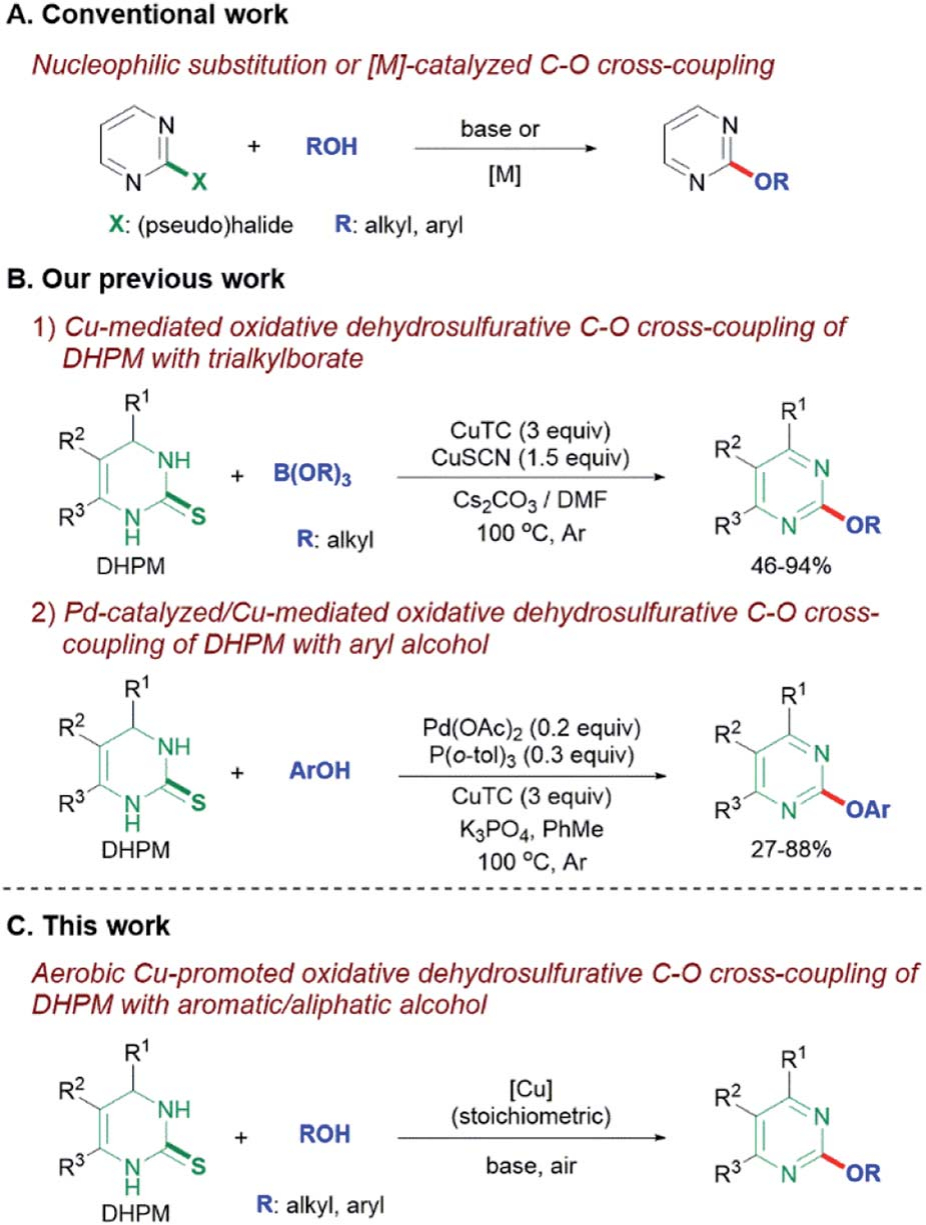
Synthetic strategy to 2-alkoxypyrimidine derivatives. (A) Conventional synthesis, (B) our previous synthesis, (C) this work.

For more efficient syntheses of pyrimidine derivatives with rapid diversification, we have employed 3,4-dihydropyrimidin-1*H*-2-thione (DHPM), which can be readily prepared by the well-known Biginelli three-component reaction,^10^ as an alternative to the 2-(pseudo)halopyrimidine. We recently developed the Cu-mediated oxidative dehydrosulfurative C–O cross-coupling of DHPMs with trialkylborates to generate the corresponding 2-alkyloxypyrimidines (Scheme 1B1).^11^ The reaction method with triarylborates was also found to be applicable to the synthesis of 2-aryloxypyrimidines, but not practical due to non-commercial availability and non-trivial preparation of most of the triarylborates. We subsequently developed Libeskind–Srogle-type^12^ Pd-catalyzed/Cu-mediated oxidative dehydrosulfurative C–O cross-coupling of DHPMs with readily available aryl alcohols for the synthesis of 2-aryloxypyrimidines (Scheme 1B2).^13^ Although both reaction methods offered the desired 2-alkoxypyrimidine products in moderate to good yields, their reaction conditions require large amounts of metals – 4.5 equivalents of Cu for borates and the mixture of 0.2 equivalents of Pd and 3 equivalents of Cu for aryl alcohols. The large amount of metals required for these reactions led us to pursue an alternative method that reduce the amount of Cu source and can be used for both aromatic and aliphatic alcohols. We report herein a dehydrosulfurative C–O cross-coupling of DHPMs with both aliphatic and aromatic alcohols with concomitant oxidative dehydrogenation (aromatization), promoted by stoichiometric amount of Cu under air (Scheme 1C). Considering the ready availability of DHPMs and both alcohols, the method furnishes a highly efficient access to various 2-aryl(alkyl)oxypyrimidine derivatives.

We commenced the studies with the reaction of DHPM 1a and PhOH as coupling partners in the presence of Cu(i)-thiophene-2-carboxylate (CuTC, 2 equiv.) and Cs_2_CO_3_ (2 equiv.) in toluene at 100 °C for 18 h under Ar atmosphere. The reaction provided the desired 2-phenoxypyrimidine 3a in 23% yield (entry 1, Table 1), which led us to investigate the reaction by varying reaction parameters. When copper(i) 3-methylsalicylate (CuMeSal) was used instead of CuTC, the desired product was produced in 32% yield (entry 2). The reaction yield was increased to 69% when Cu(OAc)_2_ was used (entry 3) while other Cu(i) or Cu(ii) sources, such as CuCl, CuI, CuBr, or CuBr_2_, were not effective (entries 4–7). With respect to base, Ag_2_CO_3_, which gave the desired product in 91% yield, was superior to other bases examined in the studies, such as Cs_2_CO_3_, K_2_CO_3_, *t*-BuOK, CsF, and Na_2_CO_3_ (entries 8–12). Toluene was better than other solvents, such as *o*-xylene, 1,4-dioxane, *N*,*N*-dimethylformamide (DMF), *N*-methyl-2-pyrrolidone (NMP), or dimethylsulfoxide (DMSO) for the reaction (entries 13–17). With respect to the amount of Cu(OAc)_2_, it was shown that 1.5 equivalents of Cu(OAc)_2_ was as effective as 2 equivalents of Cu(OAc)_2_ in the production of the desired product (entry 18). We found that the reaction under air proceeded as efficiently as that under Ar atmosphere (entry 19), which means that oxygen is not likely to participate in the reaction process. There was no difference in the reaction yield between the reaction temperature at 80 °C and 100 °C (entry 20). Further investigation of the reaction exhibited that 1–1.5 equivalents of Cu(OAc)_2_ with no other additives have almost the same effect on the production of the desired product under air (entries 19–22). Less than 1 equivalent of Cu(OAc)_2_ provided lower yields of the desired product (entries 23 and 24).

**Table 1:** Optimization studies^a^

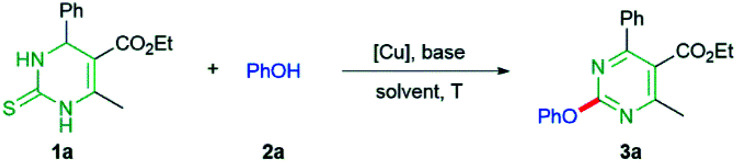					
Entry	Cu (equiv.)	Base	Solvent	*T* (°C)	Yield (%)
1	CuTC (2)	Cs_2_CO_3_	PhMe	100/Ar	23
2	CuMeSal (2)	Cs_2_CO_3_	PhMe	100/Ar	32
3	Cu(OAc)_2_ (2)	Cs_2_CO_3_	PhMe	100/Ar	69
4	CuCl (2)	Cs_2_CO_3_	PhMe	100/Ar	0
5	CuBr (2)	Cs_2_CO_3_	PhMe	100/Ar	0
6	CuI (2)	Cs_2_CO_3_	PhMe	100/Ar	0
7	CuBr_2_ (2)	Cs_2_CO_3_	PhMe	100/Ar	0
8	Cu(OAc)_2_ (2)	K_2_CO_3_	PhMe	100/Ar	75
9	Cu(OAc)_2_ (2)	*t*-BuOK	PhMe	100/Ar	54
10	Cu(OAc)_2_ (2)	CsF	PhMe	100/Ar	51
11	Cu(OAc)_2_ (2)	Ag_2_CO_3_	PhMe	100/Ar	91
12	Cu(OAc)_2_ (2)	Na_2_CO_3_	PhMe	100/Ar	63
13	Cu(OAc)_2_ (2)	Ag_2_CO_3_	*o*-Xylene	100/Ar	83
14	Cu(OAc)_2_ (2)	Ag_2_CO_3_	Dioxane	100/Ar	55
15	Cu(OAc)_2_ (2)	Ag_2_CO_3_	DMF	100/Ar	Trace
16	Cu(OAc)_2_ (2)	Ag_2_CO_3_	NMP	100/Ar	Trace
17	Cu(OAc)_2_ (2)	Ag_2_CO_3_	DMSO	100/Ar	Trace
18	Cu(OAc)_2_ (1.5)	Ag_2_CO_3_	PhMe	100/Ar	91
19	Cu(OAc)_2_ (1.5)	Ag_2_CO_3_	PhMe	100/air	90
20	Cu(OAc)_2_ (1.5)	Ag_2_CO_3_	PhMe	80/air	90
21	Cu(OAc)_2_ (1.2)	Ag_2_CO_3_	PhMe	80/air	89
22	Cu(OAc)_2_ (1)	Ag_2_CO_3_	PhMe	80/air	88
23	Cu(OAc)_2_ (0.5)	Ag_2_CO_3_	PhMe	80/air	74
24	Cu(OAc)_2_ (0.2)	Ag_2_CO_3_	PhMe	80/air	55

a Reaction conditions: DHPM 1a (0.18 mmol), PhOH 2a (0.20 mmol), base (0.36 mmol), and solvent (1.0 mL) for 18 h.

We assessed the scope of the reaction with various DHPM and aliphatic/aromatic alcohol coupling partners under optimal conditions using 1.1 equivalents of Cu(OAc)_2_. With regard to aryl alcohols, various substituents at the *para* position of phenyl ring were investigated (Scheme 2). When *p*-cresol was reacted with DHPM 1a, the desired aryloxypyrimidine 3b was generated in 51% yield.^14^ In the case of the aryl alcohols possessing electron-withdrawing group at the *para* position, the reaction with 1a provided the desired products in higher yields, comparing to the case of *p*-cresol; halide group, Cl, Br, or I, and other electron-withdrawing groups such as CN or NO_2_ afforded the corresponding products 3c–g in 77–90% yields. In particular, formyl group, which provided 3h in low yield (27%) in the previous Pd-catalyzed/Cu-mediated reaction, was also suitable functional group to produce 3h in 90% yield. We next assessed the reaction scope regarding DHPM substrates possessing various substituents at C4–C6 positions. For R^1^ at C5 position, both alkyl and aryl groups were compatible with the reaction; methyl, i-propyl, *t*-butyl, and phenyl groups afforded the corresponding products 3i–l in similarly high yields. With respect to C6 substituents (R^2^), the reaction of DHPM containing no C6 substituent with phenol gave the desired product 3m in 88% yields. Ethyl and phenyl groups at the C6 position also provided the desired products 3n and 3o in good yields. For the C4 substituent (R^3^), both electron-donating and -withdrawing groups at the *para* position of C4 aryl were adequate for the reaction; tolyl, anisolyl, 4-fluorophenyl, 4-chlorophenyl, 4-bromophenyl, 4-nitrophenyl, and 3,5-dimethylphenyl groups provided the corresponding products 3p–v in 72–94% yields. Note that the presence of halide group in either alcohol or DHPM partner did not cause any potentially competitive C–O coupling on the halide-attached carbon. Instead of aryl group, styryl group at the C4 position yielding 3w in 82% yield was also proven to be compatible under the reaction conditions. The DHPM with heterocyclic thiophenyl or pyridinyl group, or bicyclic naphthyl group at the C4 position was efficiently transformed to the corresponding products 3x–z in 60–89% yields.

**Scheme 2:**
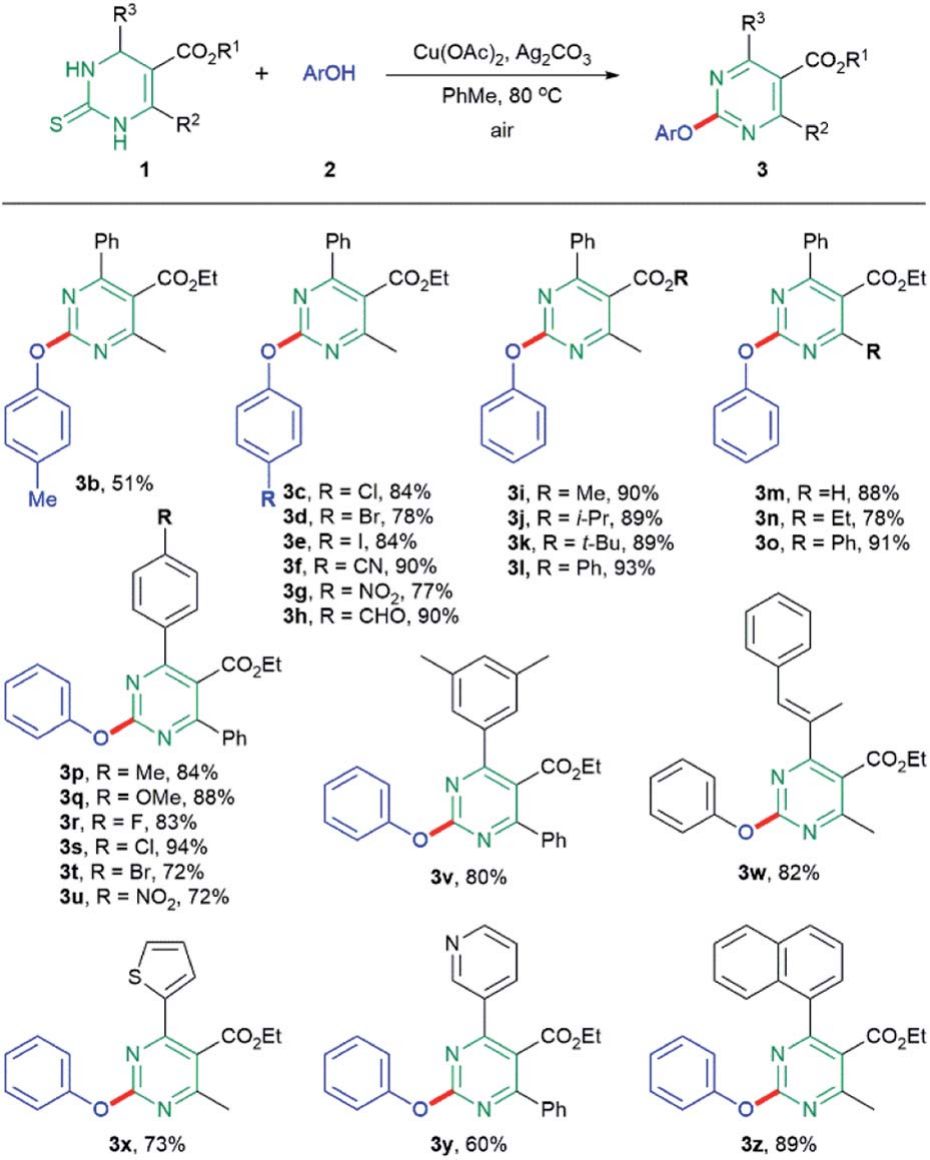
Scope of the reaction with respect to DHPMs and aromatic alcohols. Reaction conditions: DHPM 1 (0.18 mmol), aryl alcohol 2 (0.20 mmol), Cu(OAc)_2_ (0.20 mmol), Ag_2_CO_3_ (0.36 mmol) in PhMe (1.0 mL) at 80 °C under air.

We also assessed the reaction scope with respect to aliphatic alcohols (Scheme 3). When the reactions of DHPM 1a with 1° alcohols were performed, methoxy- (4a, 90%), ethoxy- (4b, 62%), *n*-propoxy- (4c, 60%), and *n*-butoxypyrimidine (4d, 79%) were obtained in moderate to good yields. Other aliphatic alcohols, such as benzyl- or phenethyl alcohol also provided the desired products 4e and 4f in 67% and 55% yields, respectively. The reaction with 2° alcohol i-PrOH produced the desired product 4g in 10% yield. This unacceptablly low yield increased to 54% in the presence of 1 equivalent of phenanthroline under the reaction conditions. The 3° alcohol, *t*-BuOH, did not appreciably provide the desired product. In these studies, we did not observe any possibly competitive β-hydride elimination side-reactions.

**Scheme 3:**
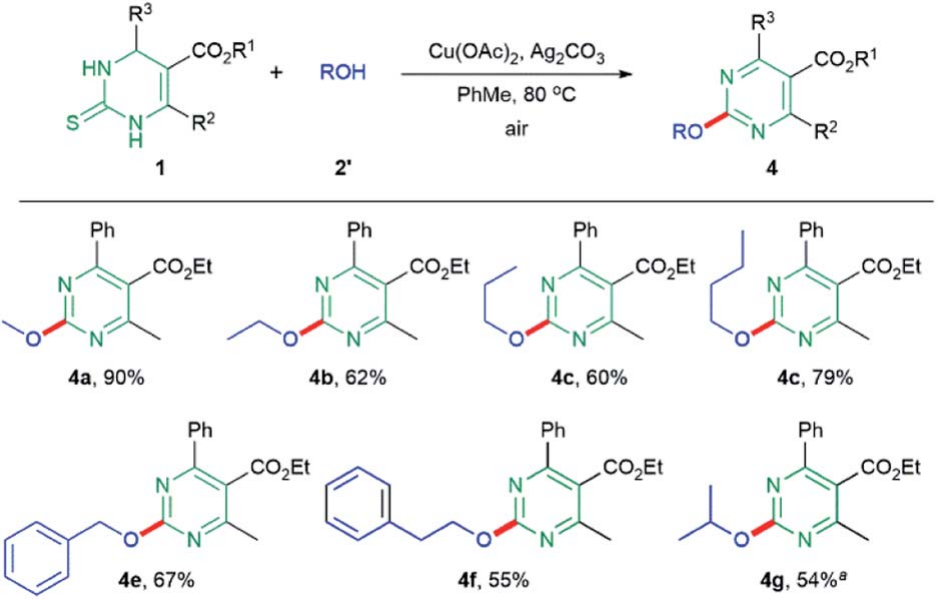
Scope of the reaction with respect to DHPMs and aliphatic alcohols. Reaction conditions: DHPM 1 (0.18 mmol), aliphatic alcohol 2′ (0.20 mmol), Cu(OAc)_2_ (0.20 mmol), Ag_2_CO_3_ (0.36 mmol) in PhMe (1.0 mL) at 80 °C under air.

In order to understand the mode of the reaction, we performed the reaction of DHPM 5 containing *t*-butyl group at the C4 position and obtained the debutylated product 6 in 75% yield (Scheme 4A). This result supports that the oxidative dehydrogenation (aromatization) proceeds *via* a radical intermediate as described in the literatures published by others^15^ and us.^11,13^ The reaction of 2-mercaptopyrimidine 7 with phenol provided the phenoxypyrimidine 8 (Scheme 4B), which can agree with the proposition that the C–S single bond generated after deprotonation participates in the coupling of DHPM. We found that the reaction of dihydropyrimidinyl thioether 9 with PhOH yielded pyrimidinyl thioether 10 as the major product along with a trace amount of 3a, which indicates that the oxidative dehydrogenation could proceed prior to the C–O coupling.

**Scheme 4:**
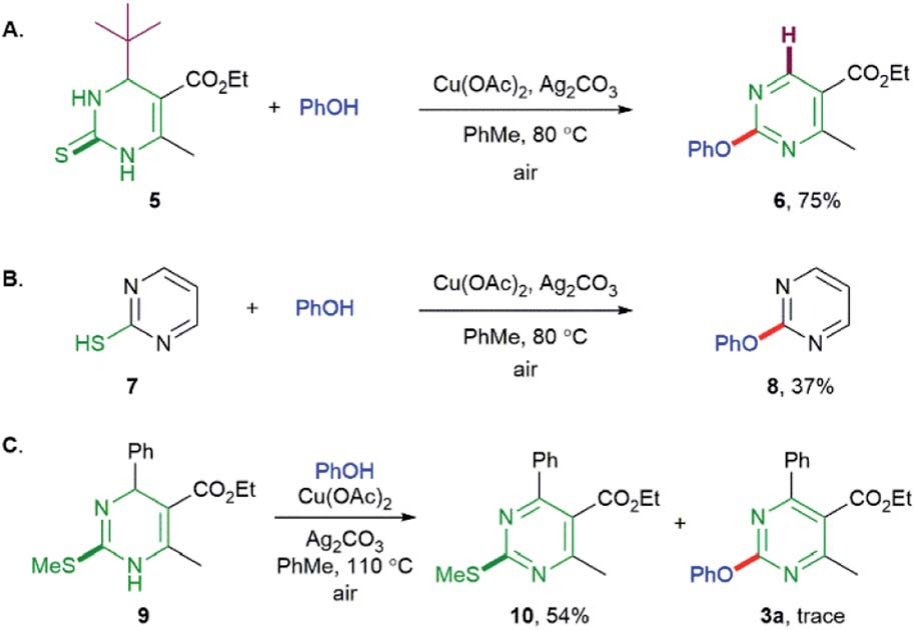
Control experiments. Reactions of phenol with (A) DHPM 5, (B) 2-mercaptopyrimidine 7, (C) dihydropyrimidinyl thioether 9.

Based on the results, we propose a plausible reaction mechanism as depicted in Scheme 5. Due to no significant difference in reaction yields between air and Ar atmosphere (Table 1), oxygen is unlikely to participate in the reaction process. Deprotonation and complexation with Ag_2_CO_3_ could generate 11 containing C–S single bond, which is then oxidized to pyrimidine 14 likely *via* radical intermediate 13 formed from nitrogen radical cation 12. The radical cation 12 could be produced from 11 by a single electron transfer.^16^ An oxidative addition of 14 to C u(i)OR generated from the reaction of Cu(i) species with alcohol could provide Cu(iii) complex 15, which is then converted to 2-alkoxypyrimidine by a reductive elimination.

**Scheme 5:**
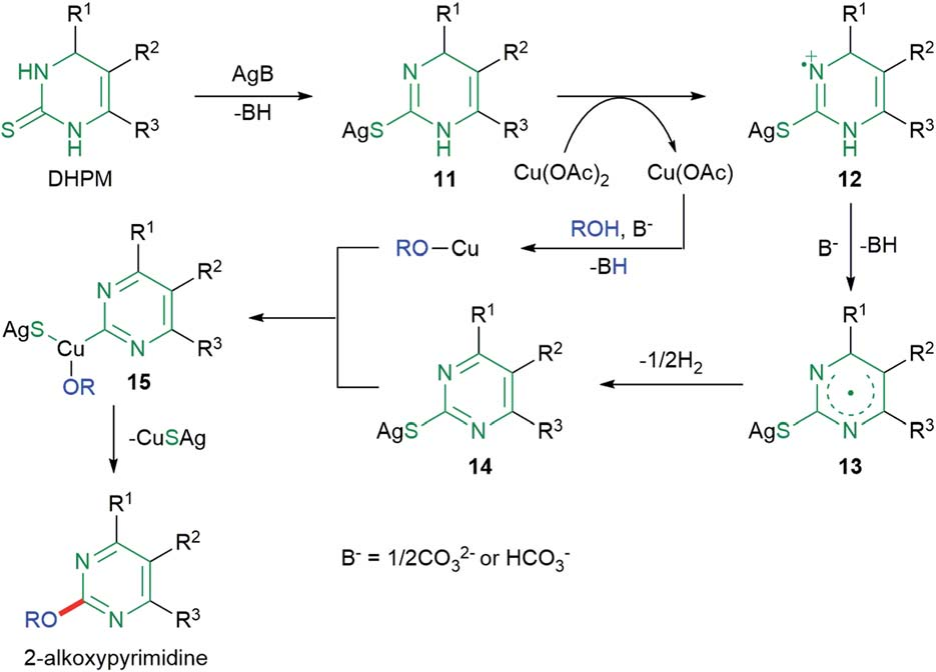
A plausible mechanism.

In summary, we have developed an aerobic Cu-promoted oxidative dehydrosulfurative carbon–oxygen cross-coupling of DHPMs. The reaction proceeded efficiently with diverse DHPMs and both aromatic and aliphatic alcohols. A wide range of readily available DHPMs and alcohols makes the presented reaction an attractive method to access to biologically and pharmacologically valuable 2-alkoxypyrimidine derivatives with rapid diversification.

## Conflicts of interest

There are no conflicts to declare.

## Supplementary Material

RA-011-D1RA07713A-s001
