# Supplementary material for: Aerobic copper-promoted oxidative dehydrosulfurative carbon–oxygen cross-coupling of 3,4-dihydropyrimidine-1H-2-thiones with alcohols
Source: RSC Adv. 2021 Nov 17;11(58):36821–5. doi: 10.1039/d1ra07713a (PMC9043584; doi:10.1039/d1ra07713a)

Electronic Supplementary Material (ESI) for RSC Advances.
This journal is © The Royal Society of Chemistry 2021

Supplementary Information

Aerobic copper-promoted oxidative dehydrosulfurative carbon-oxygen cross-coupling of 3,4-dihydropyrimidine-2-thiones with alcohols

Jihong Lee,[a] Yujeong Kwon,[a] Dong-Chan Lee,*[b] and Jeong-Hun Sohn*[a]

[a]Department of Chemistry, Chungnam National University, Daejeon 34134, Republic of Korea.

[b]Department of Chemistry and Biochemistry, University of Nevada, Las Vegas, Nevada 89154 United States.

E-mail: sohnjh@cnu.ac.kr, Dong-Chan.Lee@unlv.edu

Table of Contents

1. General information S2
2. Synthesis of DHPMs S2
3. General procedure for the synthesis of 2-alkoxypyrimidine S3
4. Characterization data for 2-alkoxypyrimidine Compounds S3
5. Reference S15
6. ^{1}H and ^{13}C NMR spectra of **1e, 3, 4, 5, 6, 8** and **10** S16
7. ^{19}F NMR spectrum of **3r** S54

1. General Information

Common solvents were purified before use. Toluene (PhMe) was purified by distillation from sodium-benzophenone ketyl. All reagents were reagent grade and purified where necessary. Reactions were monitored by thin layer chromatography (TLC) using Whatman pre-coated silica gel plates. Flash column chromatography was performed over ultra-pure silica gel (230-400 mesh) from Merck. Melting points (mp) were determined in opened capillary tubes and are uncorrected. ^{1}H NMR and ^{13}C NMR spectra were recorded on a Bruker AVANCE 600 (600 MHz) spectrometer using residual solvent peaks as an internal standard ($CHCl_3$: δ 7.26 ppm for proton and δ 77.16 ppm for carbon and DMSO-d_6: δ 2.50 ppm for proton and δ 39.52 ppm for carbon). Multiplicities for ^{1}H NMR are designated as: s = singlet, d = doublet, t = triplet, q = quartet, quint = quintet, sext =sextet, sept = septet, dd = doublet of doublets, dt = doublet of triplets, tt = triplet of triplets, m = multiplet and br = broad. Infrared spectra (IR) were recorded on JASCO FT/IR-4100 spectrometer and are reported in reciprocal centimeter (cm^{-1}). High resolution mass spectra (HRMS) were obtained on BrukermicroTOF-Q.

2. Synthesis of DHPMs

Following the literature procedure,[1] ethyl 6-methyl-4-phenyl-2-thioxo-1,2,3,4-tetrahydropyrimidine-5-carboxylate (**1a**),[1] methyl 6-methyl-4-phenyl-2-thioxo-1,2,3,4-tetrahydropyrimidine-5-carboxylate (**1b**),[1] isopropyl 6-methyl-4-phenyl-2-thioxo1,2,3,4-tetrahydropyrimidine-5-carboxylate (**1c**),[2] tert-butyl 6-methyl-4-phenyl-2-thioxo-1,2,3,4-tetrahydropyrimidine-5-carboxylate (**1d**),[3] phenyl 6-methyl-4-phenyl-2-thioxo-1,2,3,4-tetrahydropyrimidine-5-carboxylate (**1e**), ethyl 4-phenyl-2-thioxo-1,2,3,4-tetrahydropyrimidine-5-carboxylate (**1f**),[4] ethyl 6-ethyl-4-phenyl-2-thioxo-1,2,3,4-tetrahydropyrimidine-5-carboxylate (**1g**),[5] ethyl 4,6-diphenyl-2-thioxo-1,2,3,4-tetrahydropyrimidine-5-carboxylate (**1h**),[6] ethyl 6-phenyl-2-thioxo-4-(p-tolyl)-1,2,3,4-tetrahydropyrimidine-5-carboxylate (**1i**),[1] ethyl 4-(4-methoxyphenyl)-6-phenyl-2-thioxo-1,2,3,4-tetrahydropyrimidine-5-carboxylate (**1j**),[6] ethyl 4-(4-fluorophenyl)-6-phenyl-2-thioxo-1,2,3,4-tetrahydropyrimidine-5-carboxylate (**1k**),[7] ethyl 4-(4-chlorophenyl)-6-phenyl-2-thioxo-1,2,3,4-tetrahydropyrimidine-5-carboxylate (**1l**),[6] ethyl 4-(4-bromophenyl)-6-phenyl-2-thioxo-1,2,3,4-tetrahydropyrimidine-5-carboxylate (**1m**),[8] ethyl 4-(4-nitrophenyl)-6-phenyl-2-thioxo-1,2,3,4-tetrahydropyrimidine-5-carboxylate (**1n**),[6] ethyl 4-(3,5-dimethylphenyl)-6-phenyl-2-thioxo-1,2,3,4-tetrahydropyrimidine-5-carboxylate (**1o**),[9] ethyl (E)-6-phenyl-4-(2-phenylprop-1-en-1-yl)-2-thioxo-1,2,3,4-tetrahydropyrimidine-5-carboxylate (**1p**),[9] ethyl 6-phenyl-4-(thiophen-2-yl)-2-thioxo-1,2,3,4-tetrahydropyrimidine-5-carboxylate (**1q**),[10] ethyl 6-phenyl-4-(pyridin-3-yl)-2-thioxo-1,2,3,4-tetrahydropyrimidine-5-carboxylate (**1r**),[11] ethyl 4-(naphthalen-1-yl)-6-phenyl-2-thioxo-1,2,3,4-

tetrahydropyrimidine-5-carboxylate (**1s**),[12] ethyl 4-(tert-butyl)-6-methyl-2-thioxo-1,2,3,4-tetrahydropyrimidine-5-carboxylate (**5**)[13] were prepared.

Phenyl 6-methyl-4-phenyl-2-thioxo-1,2,3,4-tetrahydropyrimidine-5-carboxylate (**1e**).

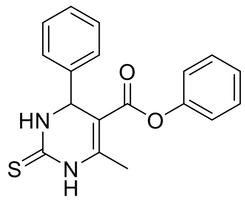

Recrystallized from a mixture of EtOH and H_2O (1:1). Yield: 1.78 g, 55% (10.0 mmol scale), white solid. ^{1}H NMR (600 MHz, DMSO-d_6): δ 10.55 (s, 1H), 9.82 (dd, J = 3.9, 1.9 Hz, 1H), 7.39 (m, 2H), 7.37 – 7.31 (m, 5H), 7.20 (m, 1H), 6.92 (m, 2H), 5.35 (d, J = 3.7 Hz, 1H), 2.37 (s, 3H). ^{13}C NMR (151 MHz, DMSO-d_6): δ 174.2, 163.7, 150.2, 147.5, 143.4, 129.3, 128.7, 127.8, 126.5, 125.6, 121.8, 99.4, 54.2, 17.5. IR (film) cm^{-1}: 3313, 3122, 2955, 1670, 1571, 1461, 1284, 1170, 1116, 762, 689; HRMS (ESI) m/z: $[M + H]^+$ calcd for $C_{18}H_{17}N_2O_2S$, 325.1011; found, 325.1035.

3. General procedure for the synthesis of 2-akoxypyrimidines and their characterization data.

To an oven-dried test tube with a magnetic stirring bar were added DHPM (0.18 mmol, 1.0 equiv), alcohol (0.20 mmol, 1.1 equiv), $Cu(OAc)_2$ (36 mg, 0.20 mmol, 1.1 equiv), Ag_2CO_3 (99 mg, 0.36 mmol, 2.0 equiv), in PhMe (1 mL). The reaction mixture was stirred at 80 °C in an oil bath under air until the DHPM completely disappeared. The mixture was filtered through a silica gel pad and washed with EtOAc (25 mL). The filtrate was washed with brine (7 mL), dried over $MgSO_4$, filtered, and concentrated under reduced pressure. The crude product was purified by flash column chromatography (silica gel; eluent: *n*-hexane/EtOAc, 10:1 to 4:1) to give the corresponding 2-alkoxypyrimidine.

4. Characterization data for 2-alkoxypyrimidine Compounds

Ethyl 4-methyl-2-phenoxy-6-phenylpyrimidine-5-carboxylate (**3a**)[14]

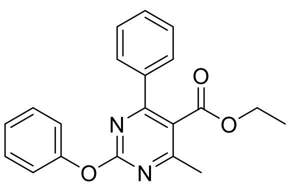

Eluent in chromatography: *n*-hexane/EtOAc 10:1. Yield: 53 mg, 88%; pale yellow oil. ^{1}H NMR (600 MHz, $CDCl_3$): δ 7.60 (m, 2H), 7.45 – 7.39 (m, 5H), 7.24 (m, 3H), 4.18 (q, J = 7.1 Hz, 2H), 2.57 (s, 3H), 1.06 (t, J = 7.1 Hz, 3H); ^{13}C NMR (151 MHz, $CDCl_3$): δ 169.3, 168.2, 166.8, 164.1, 153.0, 137.4, 130.4, 129.5, 128.6, 128.5, 125.3, 121.7, 121.2, 62.0, 22.9, 13.8.

Ethyl 4-methyl-6-phenyl-2-(p-tolyloxy)pyrimidine-5-carboxylate (**3b**).[14]

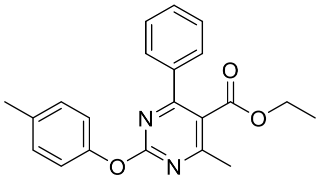

Eluent in chromatography: *n*-hexane/EtOAc 10:1. Yield: 31 mg, 51%; pale yellow oil ^{1}H NMR (600 MHz, $CDCl_3$): δ 7.59 (m, 2H), 7.46 – 7.37 (m, 3H), 7.20 (d, *J* = 8.1 Hz, 2H), 7.12 (m, 2H), 4.17 (q, *J* = 7.1 Hz, 2H), 2.57 (s, 3H), 2.37 (s, 3H), 1.06 (t, *J* = 7.1 Hz, 3H); ^{13}C NMR (151 MHz, $CDCl_3$): δ 169.2, 168.2, 166.9, 164.1, 150.7, 137.5, 134.9, 130.4, 130.0, 128.6, 128.5, 121.4, 121.1, 62.0, 22.8, 21.1, 13.8.

Ethyl 2-(4-chlorophenoxy)-4-methyl-6-phenylpyrimidine-5-carboxylate (**3c**).[14]

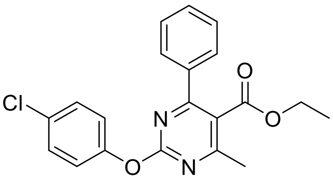

Eluent in chromatography: n-hexane/EtOAc 10:1. Yield: 56 mg, 78%; pale yellow solid. ^{1}H NMR (600 MHz, $CDCl_3$): δ 7.58 (m, 2H), 7.47 – 7.35 (m, 5H), 7.20 – 7.17 (m, 2H), 4.18 (q, *J* = 7.2 Hz, 2H), 2.57 (s, 3H), 1.06 (t, *J* = 7.1 Hz, 3H). ^{13}C NMR (151 MHz, $CDCl_3$): δ 169.4, 168.0, 166.8, 163.7, 151.4, 137.2, 130.62, 130.58, 129.6, 128.6, 128.5, 123.1, 121.5, 62.0, 22.8, 13.7.

Ethyl 2-(4-bromophenoxy)-4-methyl-6-phenylpyrimidine-5-carboxylate (**3d**).[15]

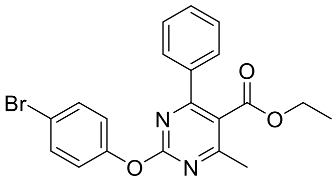

Eluent in chromatography: n-hexane/EtOAc 10:1. Yield: 59 mg, 78%; white solid. ^{1}H NMR (600 MHz, $CDCl_3$) δ 7.58 (m, 2H), 7.51 (m, 2H), 7.47 – 7.39 (m, 3H), 7.13 (m, 2H), 4.18 (q, *J* = 7.2 Hz, 2H), 2.56 (s, 3H), 1.05 (t, *J* = 7.2 Hz, 3H); ^{13}C NMR (151 MHz, $CDCl_3$): δ 169.4, 168.0, 166.8, 163.6, 151.9, 137.2, 132.5, 130.5, 128.6, 128.4, 123.5, 121.5, 118.3, 62.0, 22.8, 13.7.

*Ethyl 2-(4-iodophenoxy)-4-methyl-6-phenylpyrimidine-5-carboxylat*e (**3e**).[16]

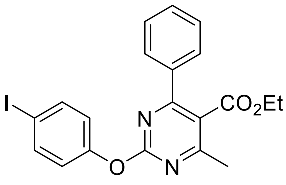

Eluent in chromatography: n-hexane/EtOAc 10:1. Yield: 70 mg, 84%; yellow solid. ^{1}H NMR (600 MHz, $CDCl_3$): δ 7.70 (m, 2H), 7.58 (m, 2H), 7.46 – 7.39 (m, 3H), 7.02 (m, 2H), 4.18 (q, J = 7.1 Hz, 2H), 2.57 (s, 3H), 1.06 (t, J = 7.2 Hz, 3H); ^{13}C NMR (151 MHz, $CDCl_3$): δ 169.4, 168.0, 166.8, 163.6, 152.8, 138.5, 137.2, 130.5, 128.6, 128.4, 123.9, 121.5, 89.1, 62.0, 22.9, 13.7.

Ethyl 2-(4-cyanophenoxy)-4-methyl-6-phenylpyrimidine-5-carboxylate (**3f**).[16]

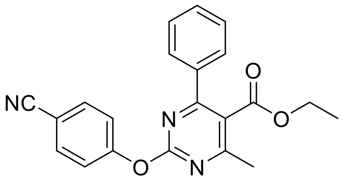

Eluent in chromatography: *n*-hexane/EtOAc 10:1. Yield: 59 mg, 90%; yellow oil. ^{1}H NMR (600 MHz, $CDCl_3$): δ 7.72 (m, 2H), 7.58 (m, 2H), 7.47 (m, 1H), 7.42 (m, 2H), 7.37 (m, 2H), 4.19 (q, J = 7.2 Hz, 2H), 2.58 (s, 3H), 1.07 (t, J = 7.2 Hz, 3H); ^{13}C NMR (151 MHz, $CDCl_3$): δ 169.7, 167.8, 166.9, 163.1, 156.3, 137.0, 133.9, 130.8, 128.7, 128.4, 122.6, 122.1, 118.6, 109.1, 62.2, 22.9, 13.8.

Ethyl 4-methyl-2-(4-nitrophenoxy)-6-phenylpyrimidine-5-carboxylate (**3g**).[17]

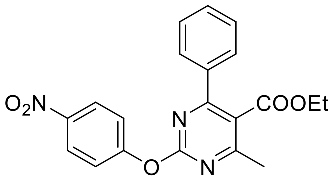

Eluent in chromatography: *n*-hexane/EtOAc 5:1. Yield: 48 mg, 70%; white solid.^{1}H NMR (600 MHz, $CDCl_3$): δ 8.30 (m, 2H), 7.58 (m, 2H), 7.47 (m, 1H), 7.42 (m, 4H), 4.20 (q, J = 7.1 Hz, 2H), 2.59 (s, 3H), 1.07 (t, J = 7.2 Hz, 3H); ^{13}C NMR (151 MHz, $CDCl_3$): δ 169.7, 167.8, 167.0, 163.0, 157.8, 145.0, 136.9, 130.8, 128.7, 128.4, 125.5, 122.2, 62.2, 22.9, 13.7.

Ethyl 2-(4-formylphenoxy)-4-methyl-6-phenylpyrimidine-5-carboxylate (**3h**).[16]

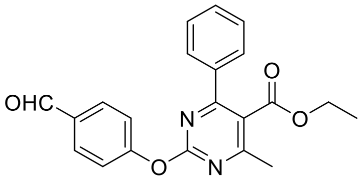

Eluent in chromatography: *n*-hexane/EtOAc 10:1. Yield: 59 mg, 90%; yellow oil. ^{1}H NMR (600 MHz, $CDCl_3$): δ 10.01 (s, 1H), 7.95 (m, 2H), 7.58 (m, 2H), 7.47 – 7.39 (m, 5H), 4.19 (q, J = 7.1 Hz, 2H), 2.58 (s, 3H), 1.07 (t, J = 7.2 Hz, 3H); ^{13}C NMR (151 MHz, $CDCl_3$): δ 191.1, 169.6, 167.9, 166.9, 163.3, 157.8, 137.1, 133.6, 131.5, 130.7, 128.7, 128.5, 122.2, 121.9, 62.1, 22.8, 13.7.

Methyl 4-methyl-2-phenoxy-6-phenylpyrimidine-5-carboxylate (**3i**).[18]

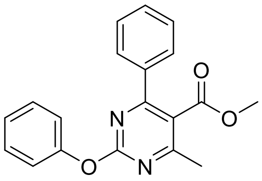

Eluent in chromatography: *n*-hexane/EtOAc 10:1. Yield: 51 mg, 90%; pale yellow oil. ^{1}H NMR (600 MHz, $CDCl_3$): δ 7.62 (d, *J* = 6.9 Hz, 2H), 7.51 – 7.41 (m, 5H), 7.33 – 7.24 (m, 3H), 3.73 (s, 3H), 2.59 (s, 3H); ^{13}C NMR (151 MHz, $CDCl_3$): δ 169.2, 168.6, 166.5, 164.0, 152.8, 137.2, 130.5, 129.4, 128.5, 128.3, 125.3, 121.6, 120.7, 52.6, 22.8.

Isopropyl 4-methyl-2-phenoxy-6-phenylpyrimidine-5-carboxylate (**3j**).[16]

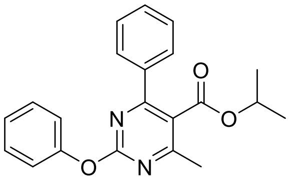

Eluent in chromatography: *n*-hexane/EtOAc 10:1. Yield: 55 mg, 89%; yellow oil. ^{1}H NMR (600 MHz, $CDCl_3$): δ 7.66 – 7.57 (m, 2H), 7.49 – 7.37 (m, 5H), 7.27 – 7.22 (m, 3H), 5.08 (sept, *J* = 6.2 Hz, 1H), 2.56 (s, 3H), 1.09 (d, *J* = 6.3 Hz, 6H); ^{13}C NMR (151 MHz, $CDCl_3$): δ 168.8, 167.5, 166.5, 163.8, 152.8, 137.3, 130.3, 129.4, 128.5, 128.4, 125.2, 121.6, 69.7, 22.6, 21.3.

Tert-butyl 4-methyl-2-phenoxy-6-phenylpyrimidine-5-carboxylate (**3k**).[16]

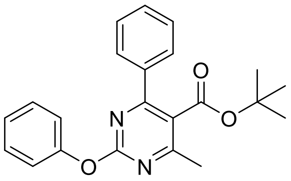

Eluent in chromatography: *n*-hexane/EtOAc 10:1. Yield: 58 mg, 89%; pale yellow oil. ^{1}H NMR (600 MHz, $CDCl_3$): δ 7.64 – 7.60 (m, 2H), 7.46 – 7.38 (m, 5H), 7.26 – 7.21 (m, 3H), 2.57 (s, 3H), 1.38 (s, 9H); ^{13}C NMR (151 MHz, $CDCl_3$): δ 168.7, 167.1, 166.3, 163.7, 153.0, 137.5, 130.3, 129.5, 128.7, 128.4, 125.2, 122.7, 121.7, 83.1, 27.8, 22.8.

Phenyl 4-methyl-2-phenoxy-6-phenylpyrimidine-5-carboxylate (**3l**).

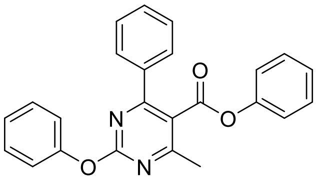

Eluent in chromatography: *n*-hexane/EtOAc 10:1. Yield: 64 mg, 93%; white solid, mp 97−99 °C. ^{1}H NMR (600 MHz, $CDCl_3$): δ 7.71 (m, 2H), 7.52 – 7.41 (m, 5H), 7.34 (m, 2H), 7.28 – 7.21 (m, 4H), 6.84 (m, 2H), 2.70 (s, 3H). ^{13}C NMR (151 MHz, $CDCl_3$): δ 169.7, 167.2, 166.7, 164.3, 152.9, 150.3, 137.4, 130.8, 129.63, 129.55, 128.9, 128.7, 126.5, 125.5, 121.7, 121.2, 120.5, 23.1; IR (film) cm^{-1}: 3021, 2963, 1717, 1566, 1491, 1421, 1356, 1263, 861, 731; HRMS (ESI) m/z: $[M + H]^+$ calcd for $C_{24}H_{19}N_2O_3$, 383.1396; found, 383.1383.

Ethyl 2-phenoxy-4-phenylpyrimidine-5-carboxylate (**3m**).

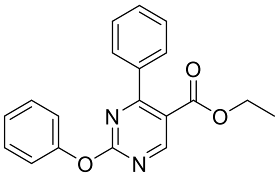

Eluent in chromatography: *n*-hexane/EtOAc 10:1. Yield: 57 mg, 88%; yellow viscous oil. ^{1}H NMR (600 MHz, $CDCl_3$): δ 8.94 (s, 1H), 7.59 (m, 2H), 7.50 – 7.41 (m, 5H), 7.28 (m, 1H), 7.24 (m, 2H), 4.22 (q, *J* = 7.1 Hz, 2H), 1.13 (t, *J* = 7.1 Hz, 3H); ^{13}C NMR (151 MHz, $CDCl_3$): δ 169.8, 166.0, 165.6, 162.2, 152.7, 137.2, 130.6, 129.8, 129.1, 128.3, 125.9, 121.7, 119.9, 61.8, 13.9; IR (film) cm^{-1}: 3045, 2942, 1716, 1569, 1534, 1489, 1417, 1313, 1237, 1124, 769, 697. HRMS (ESI) m/z: $[M + H]^+$ calcd for $C_{19}H_{17}N_2O_3$, 321.1239; found, 321.1252.

Ethyl 4-ethyl-2-phenoxy-6-phenylpyrimidine-5-carboxylate (**3n**).[16]

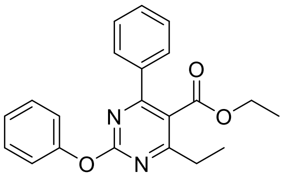

Eluent in chromatography: *n*-hexane/EtOAc 10:1. Yield: 49 mg, 78%; yellow oil. ^{1}H NMR (600 MHz, $CDCl_3$): δ 7.59 (m, 2H), 7.45 – 7.38 (m, 5H), 7.26 – 7.21 (m, 3H), 4.17 (q, *J* = 7.1 Hz, 2H), 2.84 (q, *J* = 7.5 Hz, 2H), 1.29 (t, *J* = 7.5 Hz, 3H), 1.06 (t, *J* = 7.1 Hz, 3H); ^{13}C NMR (151 MHz, $CDCl_3$): δ 173.6, 168.3, 166.7, 164.4, 153.0, 137.5, 130.4, 129.4, 128.6, 128.5, 125.3, 121.8, 120.9, 61.9, 29.1, 13.8, 12.8.

Ethyl 2-phenoxy-4,6-diphenylpyrimidine-5-carboxylate (**3o**).[16]

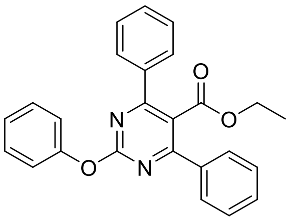

Eluent in chromatography: *n*-hexane/EtOAc 10:1. Yield: 65 mg, 91%; pale yellow oil. ^{1}H NMR (600 MHz, $CDCl_3$) δ 7.66 (m, 4H), 7.48 – 7.40 (m, 8H), 7.30 (m, 2H), 7.24 (tt, *J* = 7.3, 1.2 Hz, 1H), 4.07 (q, *J* = 7.1 Hz, 2H), 0.97 (t, *J* = 7.2 Hz, 3H); ^{13}C NMR (151 MHz, $CDCl_3$) δ 168.3, 167.6, 164.2, 153.0, 137.2, 130.4, 129.5, 128.56, 128.55, 125.3, 121.7, 121.0, 62.1, 13.6.

Ethyl 4-methyl-2-phenoxy-6-(p-tolyl)pyrimidine-5-carboxylate (**3p**).[14]

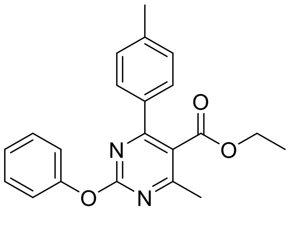

Eluent in chromatography: n-hexane/EtOAc 10:1. Yield: 53 mg, 84%; pale yellow oil. ^{1}H NMR (600 MHz, $CDCl_3$): δ 7.43 (d, *J* = 8.0 Hz, 2H), 7.33 (t, *J* = 7.9 Hz, 2H), 7.18 – 7.11 (m, 5H), 4.14 (q, *J* = 7.1 Hz, 2H), 2.48 (s, 3H), 2.30 (s, 3H), 1.05 (t, *J* = 7.1 Hz, 3H); ^{13}C NMR (151 MHz, $CDCl_3$): δ 169.0, 168.4, 166.5, 164.0, 153.0, 140.9, 134.5, 129.4, 129.3, 128.5, 125.3, 121.7, 121.0, 61.9, 22.8, 21.5, 13.8.

Ethyl 4-(4-methoxyphenyl)-2-phenoxy-6-phenylpyrimidine-5-carboxylate (**3q**).[16]

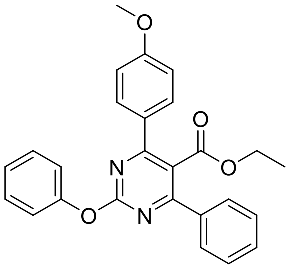

Eluent in chromatography: *n*-hexane/EtOAc10:1. Yield: 72 mg, 88%; brown oil. ^{1}H NMR (600 MHz, $CDCl_3$): δ 7.65 (m, 4H), 7.48 – 7.39 (m, 5H), 7.29 (m, 2H), 7.23 (t, *J* = 7.4 Hz, 1H), 6.93 (m, 2H), 4.10 (q, *J* = 7.1 Hz, 2H), 3.84 (s, 3H), 1.00 (t, *J* = 7.1 Hz, 3H); ^{13}C NMR (151 MHz, $CDCl_3$): δ 168.7, 167.6, 166.7, 164.2, 161.7, 153.1, 137.4, 130.4, 130.3, 129.5, 128.5, 128.5, 125.3, 121.7, 120.5, 114.1, 62.1, 55.5, 13.7.

Ethyl 4-(4-fluorophenyl)-2-phenoxy-6-phenylpyrimidine-5-carboxylate (**3r**).[16]

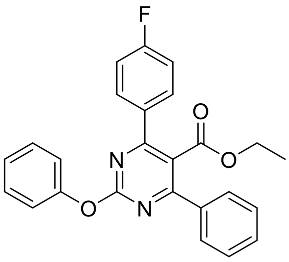

Eluent in chromatography: *n*-hexane/EtOAc 10:1. Yield: 62 mg, 83%; yellow oil. ^{1}H NMR (600 MHz, $CDCl_3$): δ 7.69 – 7.64 (m, 4H), 7.48 – 7.40 (m, 5H), 7.29 (d, J = 7.7 Hz, 2H), 7.24 (t, J = 7.4 Hz, 1H), 7.12 (m, 2H), 4.08 (q, J = 7.1 Hz, 2H), 0.99 (t, J = 7.1 Hz, 3H); ^{13}C NMR (151 MHz, $CDCl_3$): δ 168.3, 167.8, 166.4, 164.3 (d, $J_{C–F}$ = 251 Hz), 164.2, 152.9, 137.1, 133.3 (d, $J_{C–F}$ = 3.2 Hz), 130.8 (d, $J_{C–F}$ = 8.7 Hz), 130.5, 129.5, 128.6, 128.5, 125.4, 121.7, 120.8, 115.8 (d, $J_{C–F}$ = 22 Hz), 62.2, 13.6; ^{19}F NMR (565 MHz, $CDCl_3$): δ -109.85 (s).

Ethyl 4-(4-chlorophenyl)-2-phenoxy-6-phenylpyrimidine-5-carboxylate (**3s**).[16]

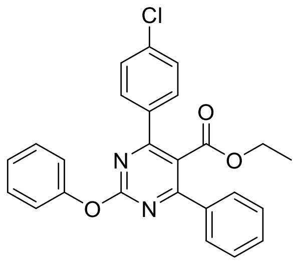

Eluent in chromatography: *n*-hexane/EtOAc 10:1. Yield: 73 mg, 94%; yellow oil. ^{1}H NMR (600 MHz, $CDCl_3$): δ 7.65 (m, 2H), 7.61 (m, 2H), 7.47 (m, 1H), 7.45 – 7.39 (m, 6H), 7.28 (m, 2H), 7.24 (m, 1H), 4.08 (q, J = 7.1 Hz, 2H), 0.99 (t, J = 7.1 Hz, 3H); ^{13}C NMR (151 MHz, $CDCl_3$): δ 168.1, 167.9, 166.3, 164.3, 152.9, 137.1, 136.9, 135.6, 130.6, 130.0, 129.6, 128.9, 128.6, 128.5, 125.5, 121.7, 120.9, 62.2, 13.6.

Ethyl 4-(4-bromophenyl)-2-phenoxy-6-phenylpyrimidine-5-carboxylate (**3t**).[16]

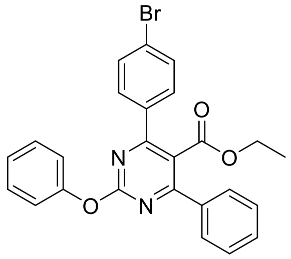

Eluent in chromatography: *n*-hexane/EtOAc 10:1. Yield: 62 mg, 72%; yellow oil. ^{1}H NMR (600 MHz, $CDCl_3$): δ 7.64 (m, 2H), 7.55 (dd, J = 20, 8.6 Hz, 4H), 7.49 – 7.40 (m, 5H), 7.28 (d, J = 7.7 Hz, 2H), 7.24 (t, J = 7.4 Hz, 1H), 4.08 (q, J = 7.1 Hz, 2H), 0.99 (t, J = 7.1 Hz, 3H); ^{13}C NMR (151 MHz, $CDCl_3$): δ 168.1, 168.0, 166.3, 164.3, 152.9, 137.1, 136.1, 131.9, 130.6, 130.2, 129.6, 128.63, 128.55, 125.5, 125.3, 121.7, 120.8, 62.3, 13.6.

Ethyl 4-(4-nitrophenyl)-2-phenoxy-6-phenylpyrimidine-5-carboxylate (**3u**).[16]

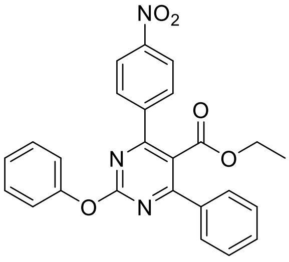

Eluent in chromatography: *n*-hexane/EtOAc 5:1. Yield: 72 mg, 90%; yellow solid. ^{1}H NMR (600 MHz, $CDCl_3$): δ 8.29 (m, 2H), 7.81 (m, 2H), 7.64 (m, 2H), 7.49 (m, 1H), 7.43 (m, 4H), 7.29 – 7.24 (m, 3H), 4.07 (q, J = 7.1 Hz, 2H), 0.97 (t, J = 7.1 Hz, 3H); ^{13}C NMR (151 MHz, $CDCl_3$): δ 168.4, 167.7, 165.3, 164.3, 152.8, 148.9, 143.1, 136.8, 130.8, 129.8, 129.6, 128.7, 128.5, 125.7, 123.7, 121.6, 121.1, 62.4, 13.6.

Ethyl 2-benzyl-4-(3,5-dimethylphenyl)-6-phenylpyrimidine-5-carboxylate (**3v**).[16]

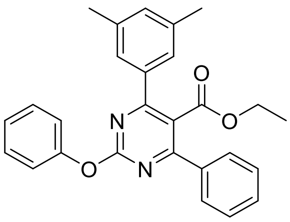

Eluent in chromatography: *n*-hexane/EtOAc 10:1. Yield: 61 mg, 80%; yellow oil. ^{1}H NMR (600 MHz, $CDCl_3$): δ 7.63 (m, 2H), 7.45 (m, 1H), 7.41 (t, J = 8.0 Hz, 4H), 7.28 (m, 4H), 7.23 (t, J = 7.4 Hz, 1H), 7.10 (s, 1H), 4.08 (q, J = 7.1 Hz, 2H), 2.34 (s, 6H), 1.01 (t, J = 7.1 Hz, 3H); ^{13}C NMR (151 MHz, $CDCl_3$): δ 168.4, 168.1, 167.3, 164.2, 153.1, 138.2, 137.3, 137.1, 132.1, 130.4, 129.5, 128.6, 128.5, 126.3, 125.3, 121.7, 121.1, 62.0, 21.4, 13.7.

Ethyl (E)-4-methyl-2-phenoxy-6-(1-phenylprop-1-en-2-yl)pyrimidine-5-carboxylate (**3w**).

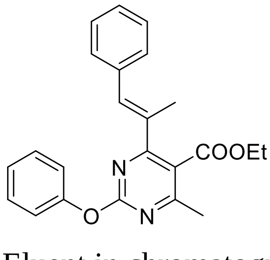

Eluent in chromatography: *n*-hexane/EtOAc 10:1. Yield: 55 mg, 82%; pale yellow oil. ^{1}H NMR (600 MHz, $CDCl_3$): δ 7.40 (t, J = 7.9 Hz, 2H), 7.36 (t, J = 7.5 Hz, 2H), 7.31 (d, J = 7.2 Hz, 2H), 7.28 (d, J = 7.3 Hz, 1H), 7.23 (m, 3H), 6.73 (s, 1H), 4.29 (q, J = 7.1 Hz, 2H), 2.54 (s, 3H), 2.22 (s, 3H), 1.27 (t, J = 7.1 Hz, 3H); ^{13}C NMR (151 MHz, $CDCl_3$): δ 170.7, 169.2, 168.2, 163.7, 153.0, 136.6, 136.0, 133.3, 129.5, 129.3, 128.5, 127.7, 125.3, 121.8, 120.9, 61.9, 22.9, 17.1, 14.3; IR (film) cm^{-1}: 3042, 2935,

1724, 1547, 1490, 1359, 1250, 1089, 697; HRMS (ESI) m/z: $[M + H]^+$ calcd for $C_{23}H_{23}N_2O_3$, 375.1709; found, 375.1737.

Ethyl 2-phenoxy-4-phenyl-6-(thiophen-2-yl)pyrimidine-5-carboxylate (**3x**).[16]

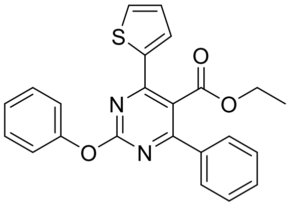

Eluent in chromatography: *n*-hexane/EtOAc 10:1. Yield: 53 mg, 73%; yellow oil. ^{1}H NMR (600 MHz, $CDCl_3$): δ 7.68 (m, 2H), 7.62 (dd, *J* = 3.9, 1.0 Hz, 1H), 7.51 (dd, *J* = 5.0, 1.0 Hz, 1H), 7.49 – 7.42 (m, 5H), 7.32 – 7.28 (m, 2H), 7.26 (m, 1H), 7.08 (dd, *J* = 5.0, 3.9 Hz, 1H), 4.20 (q, *J* = 7.2 Hz, 2H), 1.06 (t, *J* = 7.2 Hz, 3H); ^{13}C NMR (151 MHz, $CDCl_3$): δ 168.5, 168.0, 163.9, 158.7, 152.9, 140.8, 137.4, 131.7, 130.4, 130.0, 129.5, 128.6, 128.53, 128.51, 125.4, 121.8, 117.9, 62.5, 13.7.

Ethyl 2-phenoxy-4-phenyl-6-(pyridin-3-yl)pyrimidine-5-carboxylate (**3y**).[16]

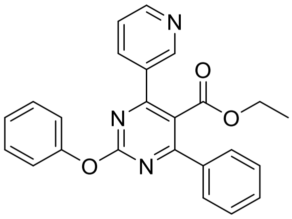

Eluent in chromatography: *n*-hexane/EtOAc 4:1. Yield: 60 mg, 60%; yellow oil. ^{1}H NMR (600 MHz, $CDCl_3$): δ 8.54 (t, *J* = 2.0 Hz, 1H), 8.33 (ddd, *J* = 8.2, 2.2, 0.9 Hz, 1H), 8.02 (m, 1H), 7.69 – 7.61 (m, 3H), 7.51 – 7.47 (m, 1H), 7.47 – 7.42 (m, 4H), 7.32 – 7.23 (m, 3H), 4.11 (q, *J* = 7.2 Hz, 2H), 1.02 (t, *J* = 7.1 Hz, 3H); ^{13}C NMR (151 MHz, $CDCl_3$): δ 168.4, 167.8, 164.9, 164.4, 152.8, 148.3, 138.6, 136.9, 134.7, 130.8, 129.7, 128.7, 128.6, 125.7, 125.1, 123.8, 121.7, 121.1, 62.5, 13.6.

Ethyl 4-(naphthalen-1-yl)-2-phenoxy-6-phenylpyrimidine-5-carboxylate (**3z**).

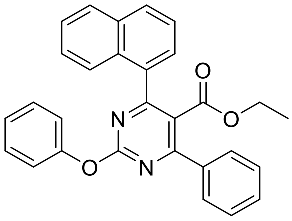

Eluent in chromatography: n-hexane/EtOAc 10:1. Yield: 71 mg, 89%; pale yellow solid, mp 86−88 °C ^{1}H NMR (600 MHz, $CDCl_3$) δ 8.20 (s, 1H), 7.90 (d, *J* = 8.6 Hz, 1H), 7.87 (d, *J* = 8.1 Hz, 2H), 7.76 (dd, *J* = 8.5, 1.6 Hz, 1H), 7.68 (m, 2H), 7.58 – 7.50 (m, 2H), 7.49 – 7.41 (m, 5H), 7.34 – 7.30 (m, 2H),

7.26 – 7.22 (m, 1H), 4.07 (q, *J* = 7.1 Hz, 2H), 0.95 (t, *J* = 7.1 Hz, 3H); ^{13}C NMR (151 MHz, $CDCl_3$) δ 168.4, 167.7, 167.5, 164.3, 153.0, 137.3, 134.5, 134.2, 132.9, 130.5, 129.5, 129.0, 128.9, 128.6, 128.4, 127.9, 127.6, 126.7, 125.5, 125.4, 121.7, 121.3, 62.1, 13.6; IR (film) cm^{-1}: 3053, 2961, 1725, 1536, 1490, 1386, 1257, 697; HRMS (ESI) m/z: $[M + H]^+$ calcd for $C_{29}H_{23}N_2O_3$, 447.1709; found, 447.1733.

Ethyl 2-methoxy-4-methyl-6-phenylpyrimidine-5-carboxylate (**4a**).[19]

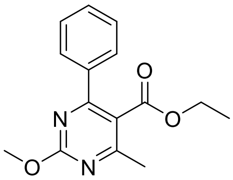

Eluent in chromatography: *n*-hexane/EtOAc 10:1. Yield: 44 mg, 90%; colorless oil. ^{1}H NMR (600 MHz, $CDCl_3$): δ 7.66 – 7.62 (m, 2H), 7.49 – 7.40 (m, 3H), 4.14 (q, *J* = 7.1 Hz, 2H), 4.07 (s, 3H), 2.58 (s, 3H), 1.03 (t, *J* = 7.1 Hz, 3H); ^{13}C NMR (151 MHz, $CDCl_3$): δ 168.8, 168.4, 166.7, 164.5, 137.9, 130.3, 128.5, 128.4, 120.1, 61.8, 55.2, 22.8, 13.7.

Ethyl 2-ethoxy-4-methyl-6-phenylpyrimidine-5-carboxylate (**4b**).[20]

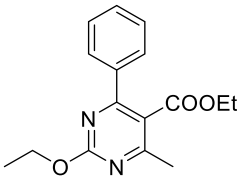

Eluent in chromatography: *n*-hexane/EtOAc 10:1. Yield: 27 mg, 52%; colorless oil. ^{1}H NMR (600 MHz, $CDCl_3$): δ 7.65 – 7.61 (m, 2H), 7.50 – 7.40 (m, 3H), 4.51 (q, *J* = 7.1 Hz, 2H), 4.14 (q, *J* = 7.1 Hz, 2H), 2.57 (s, 3H), 1.45 (t, *J* = 7.1 Hz, 3H), 1.03 (t, *J* = 7.2 Hz, 3H); ^{13}C NMR (151 MHz, $CDCl_3$): δ 168.6, 168.3, 166.8, 164.0, 138.0, 130.3, 128.5, 128.4, 119.9, 64.1, 61.8, 22.7, 14.6, 13.7.

Ethyl 4-methyl-6-phenyl-2-propoxypyrimidine-5-carboxylate (**4c**).[21]

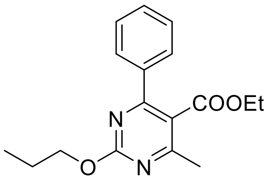

Eluent in chromatography: *n*-hexane/EtOAc 10:1. Yield: 27 mg, 50%; colorless oil.^{1}H NMR (600 MHz, $CDCl_3$) δ 7.65 – 7.61 (m, 2H), 7.48 – 7.39 (m, 3H), 4.40 (t, *J* = 6.7 Hz, 2H), 4.14 (q, *J* = 7.1 Hz, 2H), 2.57 (s, 3H), 1.85 (m, 2H), 1.04 (t, *J* = 7.4 Hz, 3H), 1.02 (t, *J* = 7.2 Hz, 3H); ^{13}C NMR (151 MHz, $CDCl_3$) δ 168.6, 168.3, 166.8, 164.1, 1387.0, 130.3, 128.5, 128.4, 119.9, 69.8, 61.8, 22.7, 22.3, 13.7, 10.6.

Ethyl 2-butoxy-4-methyl-6-phenylpyrimidine-5-carboxylate (**4d**).[21]

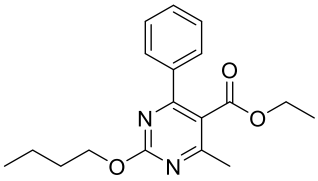

Eluent in chromatography: *n*-hexane/EtOAc 10:1. Yield: 45 mg, 79%; colorless oil.^{1}H NMR (600 MHz, $CDCl_3$) δ 7.64 – 7.61 (m, 2H), 7.47 – 7.41 (m, 3H), 4.45 (t, *J* = 6.6 Hz, 2H), 4.14 (q, *J* = 7.1 Hz, 2H), 2.57 (s, 3H), 1.81 (m, 2H), 1.51 (sext, *J* = 7.5 Hz, 2H), 1.03 (t, *J* = 7.1 Hz, 3H), 0.97 (t, *J* = 7.4 Hz, 3H); ^{13}C NMR (151 MHz, $CDCl_3$) δ 168.7, 168.5, 166.7, 164.4, 138.1, 130.2, 128.5, 128.4, 119.9, 67.9, 61.7, 31.1, 22.9, 19.3, 14.0, 13.7.

Ethyl 2-(benzyloxy)-4-methyl-6-phenylpyrimidine-5-carboxylate (**4e**).[20]

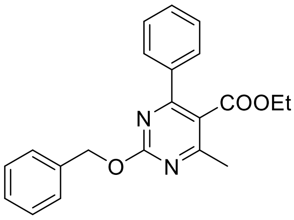

Eluent in chromatography: *n*-hexane/EtOAc 20:1. Yield: 42 mg, 67%; colorless oil. ^{1}H NMR (600 MHz, $CDCl_3$): δ 7.62 (d, *J* = 6.8 Hz, 2H), 7.50 (d, *J* = 7.3 Hz, 2H), 7.48 – 7.41 (m, 3H), 7.36 (t, *J* = 7.4 Hz, 2H), 7.31 (m, 1H), 5.52 (s, 2H), 4.15 (q, *J* = 7.1 Hz, 2H), 2.58 (s, 3H), 1.03 (t, *J* = 7.1 Hz, 3H); ^{13}C NMR (151 MHz, $CDCl_3$): δ 168.8, 168.2, 166.5, 163.9, 137.8, 136.4, 130.1, 128.4, 128.31, 128.29, 128.0, 120.1, 69.3, 61.6, 22.8, 13.6.

Ethyl 4-methyl-2-phenethoxy-6-phenylpyrimidine-5-carboxylate (**4f**)

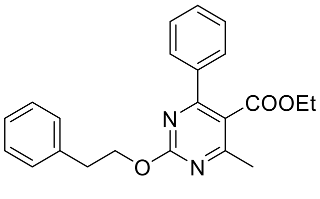

Eluent in chromatography: *n*-hexane/EtOAc 20:1. Yield: 29 mg, 45%; pale yellow oil. ^{1}H NMR (600 MHz, $CDCl_3$) δ 7.62 (m, 2H), 7.48 – 7.41 (m, 3H), 7.31 (d, *J* = 4.4 Hz, 4H), 7.25 – 7.21 (m, 1H), 4.66 (t, *J* = 7.4 Hz, 2H), 4.15 (q, *J* = 7.1 Hz, 2H), 3.16 (t, *J* = 7.4 Hz, 2H), 2.58 (s, 3H), 1.03 (t, *J* = 7.1 Hz, 3H); ^{13}C NMR (151 MHz, $CDCl_3$) δ 168.6, 168.2, 166.9, 163.8, 137.9, 137.8, 130.4, 129.2, 128.7, 128.6, 128.4, 126.7, 120.1, 68.8, 61.8, 35.5, 22.7, 13.7; IR (film) cm^{-1}: 3032, 2923, 1722, 1549, 1427, 1350, 1255, 1069, 698; HRMS (ESI) m/z: $[M + H]^+$ calcd for $C_{22}H_{23}N_2O_3$, 363.1709; found, 363.1684.

Ethyl 2-isopropoxy-4-methyl-6-phenylpyrimidine-5-carboxylate (**4g**).[20]

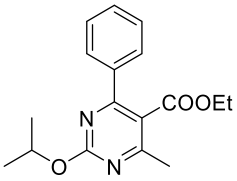

Eluent in chromatography: *n*-hexane/EtOAc 10:1. Yield: 24 mg, 44%; colorless oil. ^{1}H NMR (600 MHz, $CDCl_3$) δ 7.64 (m, 2H), 7.47 – 7.41 (m, 3H), 5.42 (sept, J = 6.2 Hz, 1H), 4.14 (q, J = 7.1 Hz, 2H), 2.58 (s, 3H), 1.42 (d, J = 6.2 Hz, 6H), 1.03 (t, J = 7.2 Hz, 3H); ^{13}C NMR (151 MHz, CD_2Cl_2) δ 168.7, 168.5, 166.7, 163.7, 138.1, 130.2, 128.5, 128.4, 119.6, 71.0, 61.8, 22.8, 22.0, 13.8.

Ethyl 4-methyl-2-phenoxypyrimidine-5-carboxylate (**6**).[22]

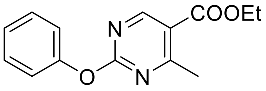

Eluent in chromatography: *n*-hexane/EtOAc 10:1. Yield: 35 mg, 75%; colorless oil. ^{1}H NMR (600 MHz, $CDCl_3$) δ 8.98 (s, 1H), 7.43 (m, 2H), 7.27 (m, 1H), 7.20 (dd, J = 8.6, 1.1 Hz, 2H), 4.37 (q, J = 7.1 Hz, 2H), 2.79 (s, 3H), 1.38 (t, J = 7.2 Hz, 3H); ^{13}C NMR (151 MHz, $CDCl_3$) δ 173.3, 165.6, 164.5, 162.3, 152.6, 129.7, 125.9, 121.7, 119.0, 61.5, 24.8, 14.4.

2-Phenoxypyrimidine (**8**).[23]

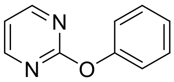

Eluent in chromatography: *n*-hexane/EtOAc 4:1. Yield: 11 mg, 37%; white solid. ^{1}H NMR (600 MHz, CD_2Cl_2) δ 8.53 (d, J = 4.8 Hz, 2H), 7.44 (m, 2H), 7.26 (tt, J = 7.4, 1.1 Hz, 1H), 7.19 (m, 2H), 7.04 (t, J = 4.8 Hz, 1H). ^{13}C NMR (151 MHz, CD_2Cl_2) δ 166.0, 160.2, 153.7, 130.1, 125.8, 122.3, 116.8.

Ethyl 4-methyl-2-(methylthio)-6-phenylpyrimidine-5-carboxylate (**10**).[24]

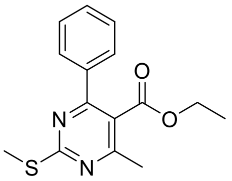

Eluent in chromatography: *n*-hexane/EtOAc 10:1. Yield: 28 mg, 54%; yellow oil. ^{1}H NMR (600 MHz, $CDCl_3$) δ 7.64 (dd, J = 7.9, 1.4 Hz, 2H), 7.45 (m, 3H), 4.15 (q, J = 7.2 Hz, 2H), 2.61 (s, 3H), 2.57 (s, 3H), 1.04 (t, J = 7.2 Hz, 3H); ^{13}C NMR (151 MHz, $CDCl_3$) δ 171.5, 167.1, 164.4, 162.6, 136.8, 129.0, 127.4, 127.3, 119.9, 60.7, 21.6, 13.2, 12.6.

5. Reference

1. Khatri, C. K.; Potadar, S. M.; Chaturbhuj, G. U. *Tetrahedron Lett*. **2017**, *58*, 1778−1780.

2. Chitra, S.; Pandiarajan, K. *Tetrahedron Lett*. **2009**, *50*, 2222−2224.

3. Afradi, M.; Foroughifar, N.; Pasdar, H.; Moghanian, H. *RSC Adv*. **2016**, *6*, 59343−5935.

4. Nishimura Y.; Kikuchi H.; Kubo T.; Gokurakuji Y.; Nakamura, Y.; Arai R.; Yuan B.; Sunaga, K.; Cho, H. *Tetrahedron Lett*. **2020**, *61*, 151967.

5. Sari, O.; Roy, V.; Metifiot, M.; Marchand, C.; Pommier, Y.; Bourg, S.; Bonnet, P.; Schinazi, R. F.; Agrofoglio, L. A. *Eur. J. Med. Chem. 2015*, **104**, 127−138.

6. Hayashi, M.; Okunaga, K. − I.; Nishida, S.; Kawamura, K.; Eda, K. *Tetrahedron Lett*. **2010**, *51*, 6734−6736.

7. Lokwani, D.; Azad, R.; Sarkate, A.; Reddanna, P.; Shinde, D. *Bioorg. Med. Chem*. **2015**, *23*, 4533−4543.

8. Nemr, M. T. M.; AboulMagd, A. M. *Bioorg. Chem*. **2020**, *103*, 104134.

9. Pham, N. S. L.; Shin, H.; Kang, J. Y.; Sohn, J. − H. *J. Org. Chem.* **2020**, *85*, 5087−5096.

10. Yang, H.; Pham, N. S. L.; Shin, H.; Sohn, J. − H. *Bull. Korean Chem. Soc.* **2020**, *41*, 881−883.

11. Phan, N. H. T.; Kim, H.; Shin, H.; Lee, H. -S.; Sohn, J. -H. *Org. Lett.* **2016**, *18*, 5154−5157.

12. Gartner, M.; Sunder-Plassmann, N.; Seiler, J.; Utz, M.; Vernos, I.; Surrey, T.; Giannis, A. *ChemBioChem*, **2005**, *6*, 1173-1177.

13. Jenner, G. *Tetrahedron Lett*. **2004**, *45*, 6195-6198.

14. Thorat, P. B.; Waghmode, N. A.; Karade, N. N. *Tetrahedron Lett*. **2014**, *55*, 5718-5721.

15. Zhang P.; Guo Y.; Quan Z. J. *Heteroatom Chem.* **2017**, *28*, e21397

16. Phan, N. H. T.; Lee, J.; Shin, H.; Sohn, J, -H. *J. Org. Chem.* **2021**, *86*, 5423-5430.

17. Quan, Z.; Jing, F.; Zhang, Z.; Da, Y.; Wang, X. *Chin. J. Chem*. **2013**, *31*, 1495-1502

18. Kang, F. A.; Kodah, J.; Guan, Q.; Li, X.; Murray, W. V. *J. Org. Chem*. **2005**, *70*, 1957-1960.

19. Yammoto, K.; Chen, Y. G.; Buono, F. G. *Org. Lett*, **2005**, *7*, 4673 – 4676.

20. Wang, X. –C.; Yang, G. –J.; Jia, X. -D.; Zhang, Z; Da, Y. –X.; Quan, Z, -J. *Tetrahedron* **2011**, *67*, 3267-3272.

21. Kim, H.; Lee, J.; Shin, H.; Sohn, J.-H. *Org. Lett*. **2018**, *20*, 1961−1965.

22. Abe, T.; Tamai, R.; Ito, M.; Tamaru, M.; Yano, H.; Takahashi, S.; Muramatsu, N. WO Patent No. 2003029211, 2003.

23. Platon, M.; Cui, L.; Mom, S.; Richard, P.; Saeys, M.; Hierso, J. –C. *Adv. Synth. Catal*. **2011**, *353*, 3403-3414.

24. Phan, N. H. T.; Sohn, J, -H. *Tetrahedron.* **2014**, *70*, 7929-7935.

6. ^{1}H and ^{13}C NMR spectra of 1e, 3, 4, 5, 6, 8 and 10

Phenyl 6-methyl-4-phenyl-2-thioxo-1,2,3,4-tetrahydropyrimidine-5-carboxylate (**1e**)

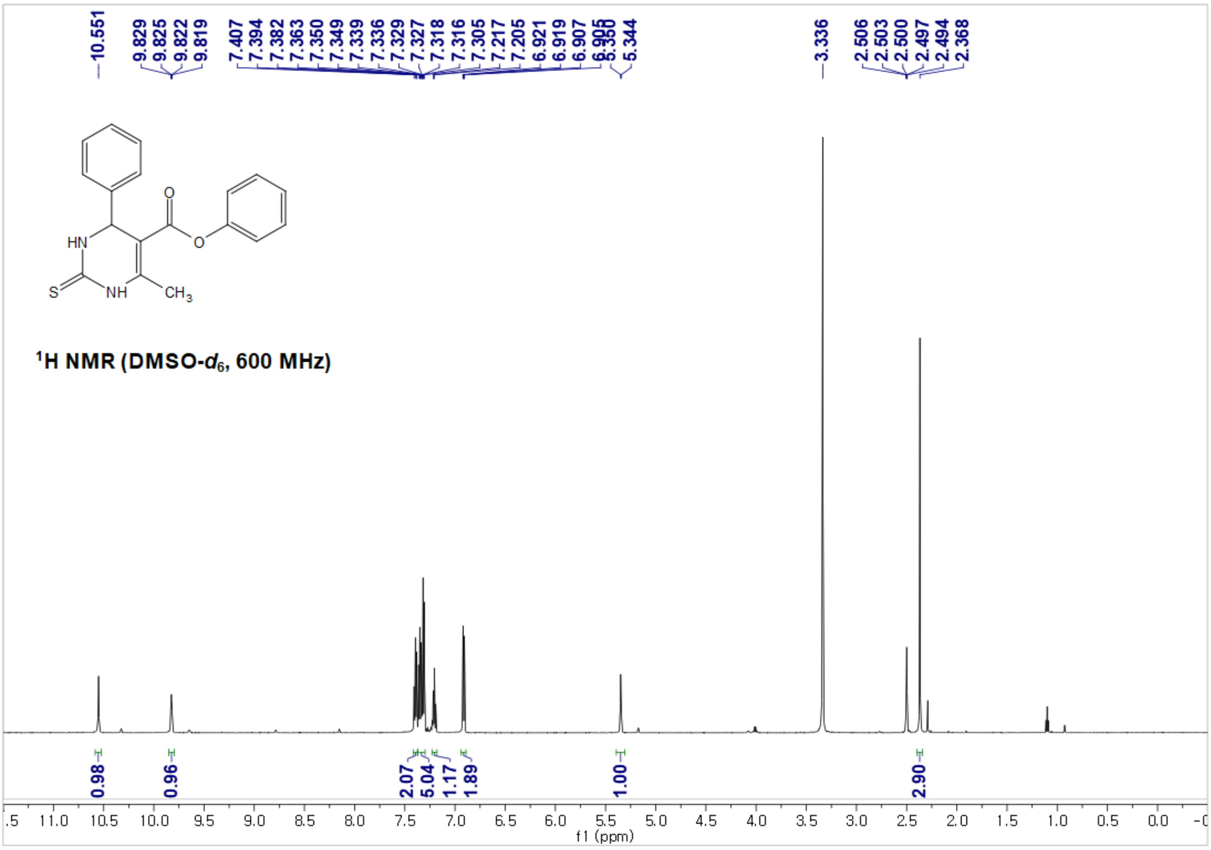

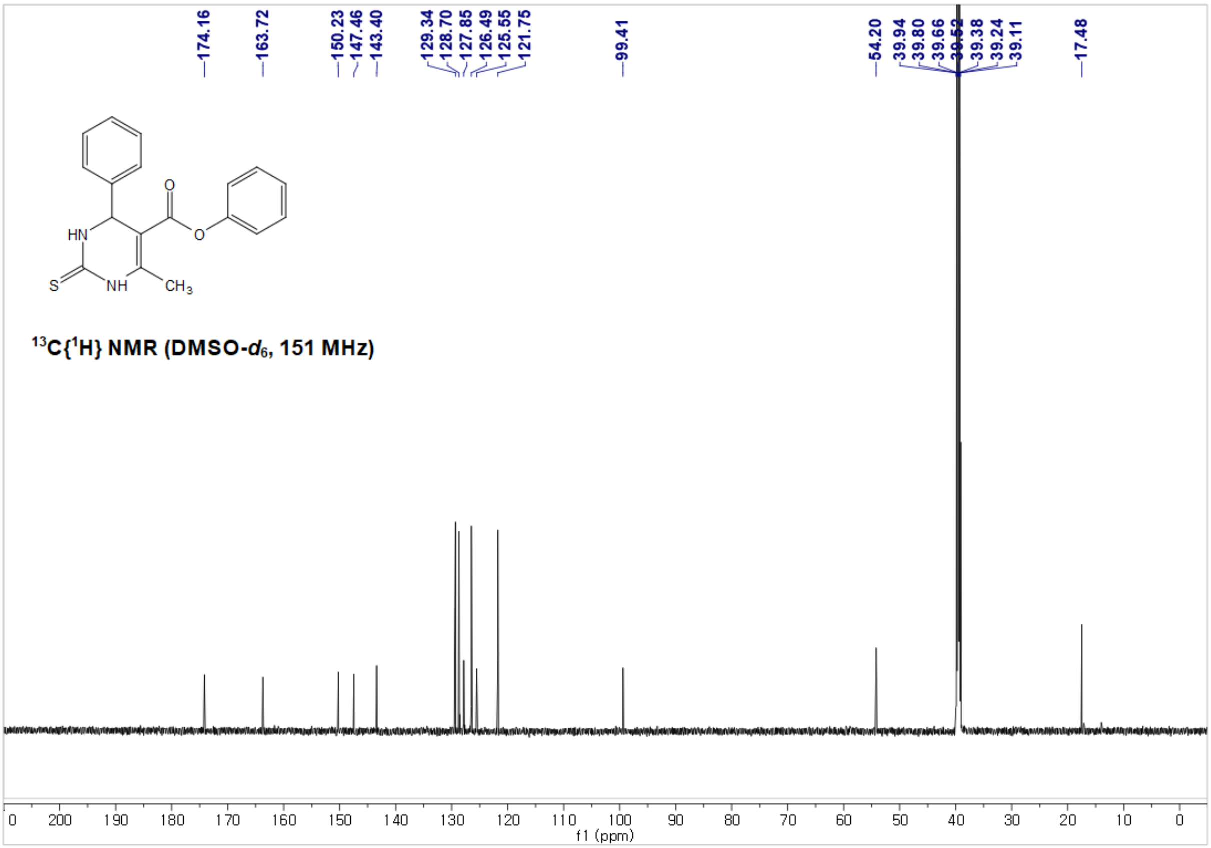

Ethyl 4-(tert-butyl)-6-methyl-2-thioxo-1,2,3,4-tetrahydropyrimidine-5-carboxylate (**5**).

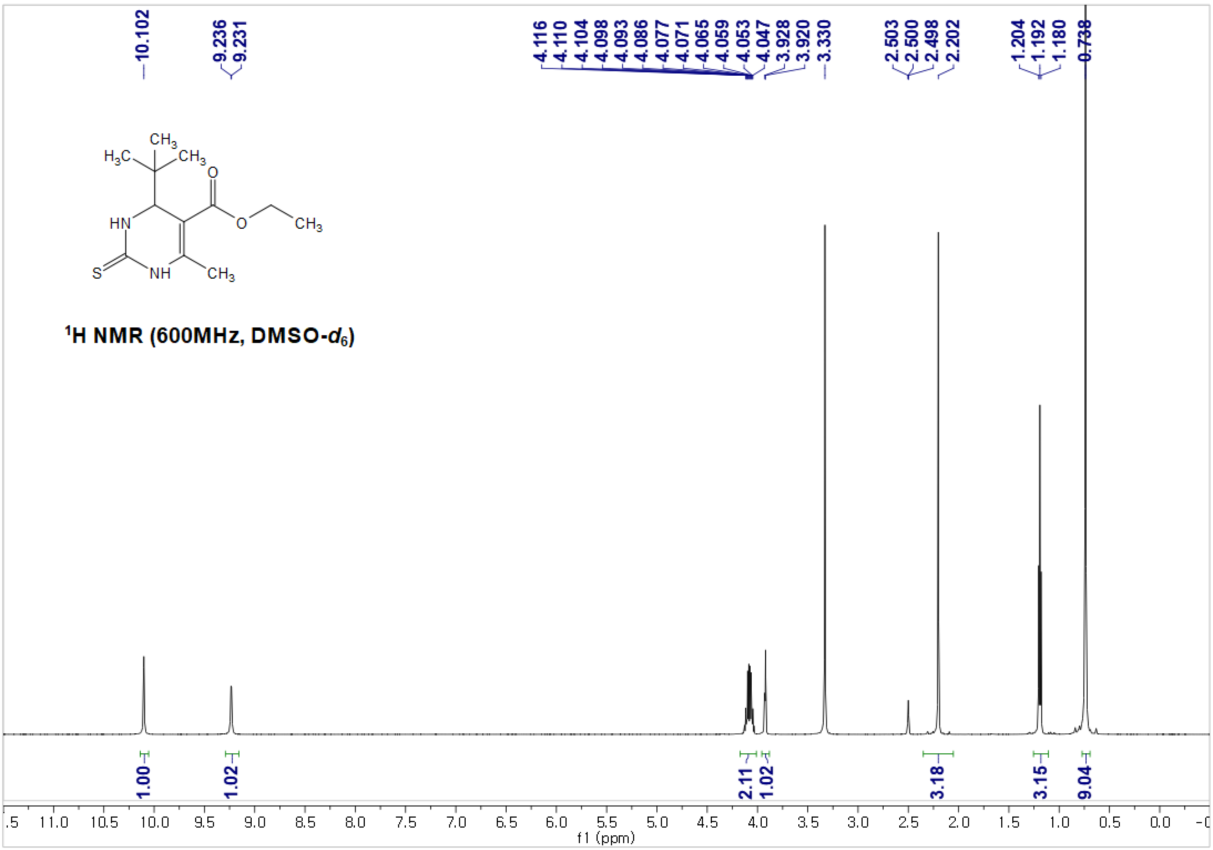

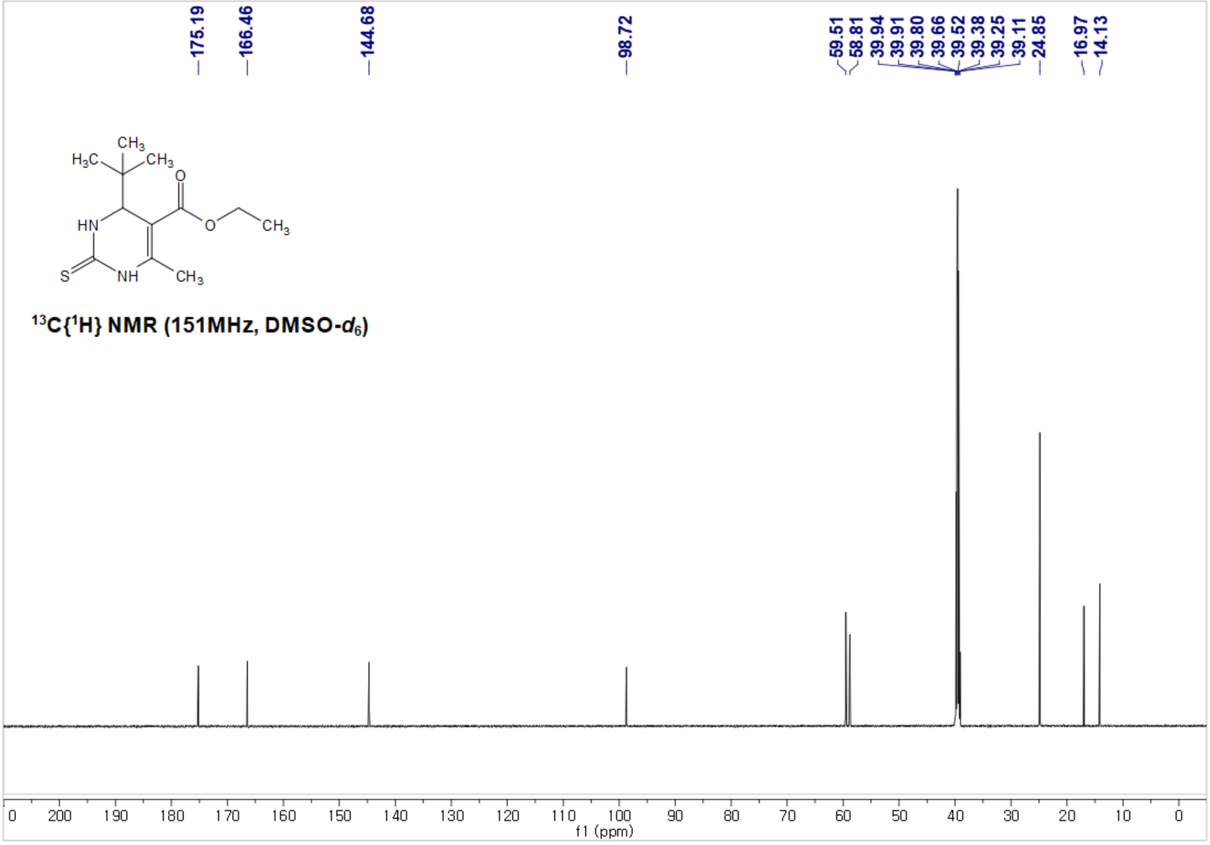

Ethyl 4-methyl-2-phenoxy-6-phenylpyrimidine-5-carboxylate (**3a**).

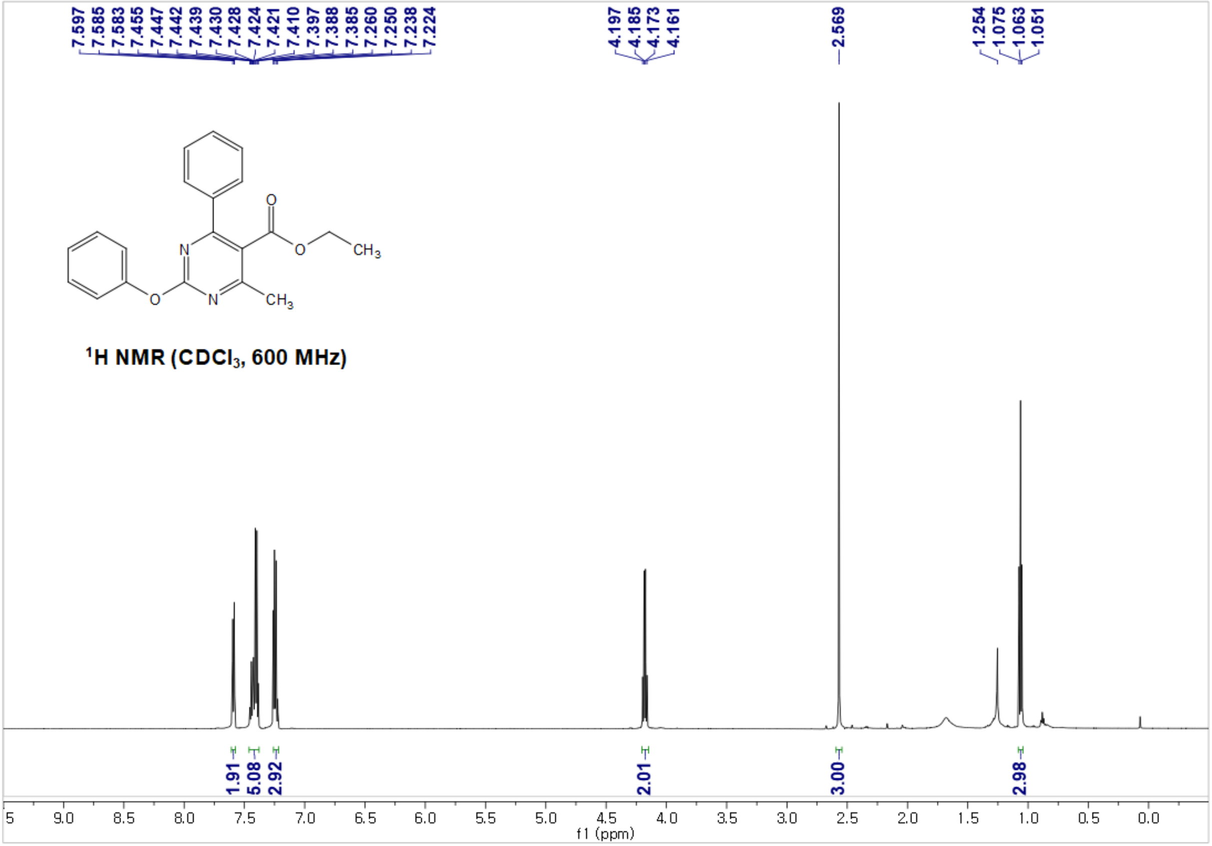

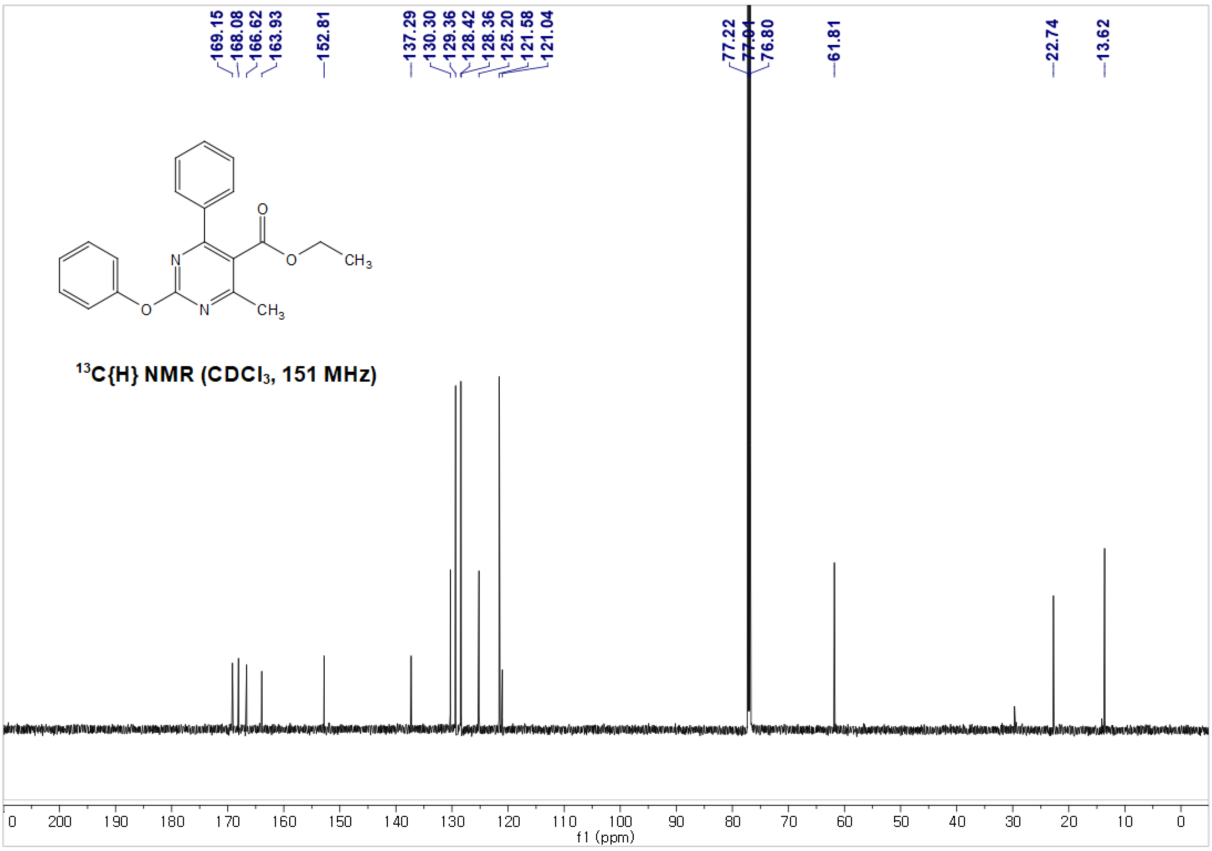

Ethyl 4-methyl-6-phenyl-2-(p-tolyloxy)pyrimidine-5-carboxylate (**3b**).

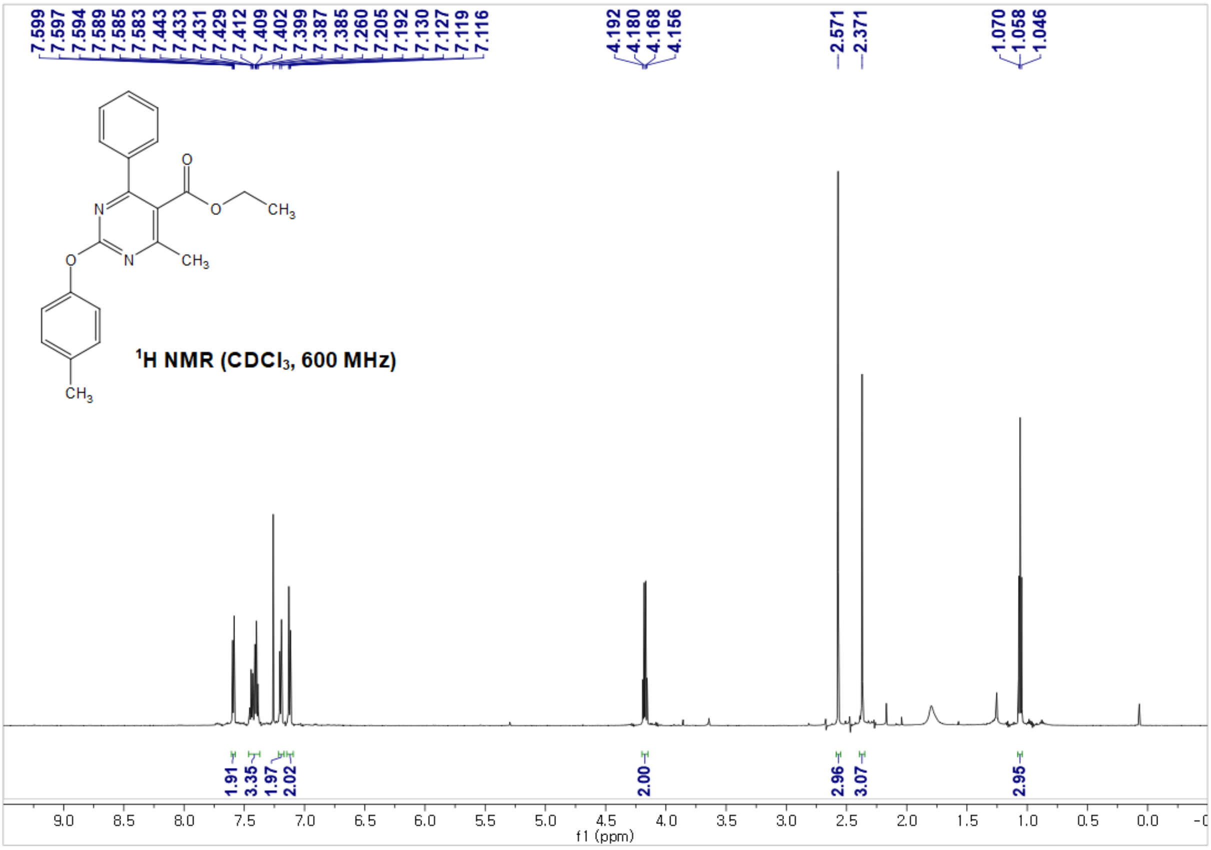

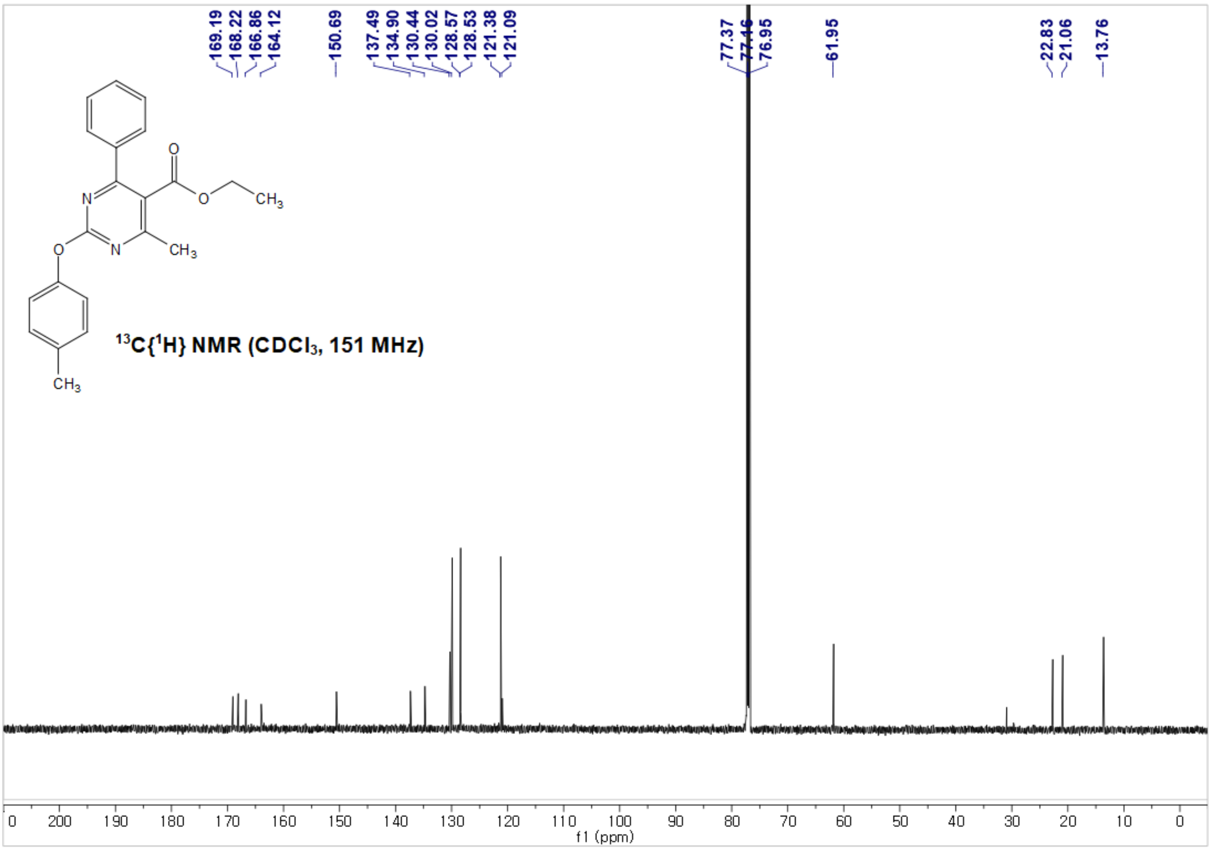

Ethyl 2-(4-chlorophenoxy)-4-methyl-6-phenylpyrimidine-5-carboxylate (**3c**).

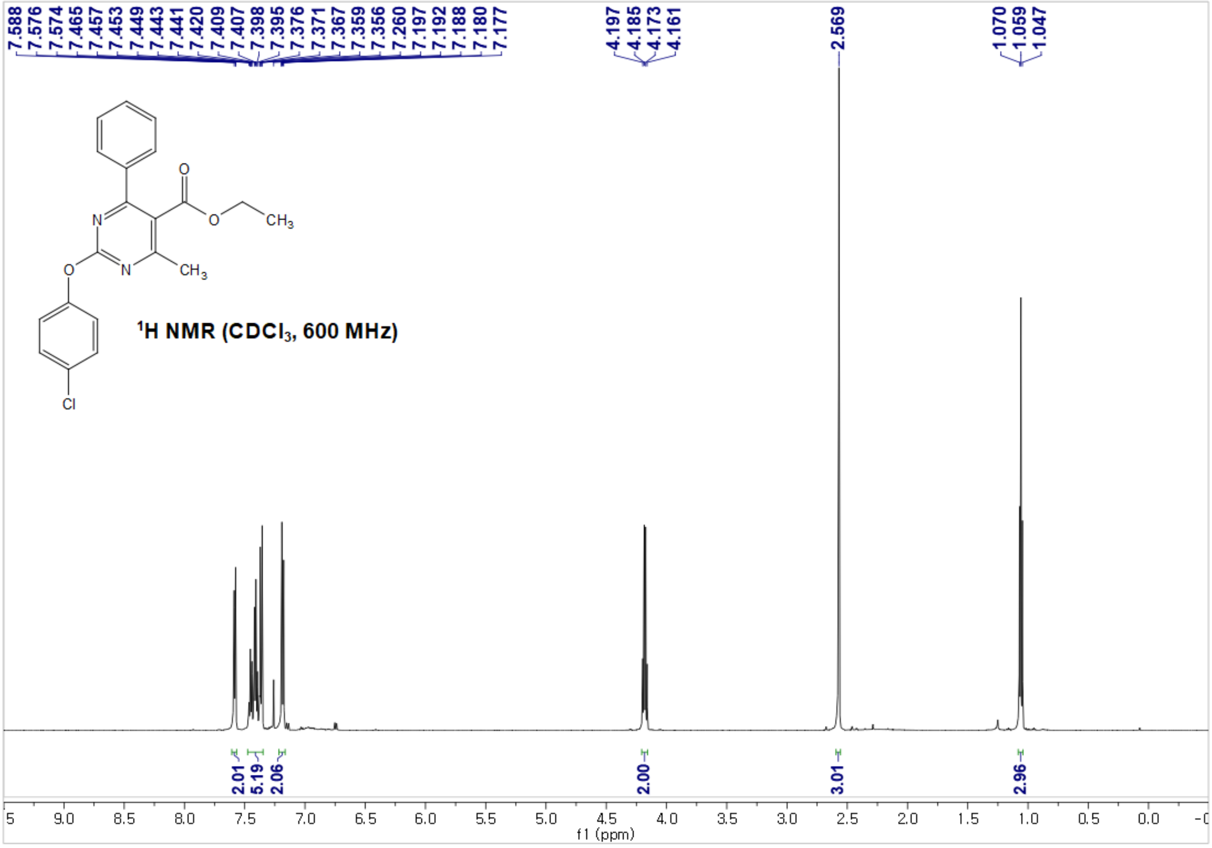

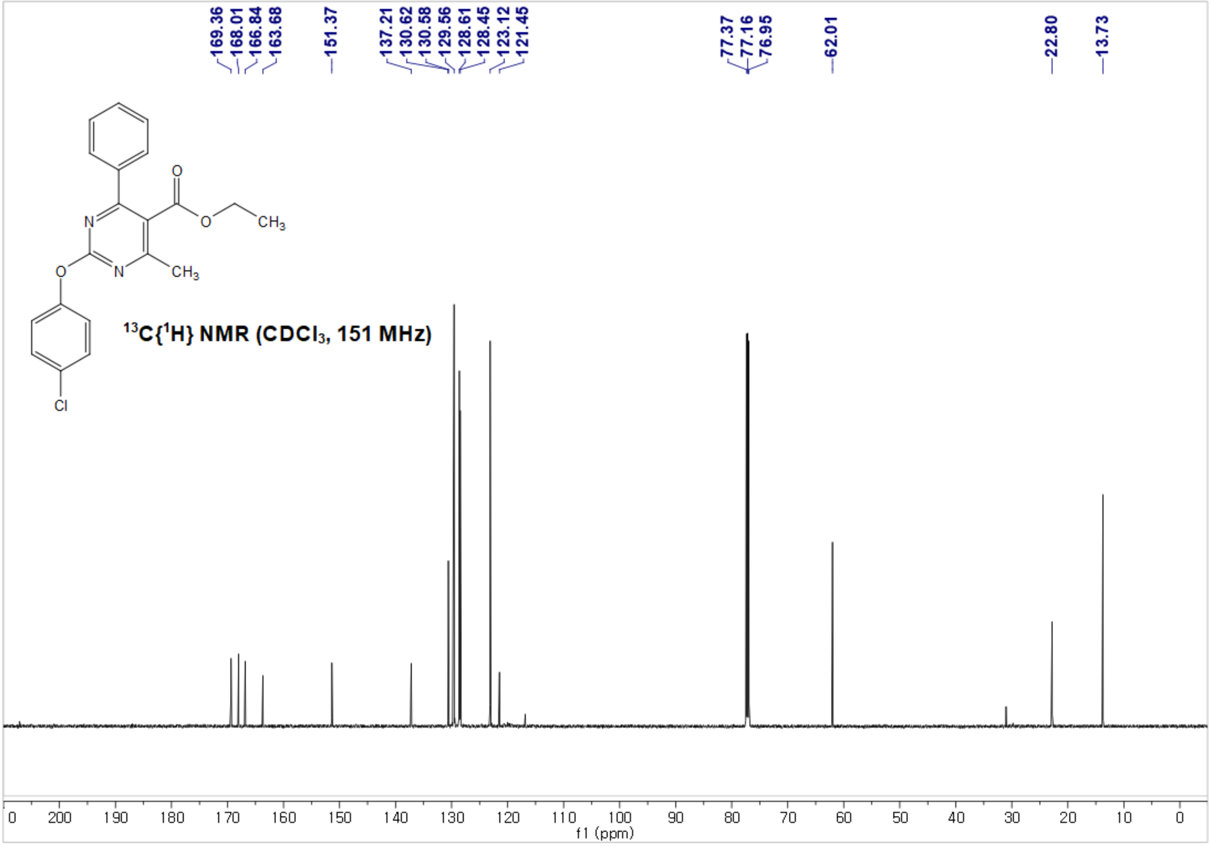

Ethyl 2-(4-bromophenoxy)-4-methyl-6-phenylpyrimidine-5-carboxylate (**3d**).

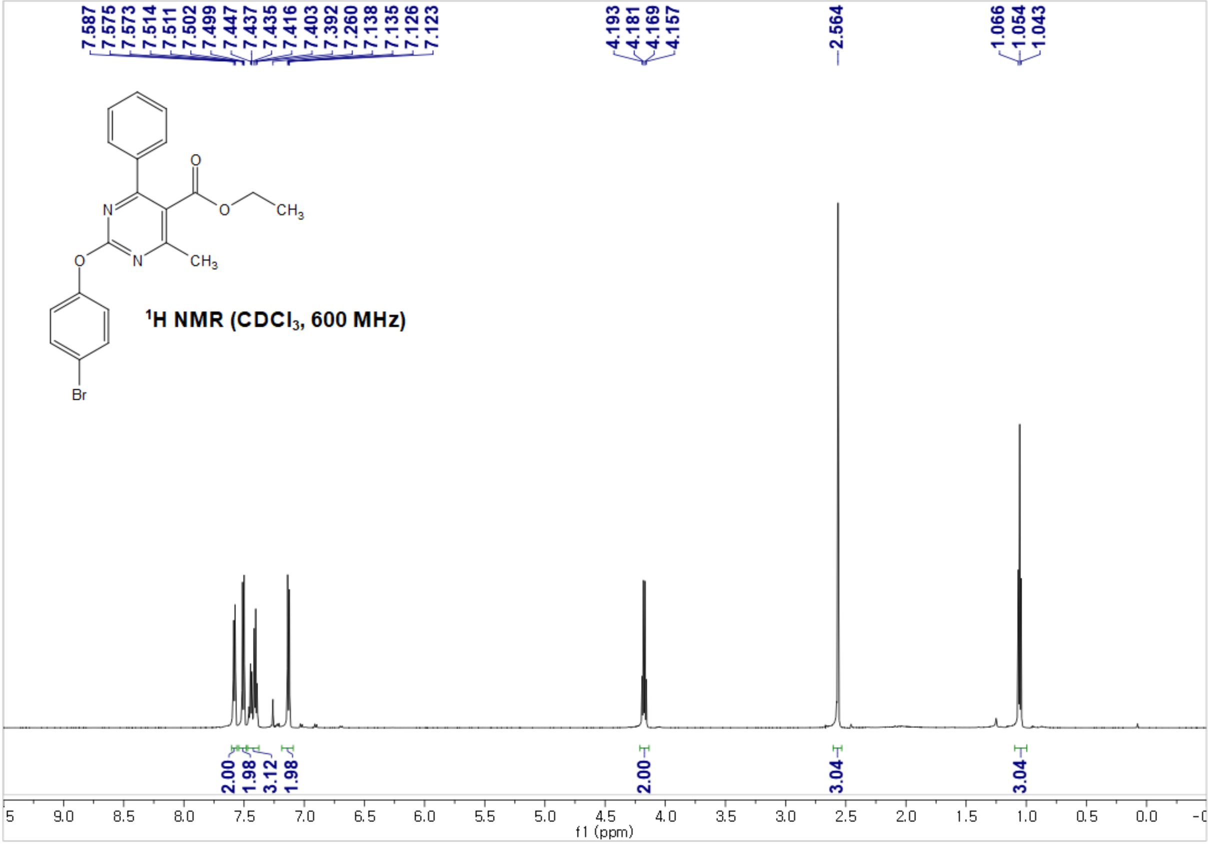

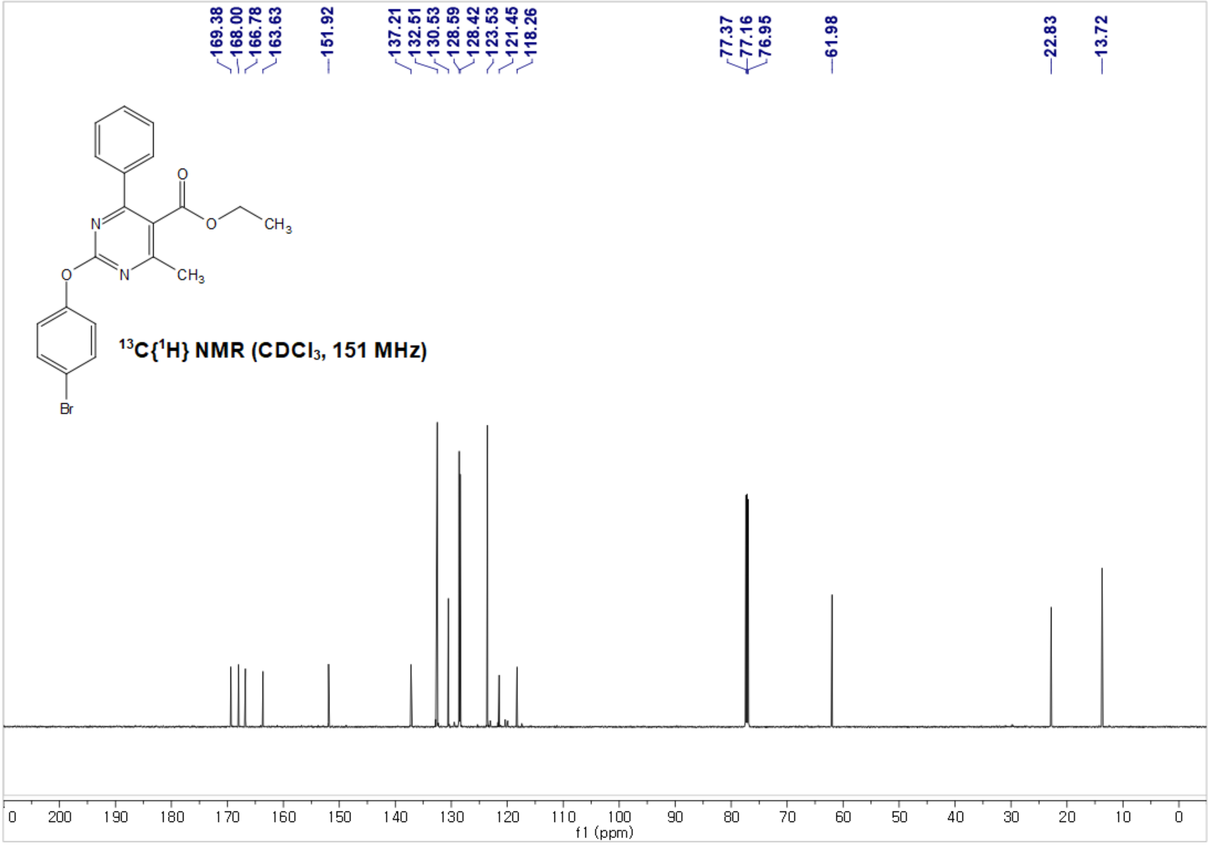

Ethyl 2-(4-iodophenoxy)-4-methyl-6-phenylpyrimidine-5-carboxylate (**3e**).

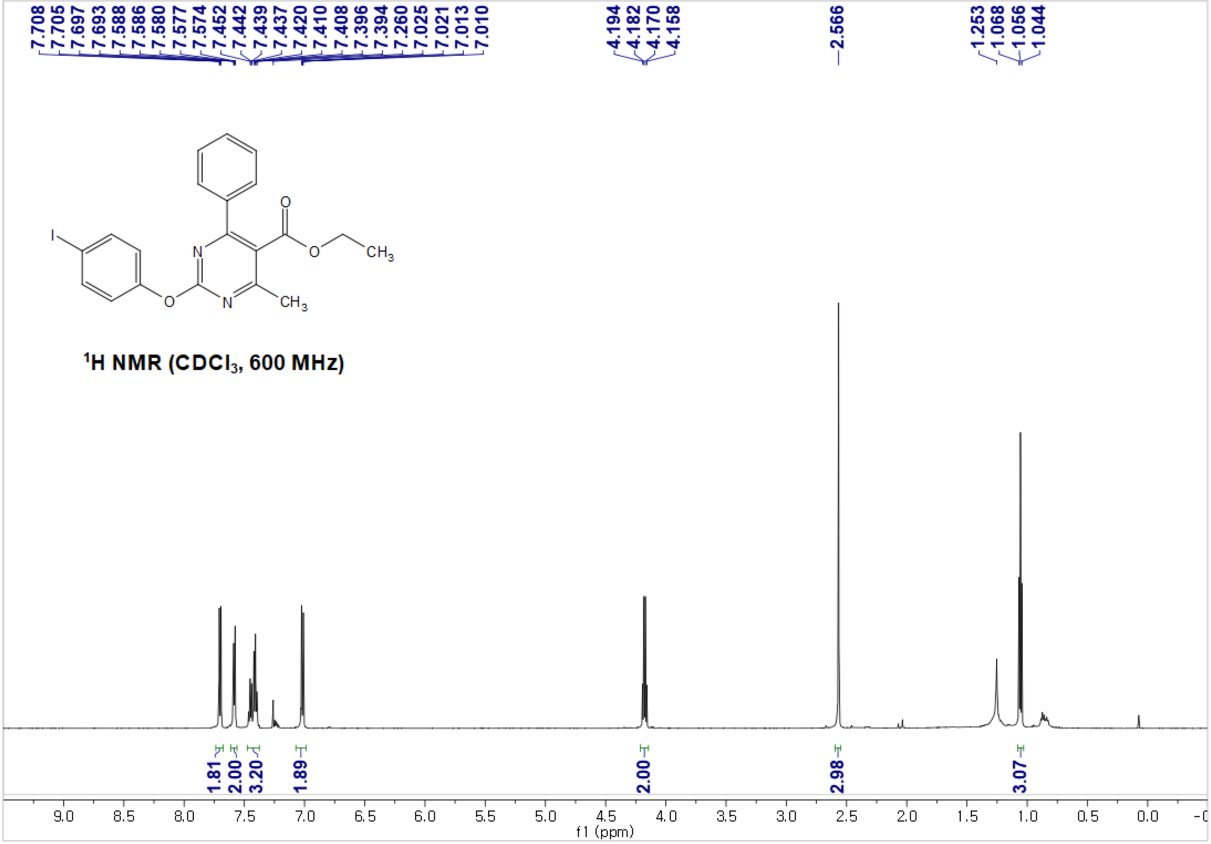

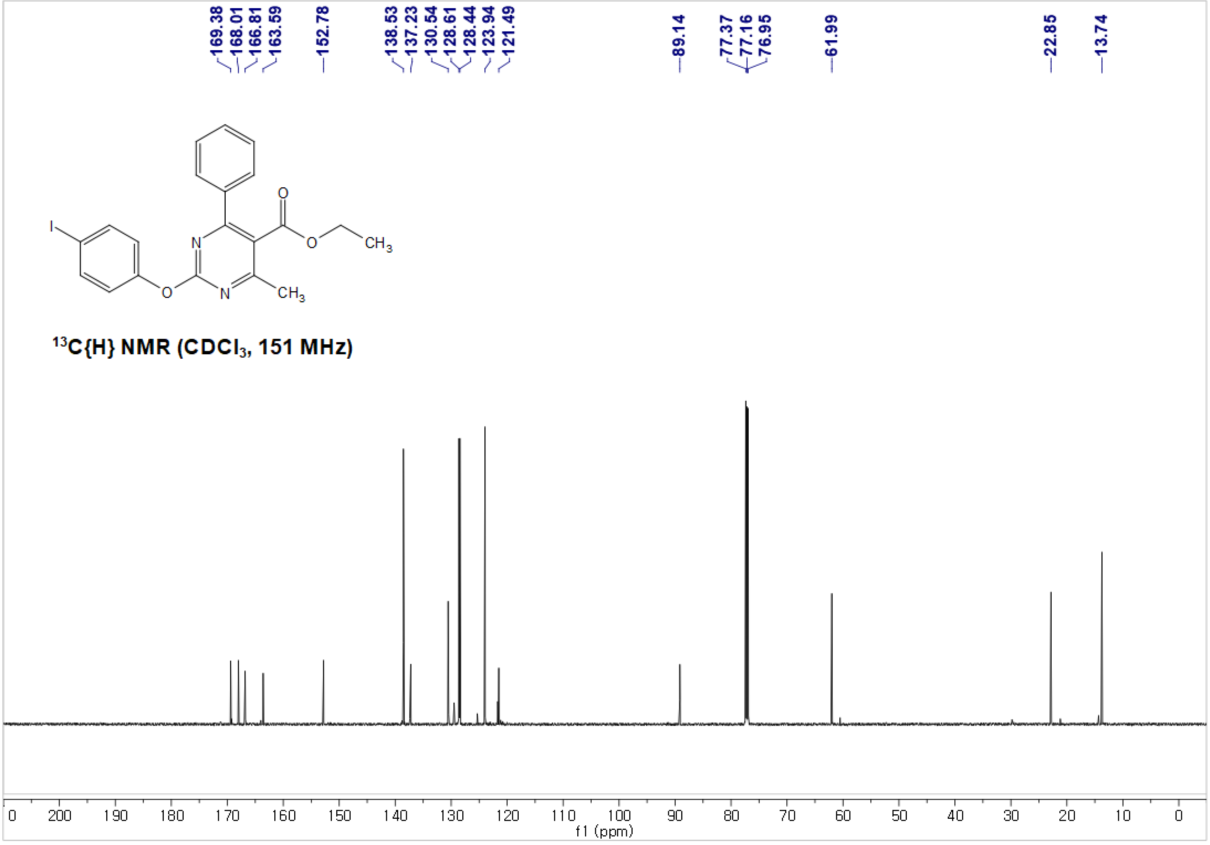

Ethyl 2-(4-cyanophenoxy)-4-methyl-6-phenylpyrimidine-5-carboxylate (**3f**).

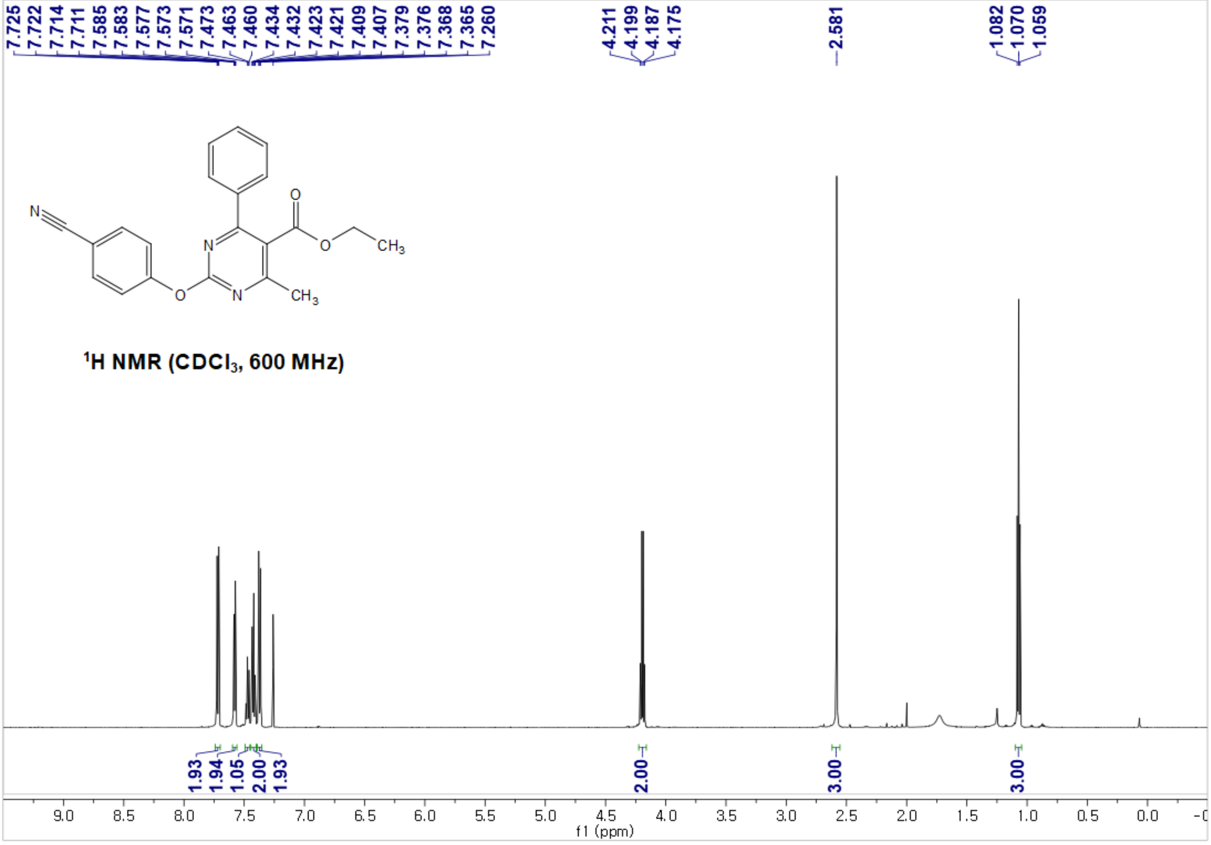

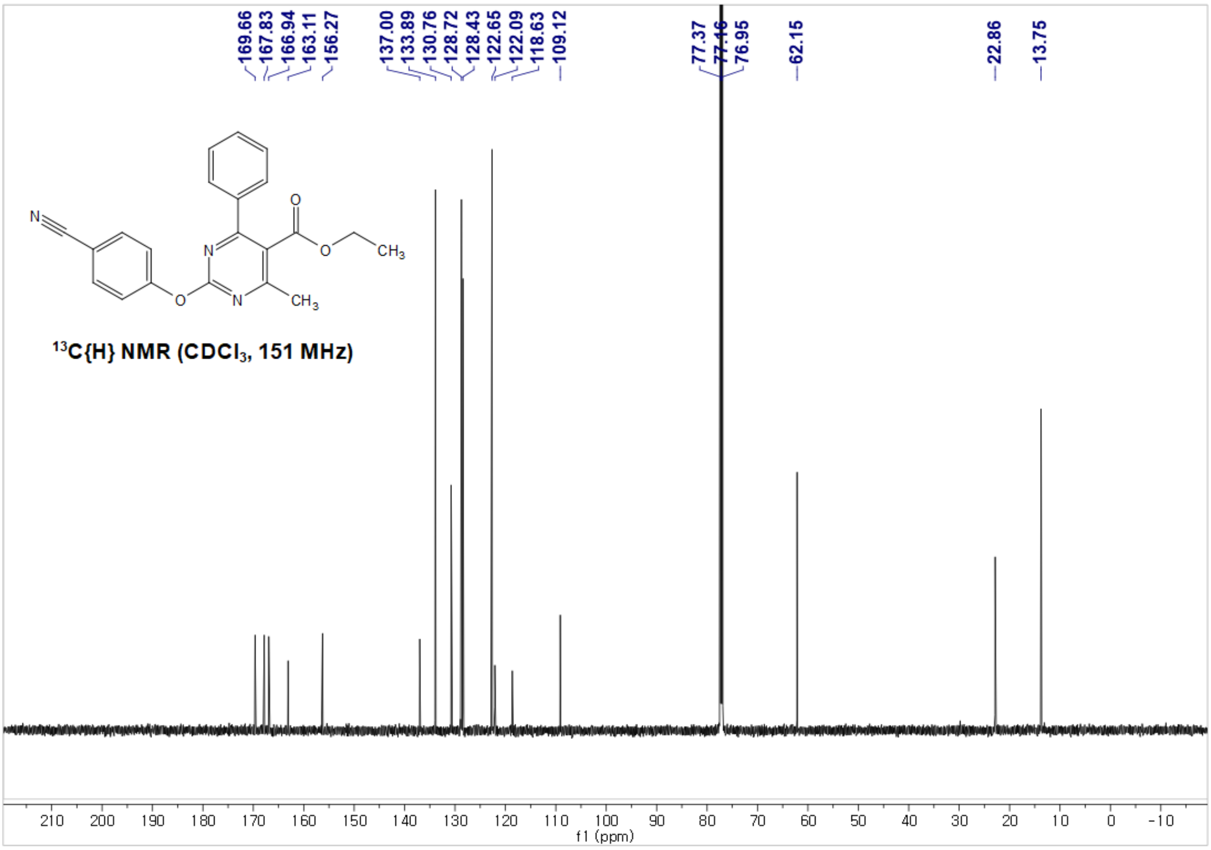

Ethyl 4-methyl-2-(4-nitrophenoxy)-6-phenylpyrimidine-5-carboxylate (**3g**).

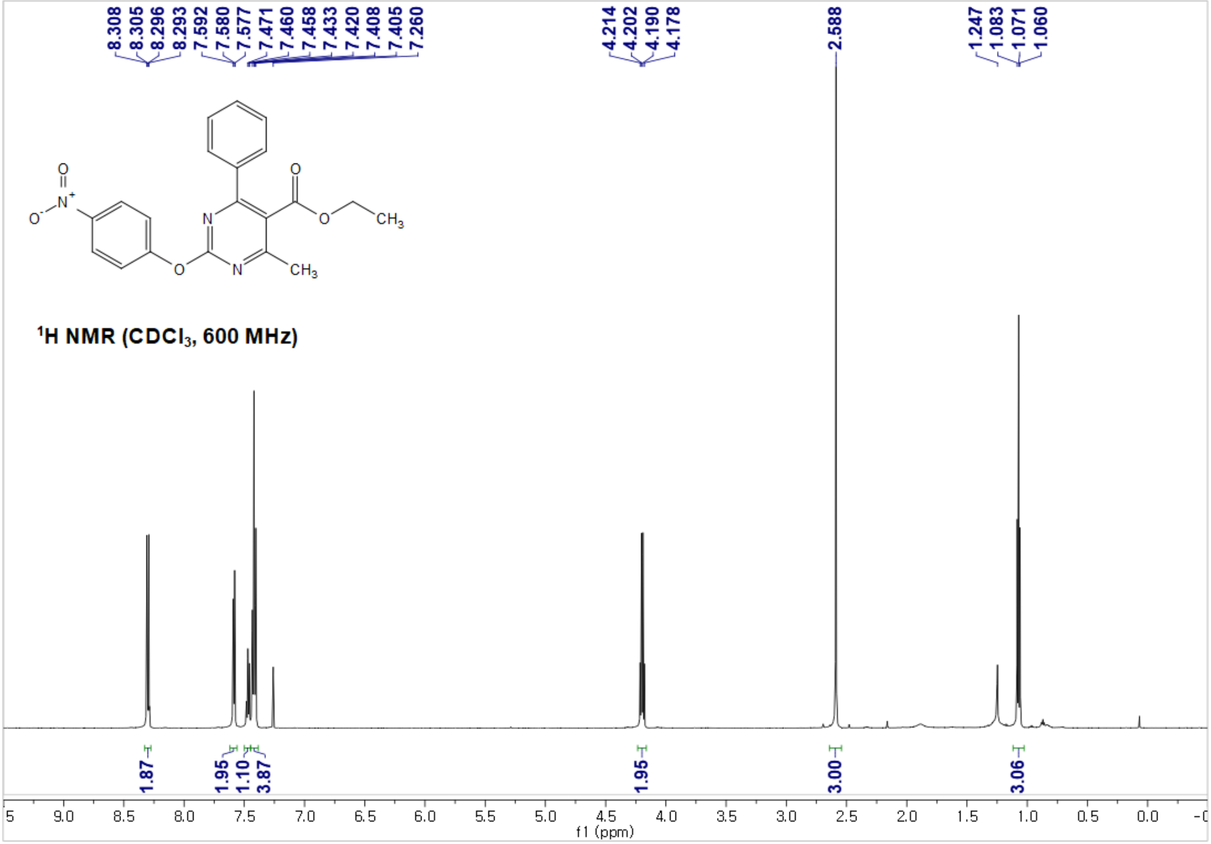

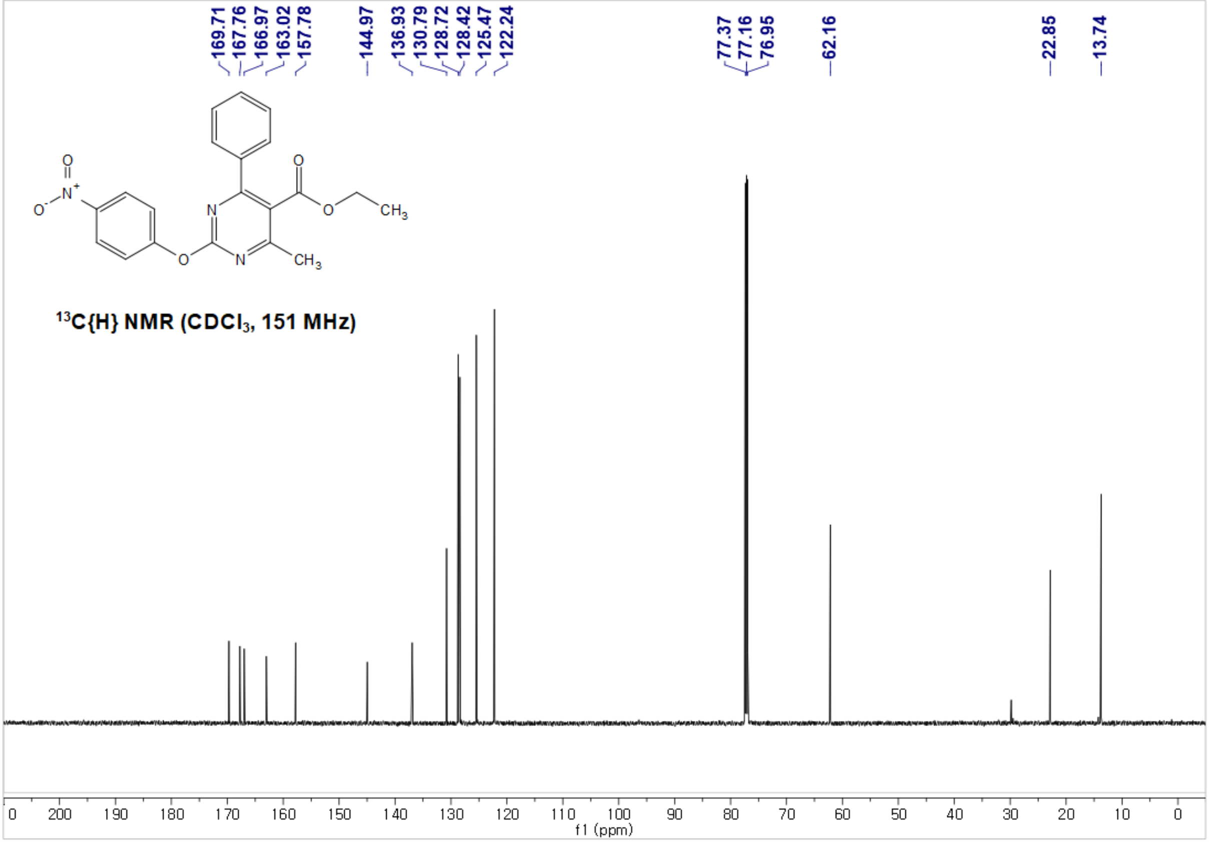

Ethyl 2-(4-formylphenoxy)-4-methyl-6-phenylpyrimidine-5-carboxylate (**3h**).

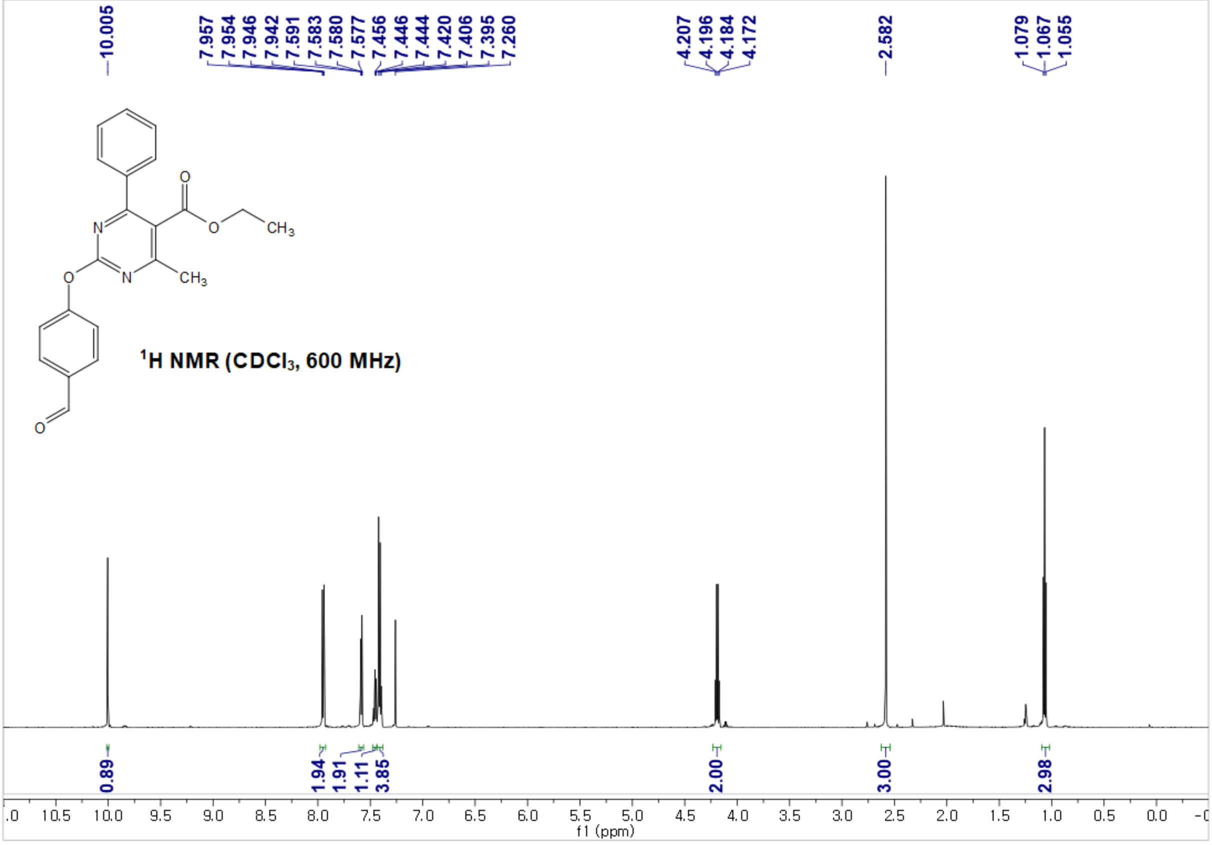

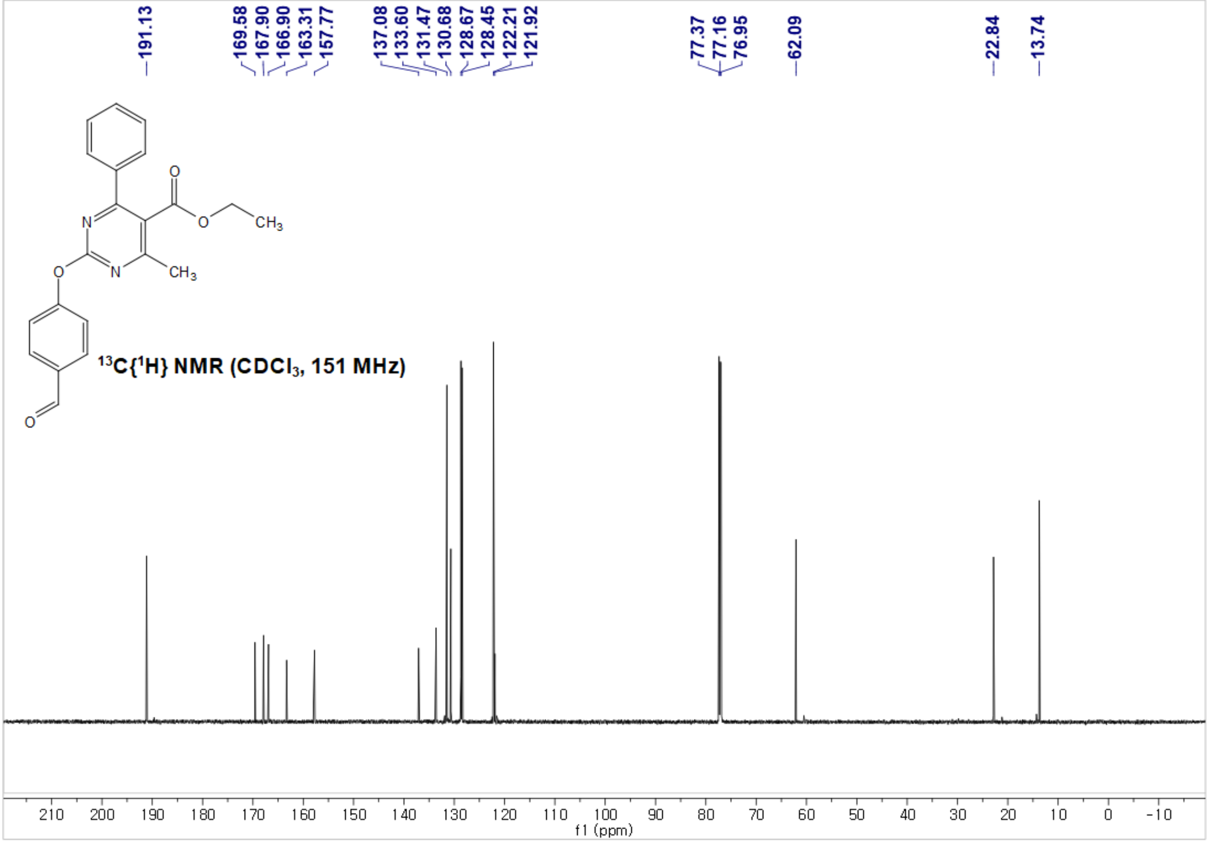

Methyl 4-methyl-2-phenoxy-6-phenylpyrimidine-5-carboxylate (**3i**).

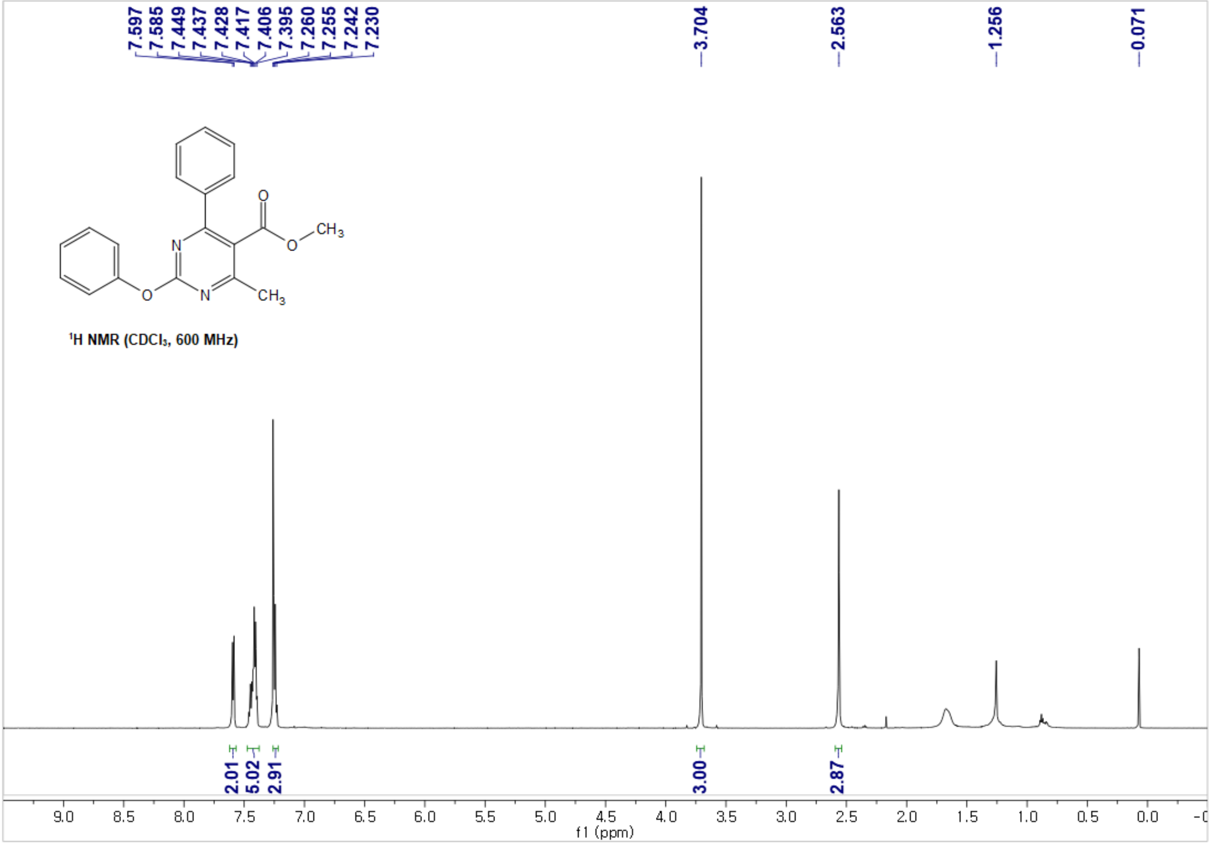

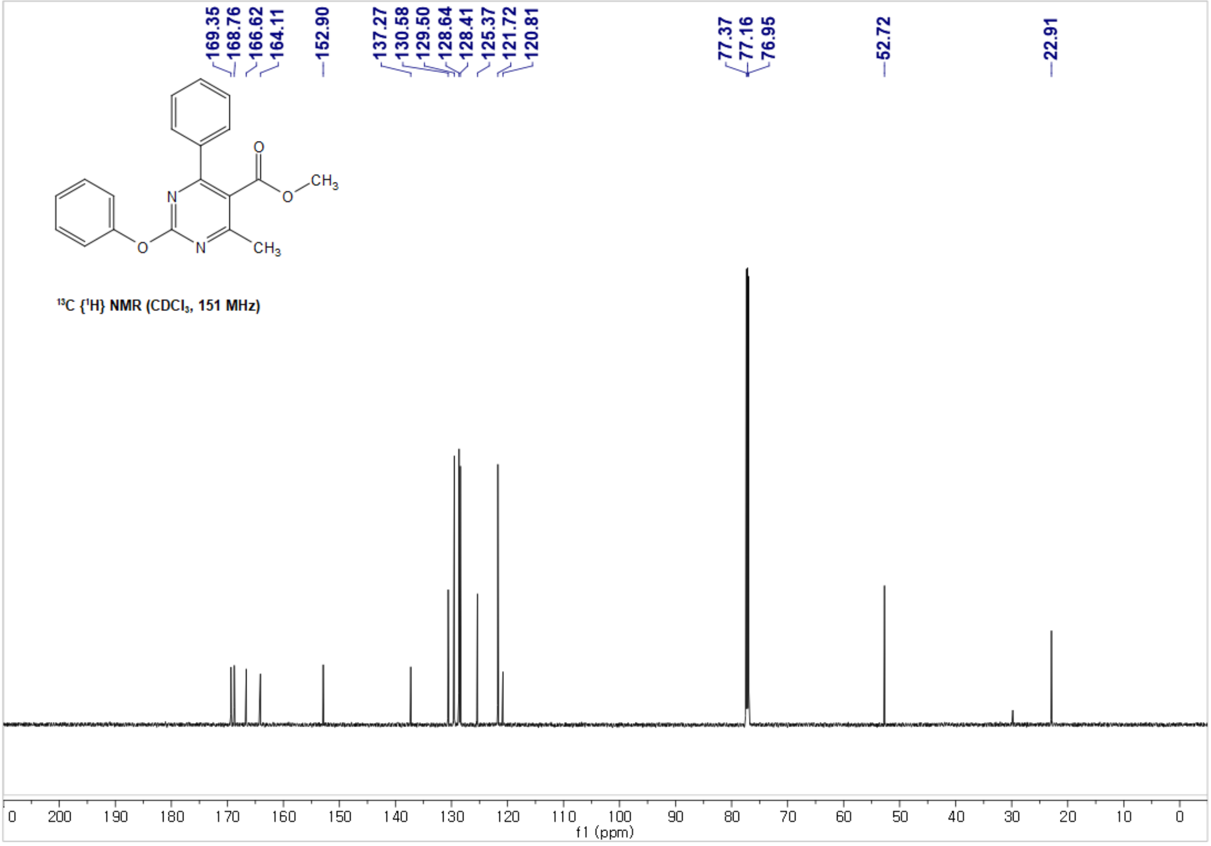

Isopropyl 4-methyl-2-phenoxy-6-phenylpyrimidine-5-carboxylate (**3j**).

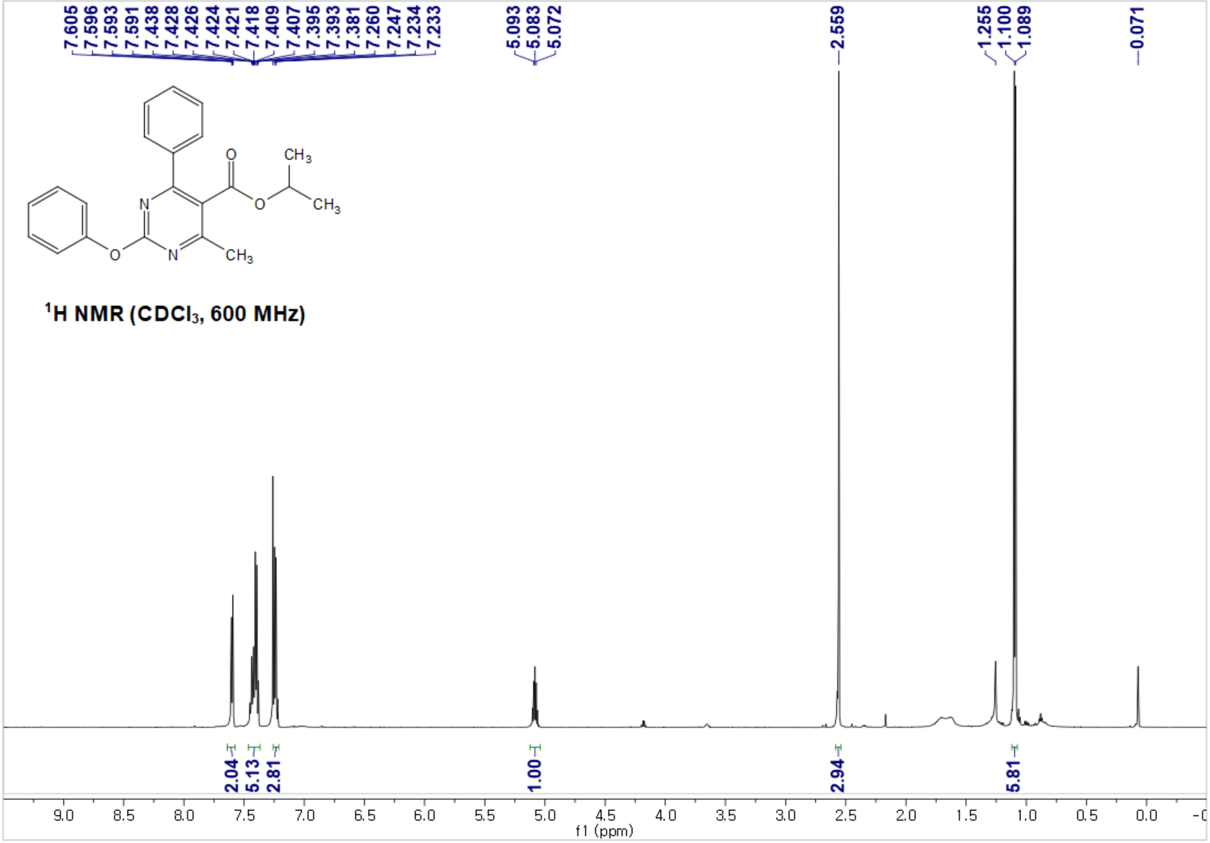

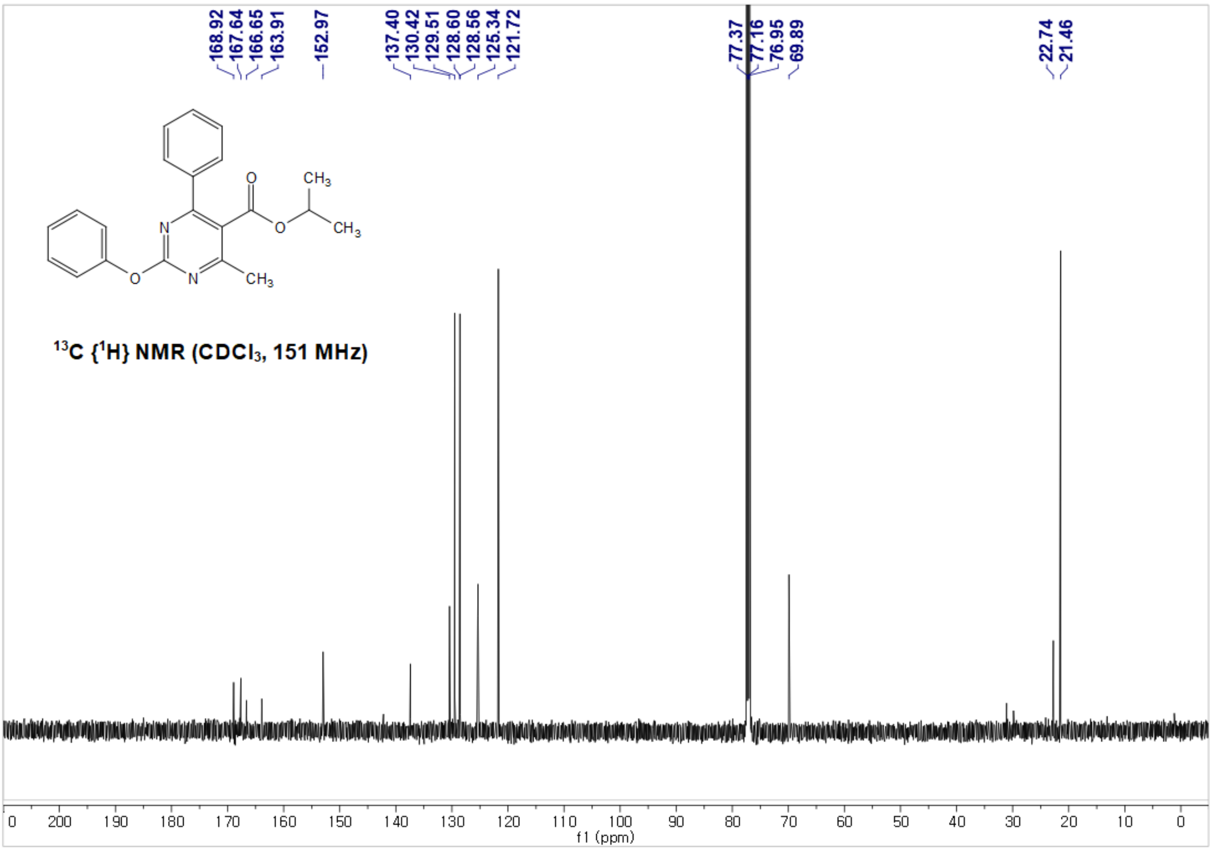

Tert-butyl 4-methyl-2-phenoxy-6-phenylpyrimidine-5-carboxylate (**3k**).

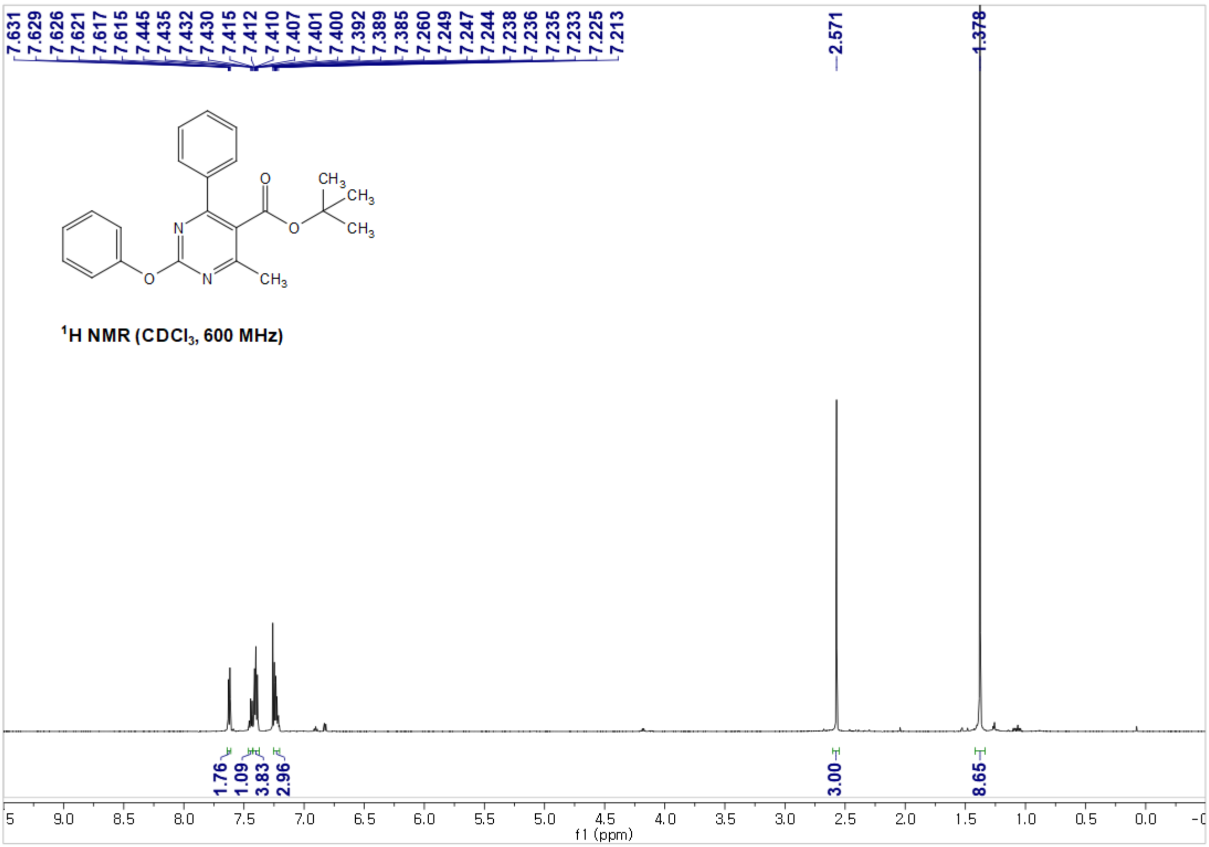

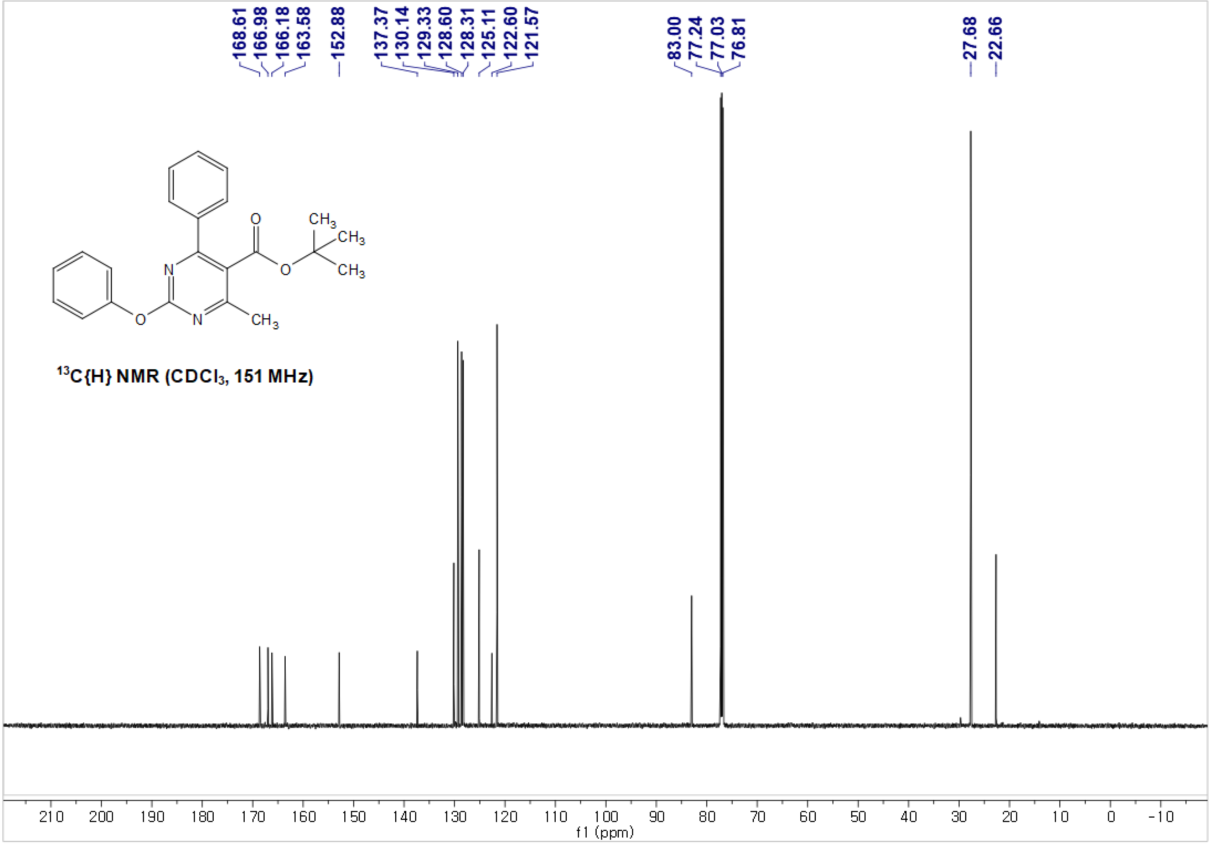

Phenyl 4-methyl-2-phenoxy-6-phenylpyrimidine-5-carboxylate (**3l**).

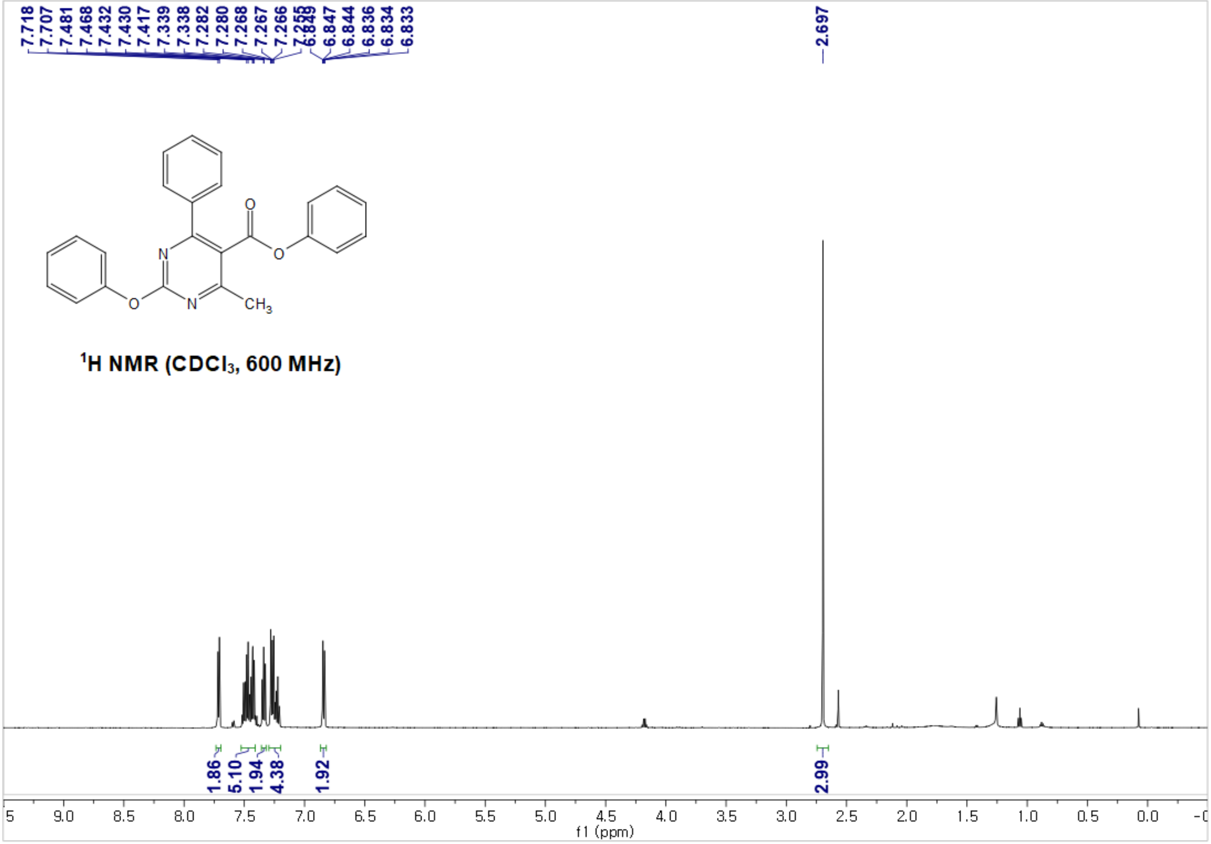

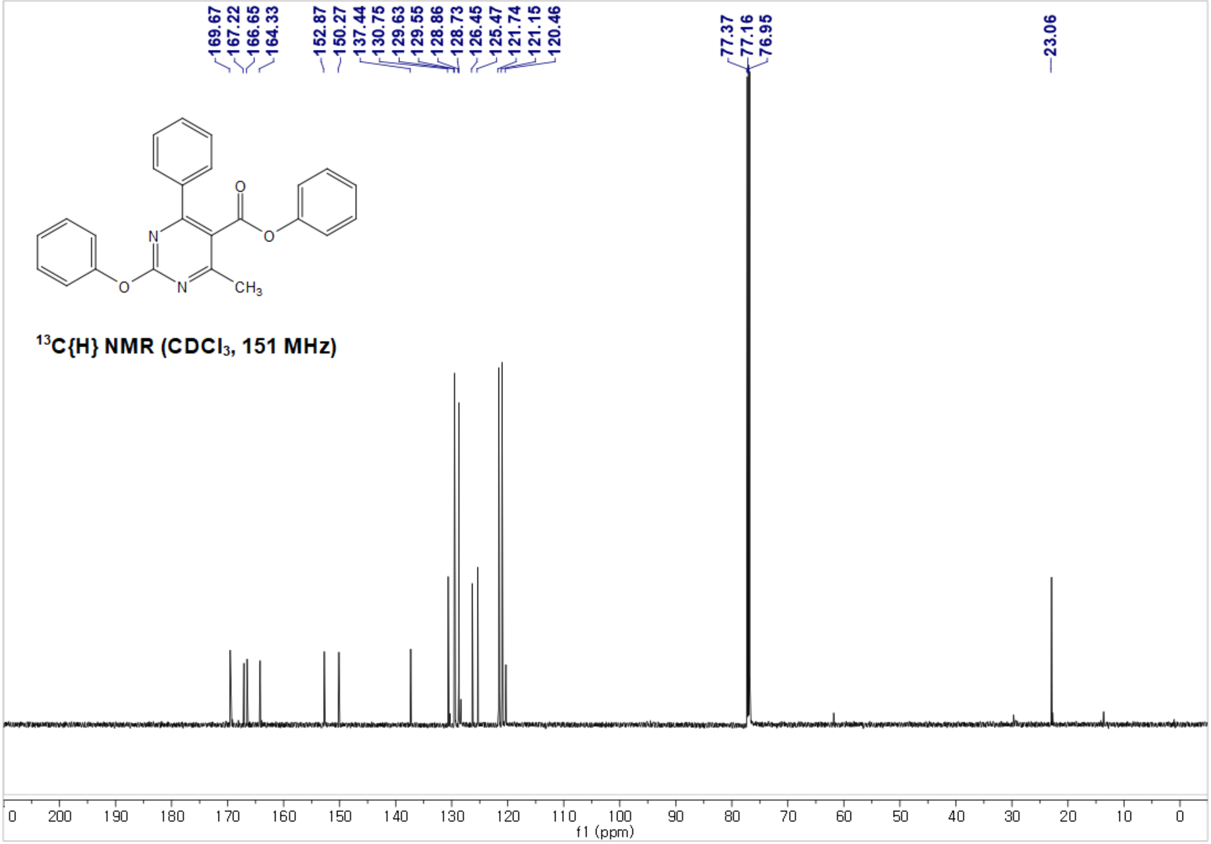

Ethyl 2-phenoxy-4-phenylpyrimidine-5-carboxylate (**3m**).

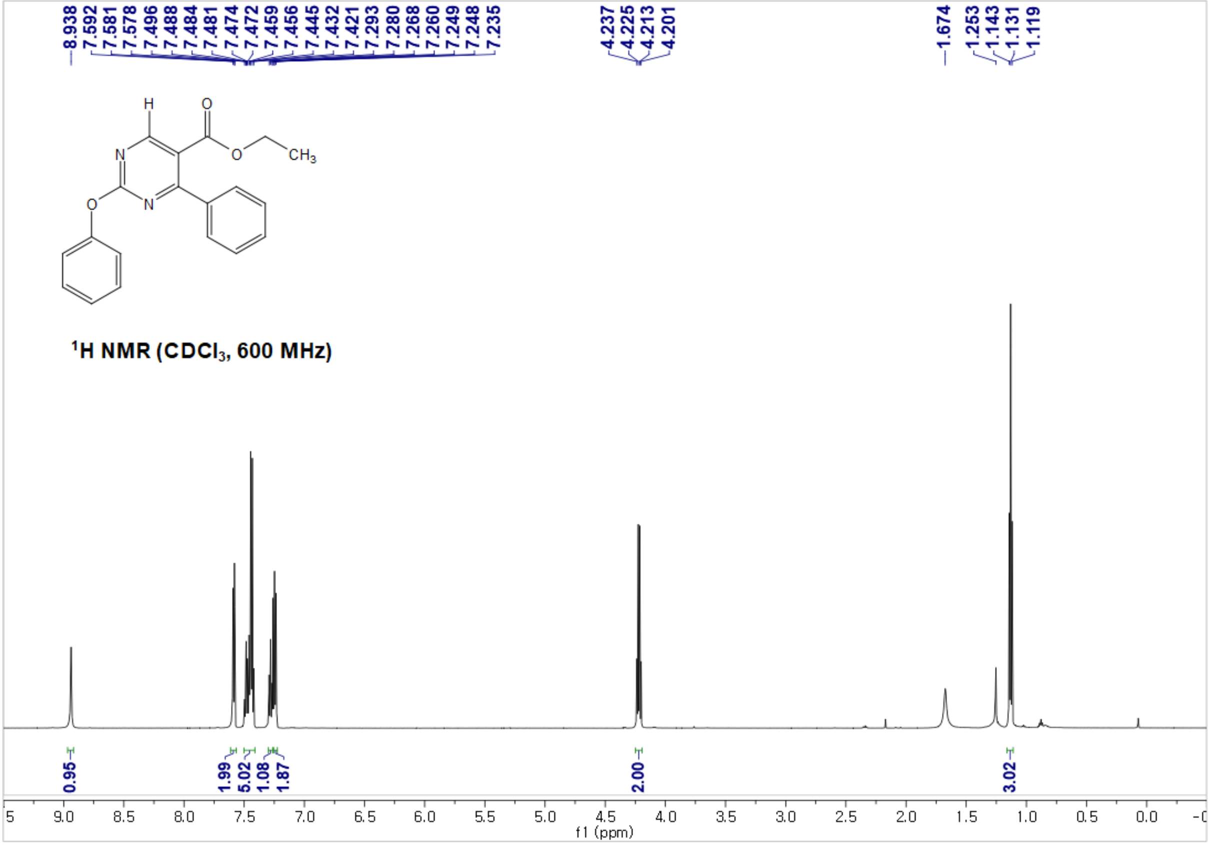

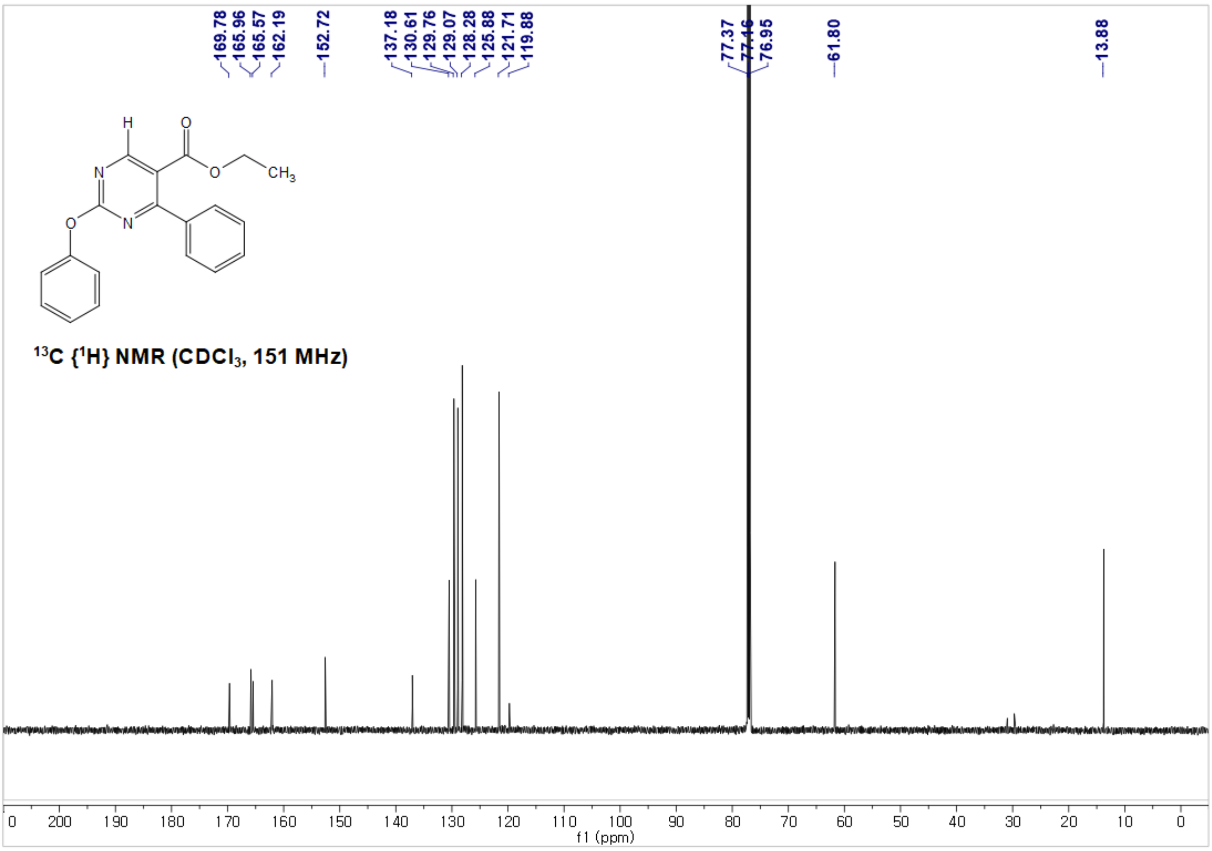

Ethyl 4-ethyl-2-phenoxy-6-phenylpyrimidine-5-carboxylate (**3n**).

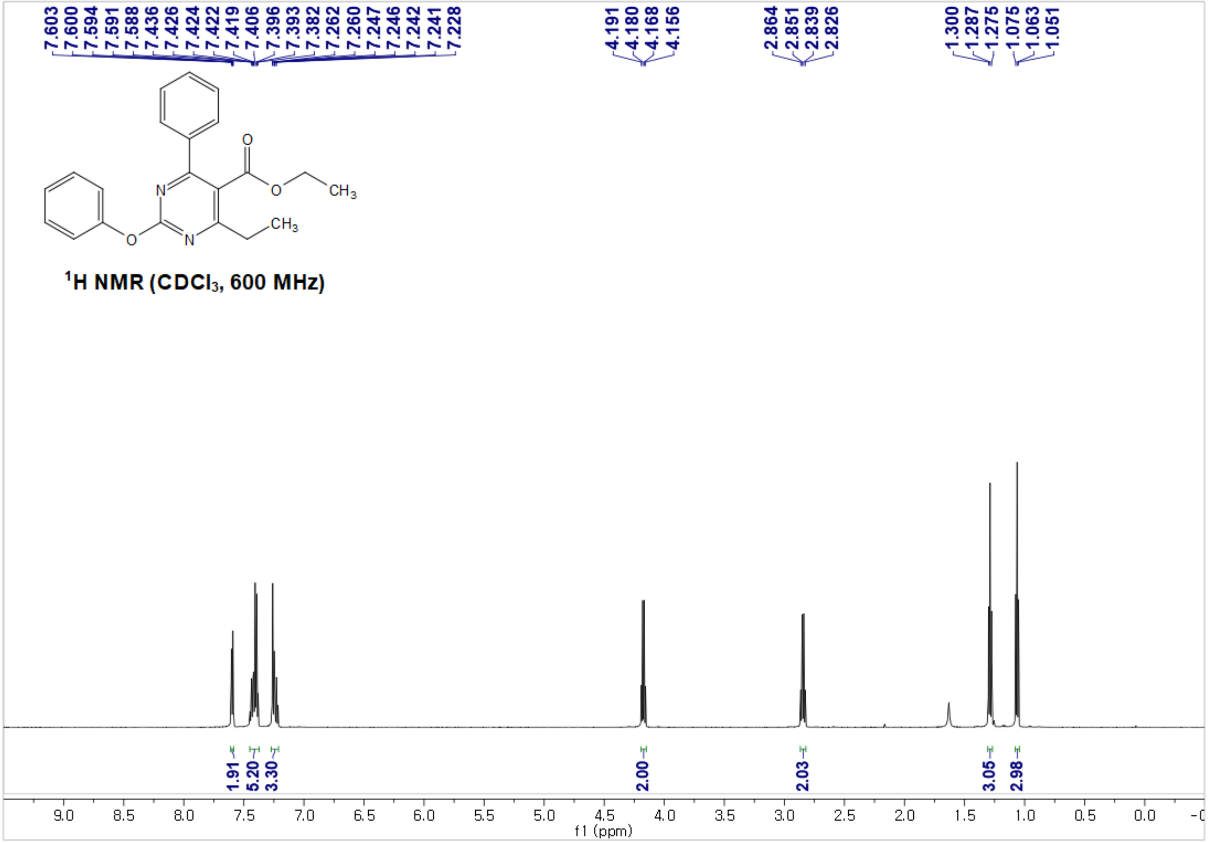

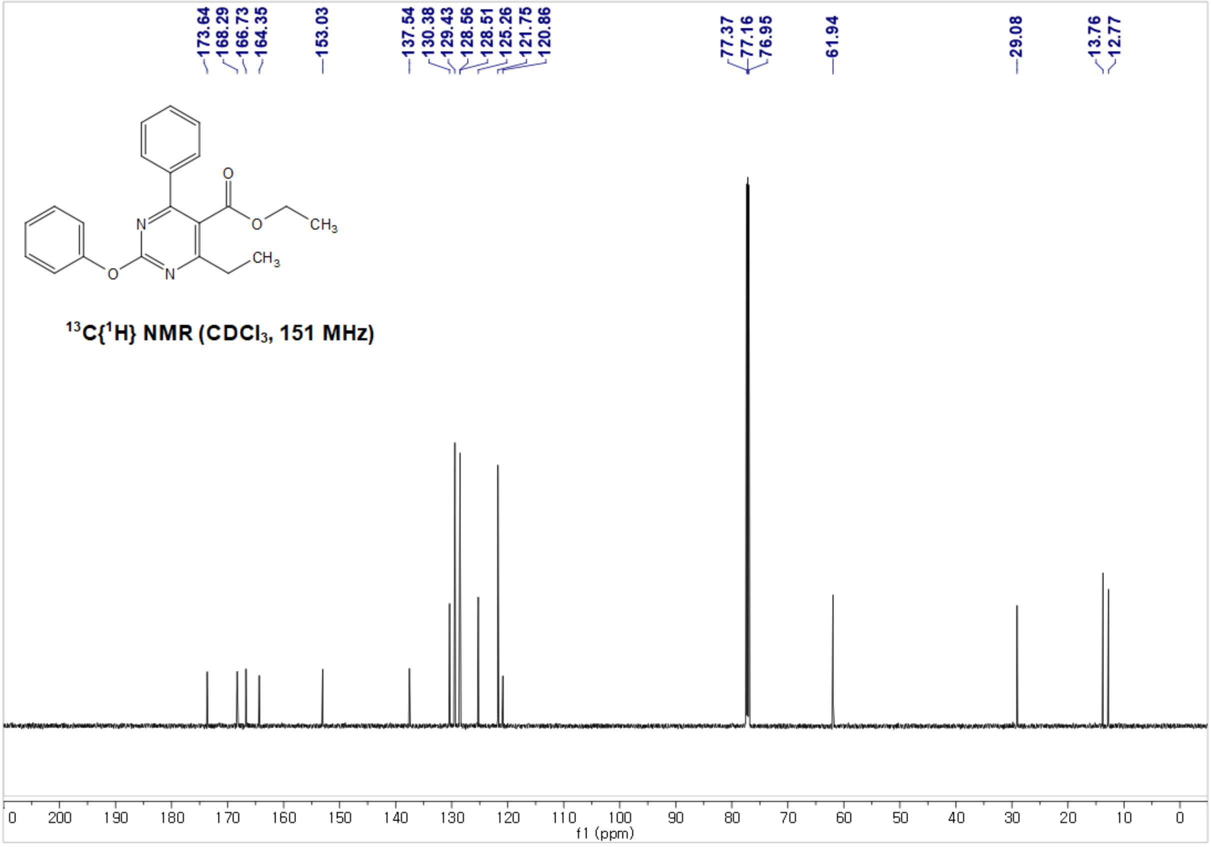

Ethyl 2-phenoxy-4,6-diphenylpyrimidine-5-carboxylate(**3o**).

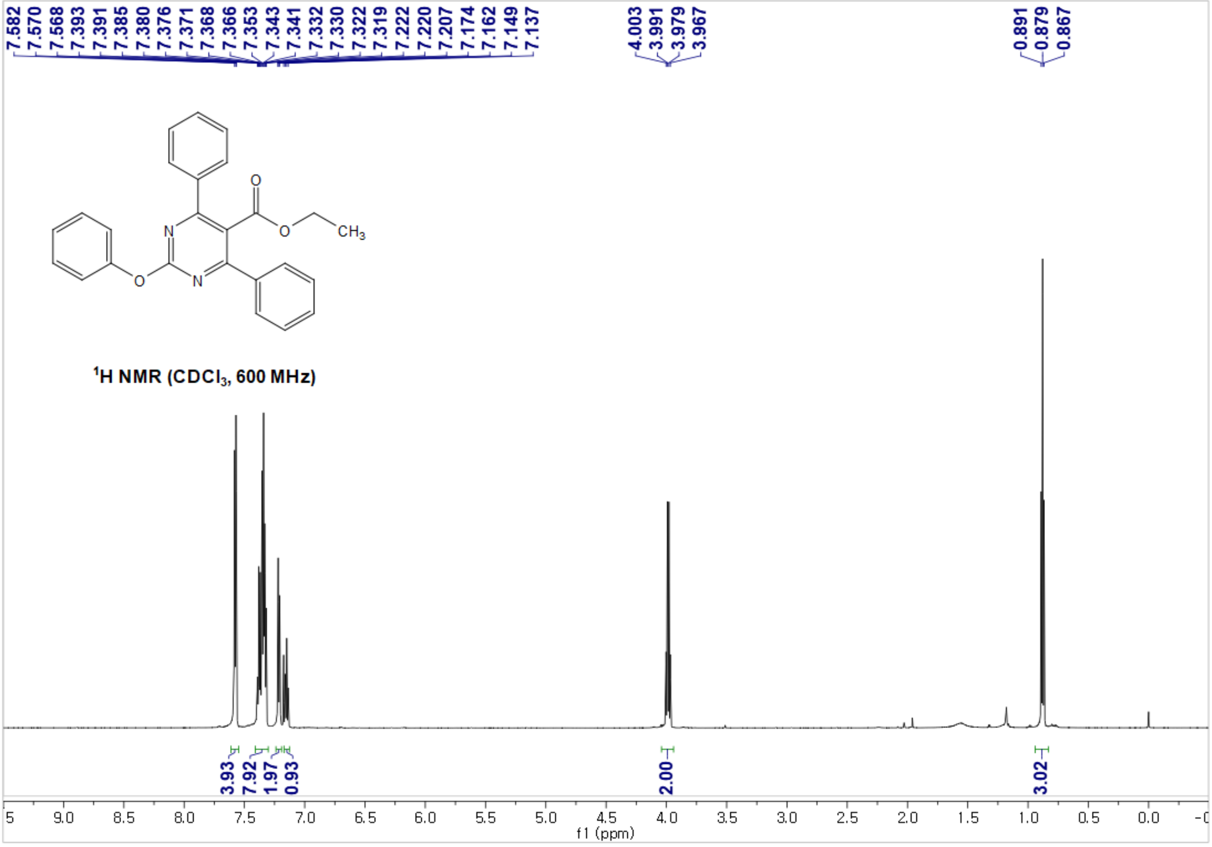

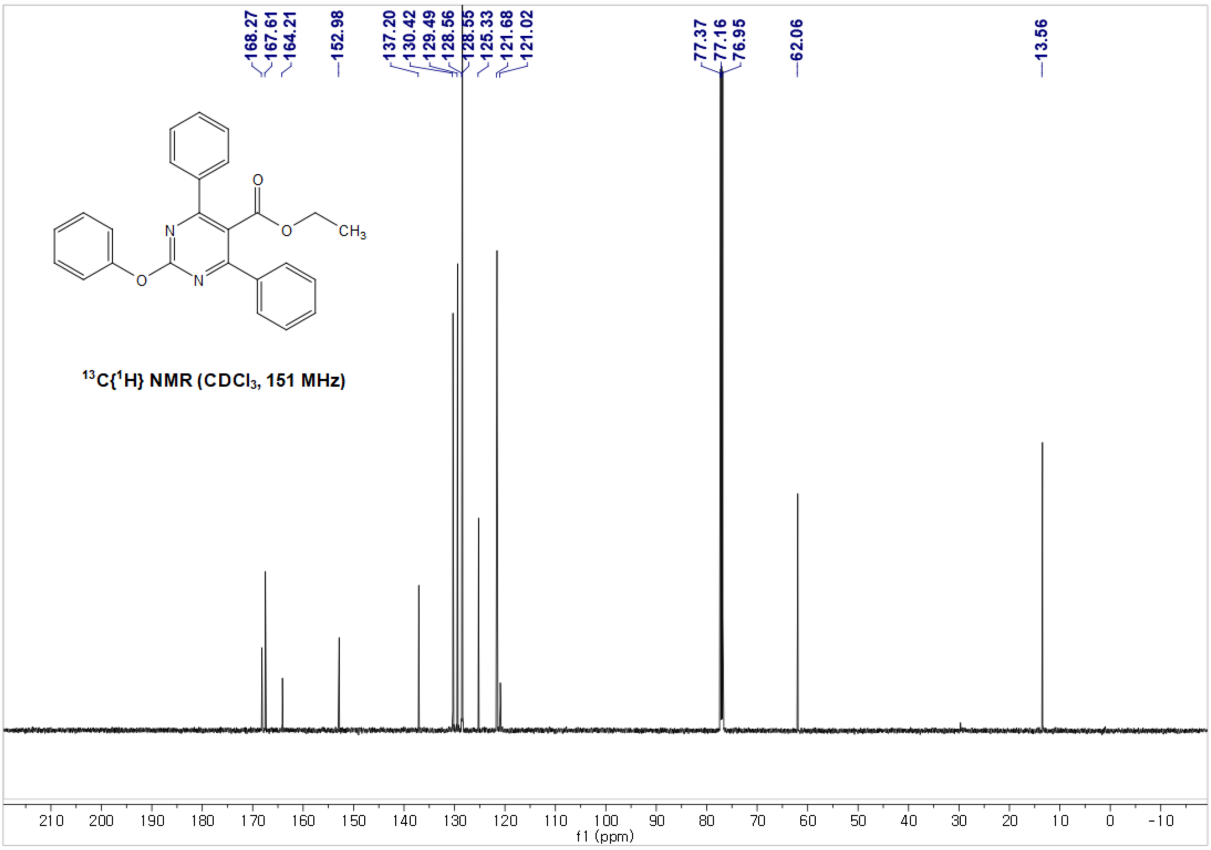

Ethyl 4-methyl-2-phenoxy-6-(p-tolyl)pyrimidine-5-carboxylate (**3p**).

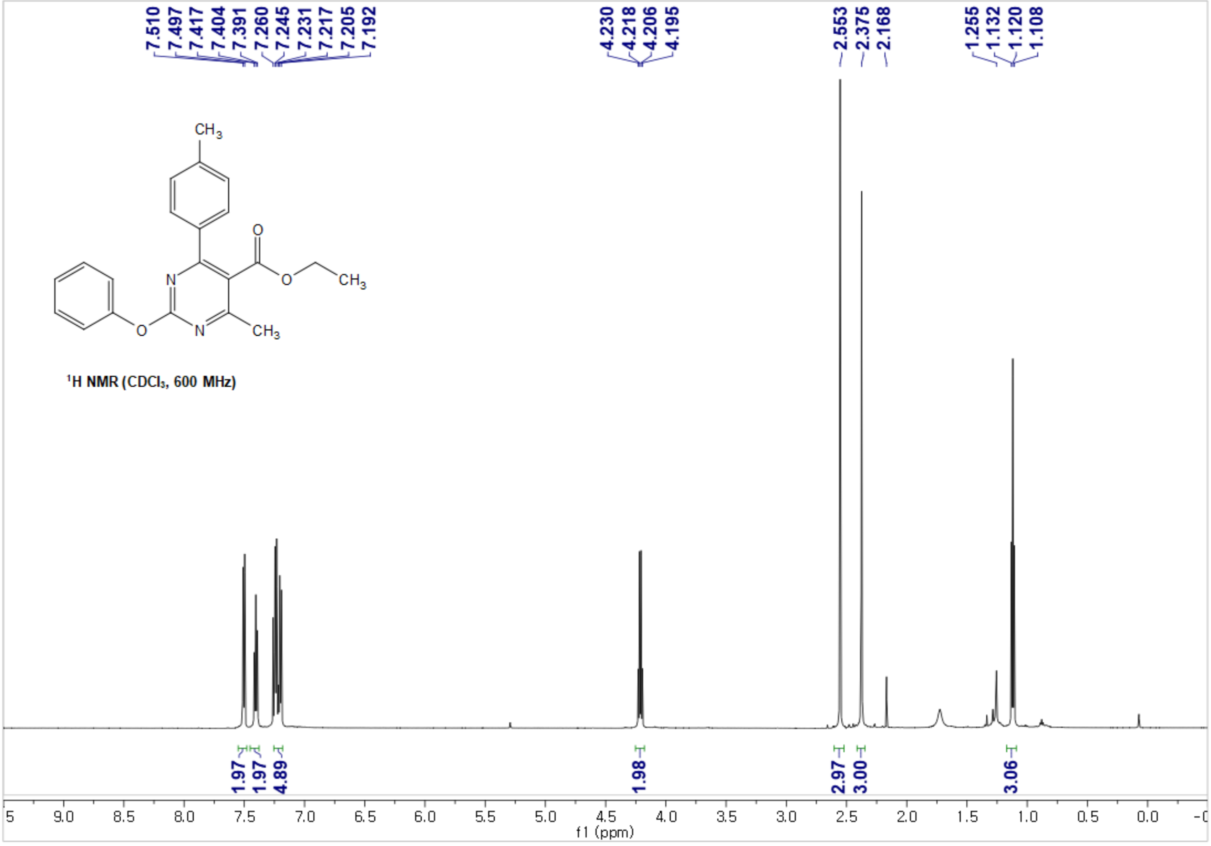

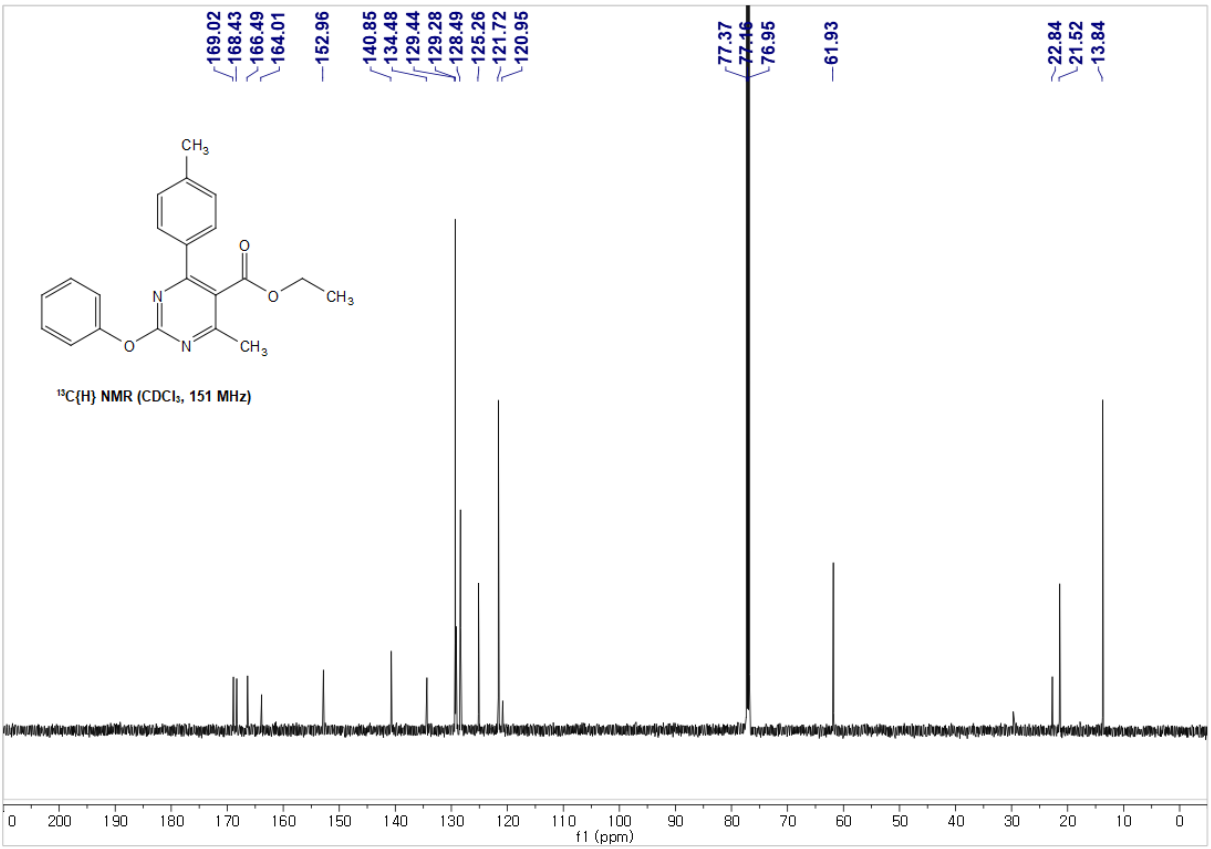

Ethyl 4-(4-methoxyphenyl)-2-phenoxy-6-phenylpyrimidine-5-carboxylate (**3q**).

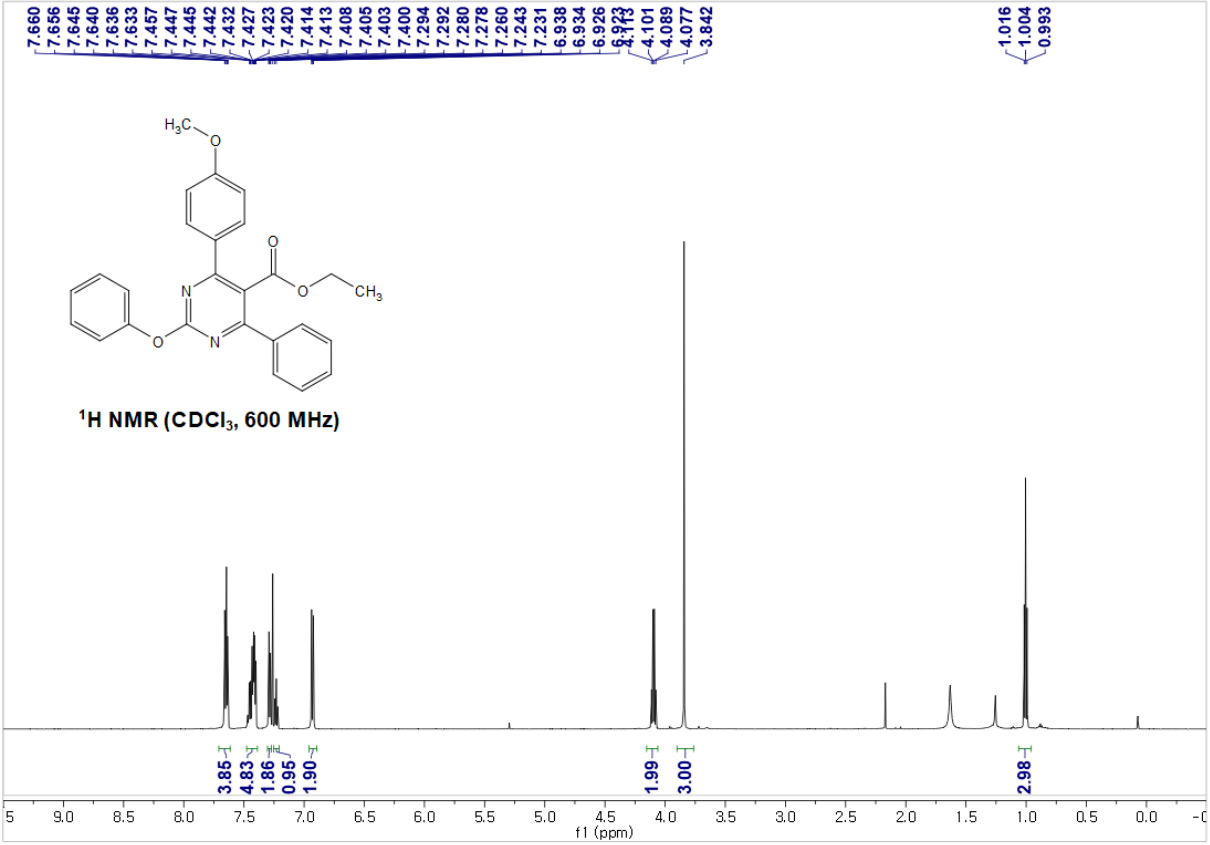

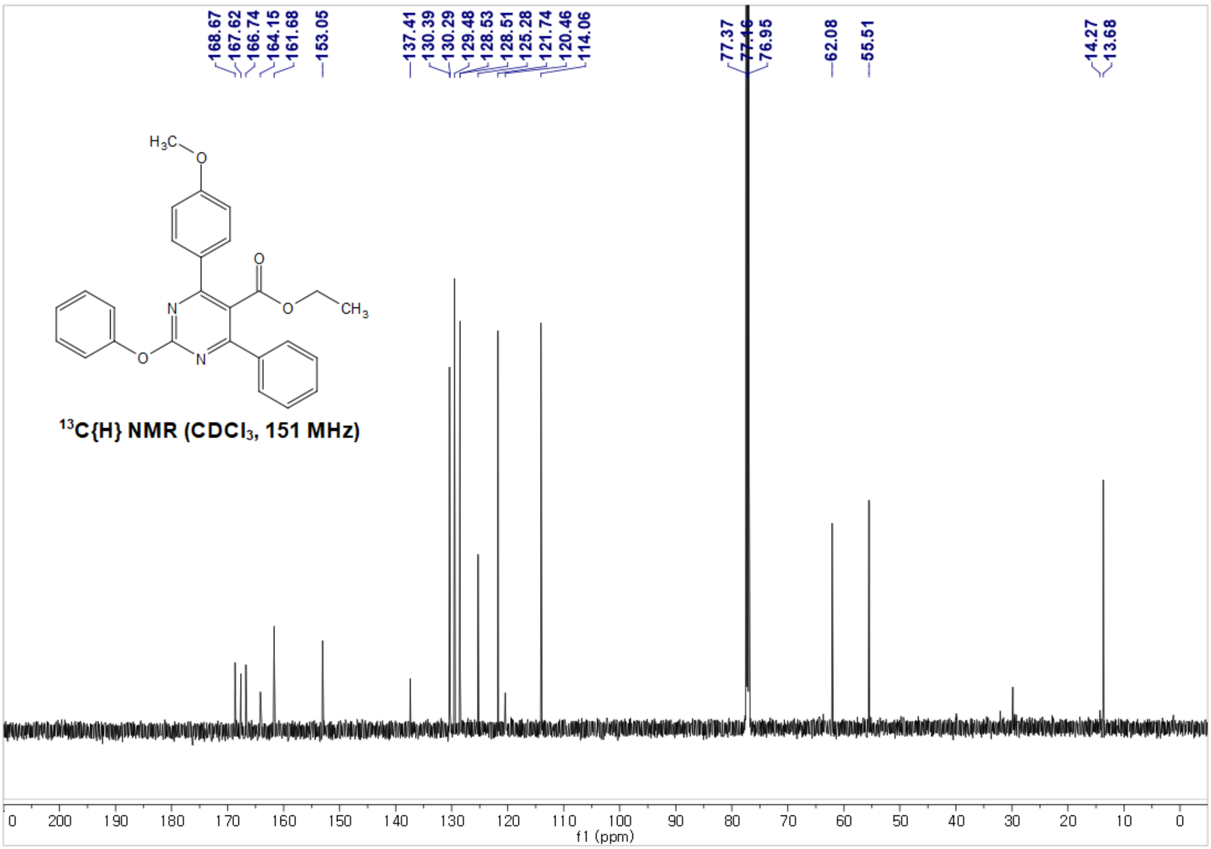

Ethyl 4-(4-fluorophenyl)-6-methyl-2-phenoxypyrimidine-5-carboxylate (**3r**)

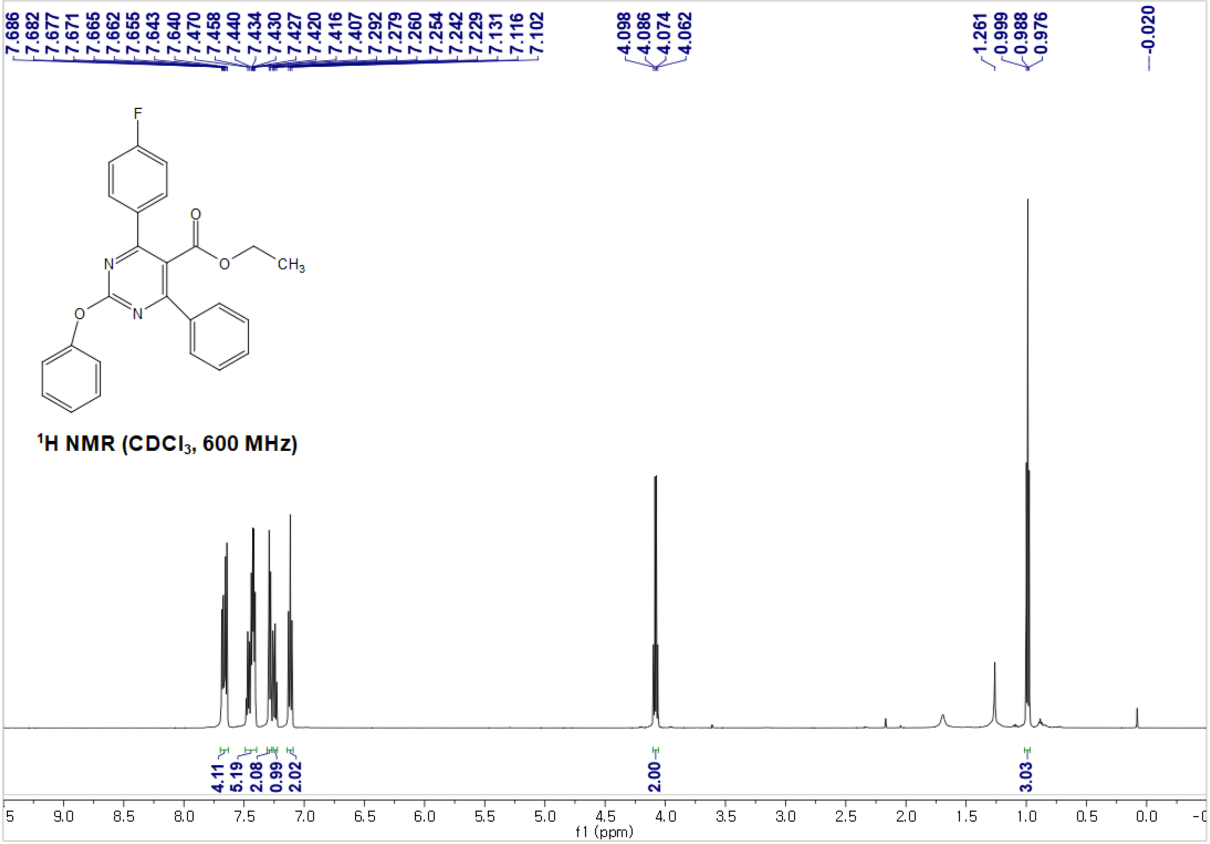

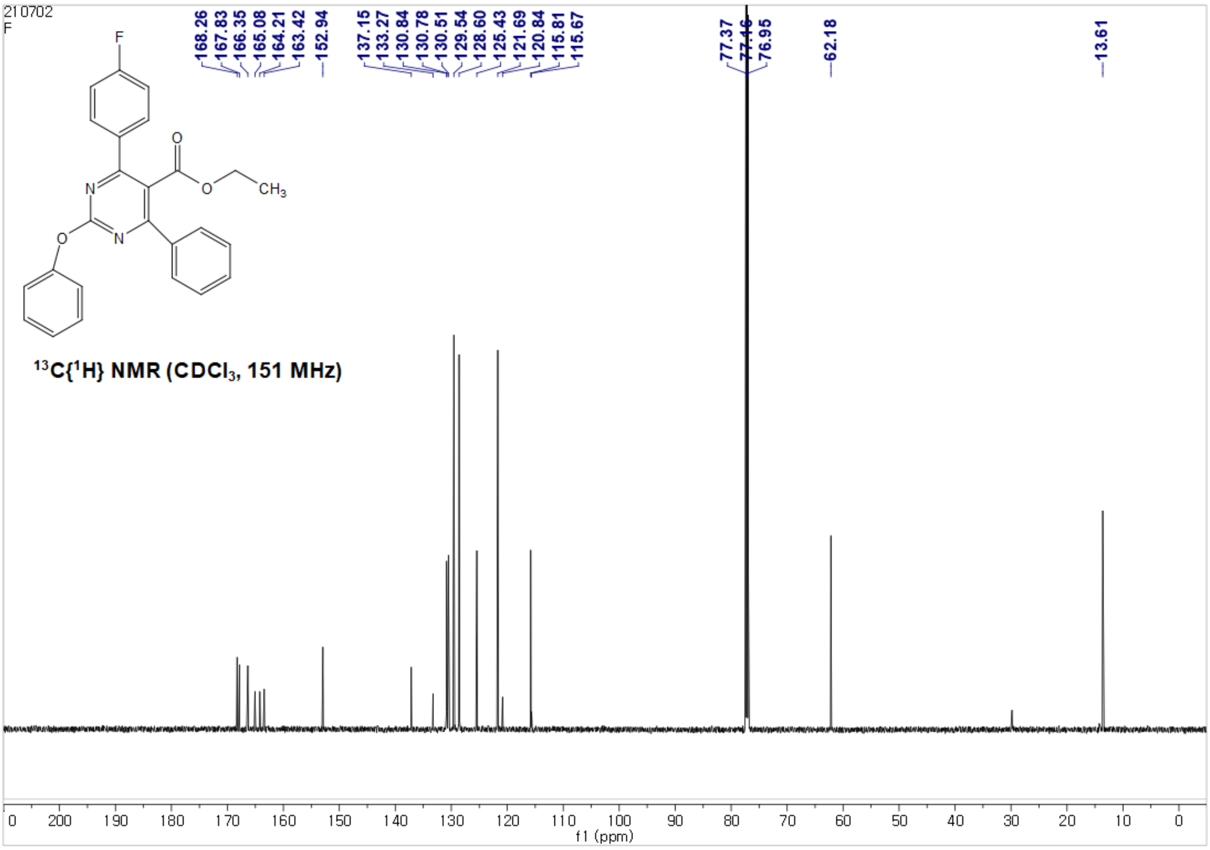

Ethyl 4-(4-chlorophenyl)-2-phenoxy-6-phenylpyrimidine-5-carboxylate (**3s**)

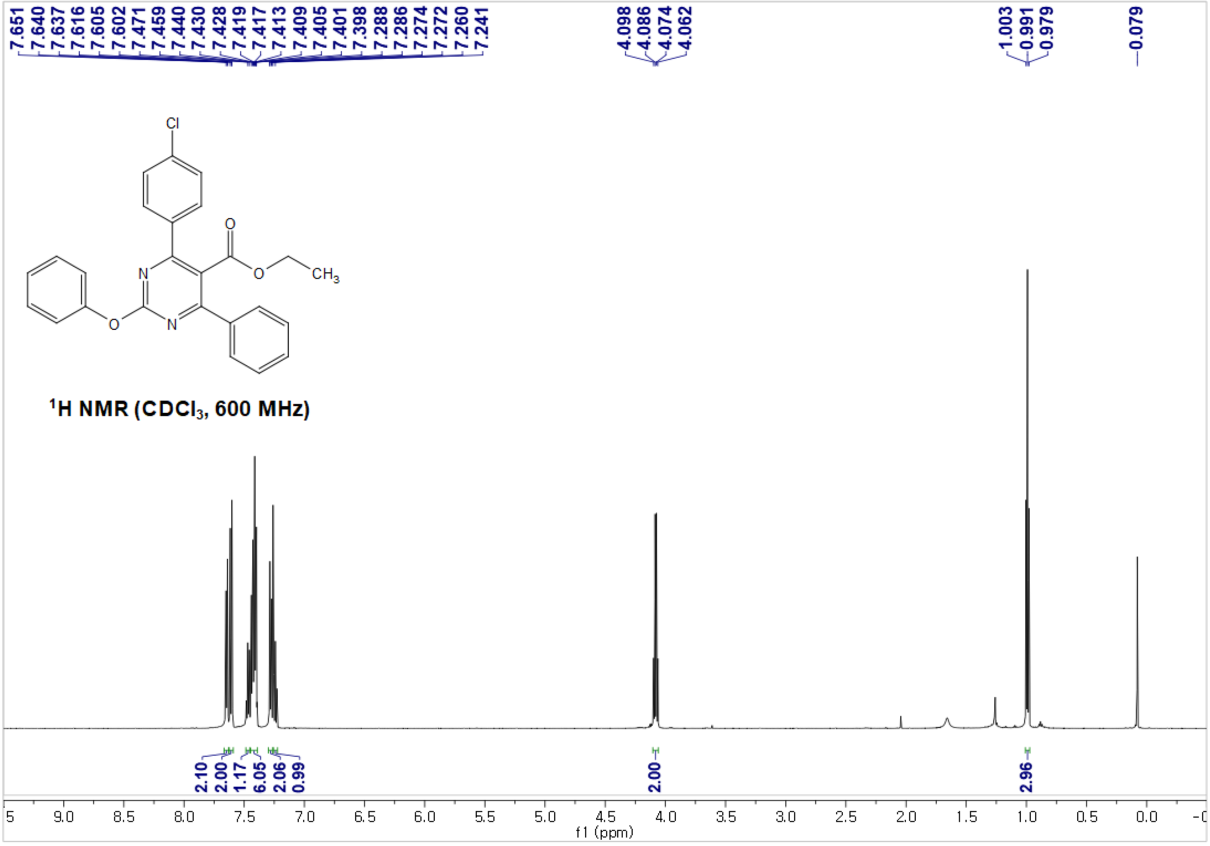

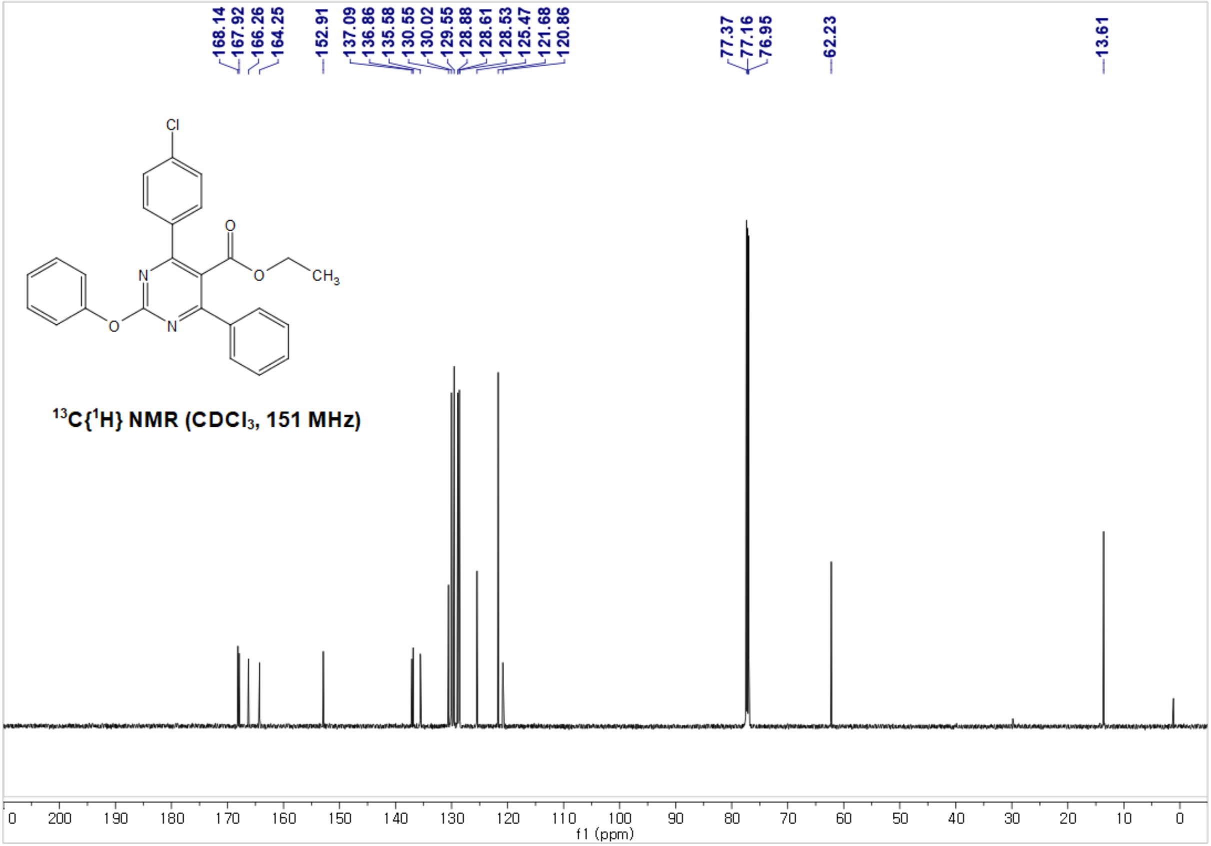

Ethyl 4-(4-bromophenyl)-2-phenoxy-6-phenylpyrimidine-5-carboxylate (**3t**)

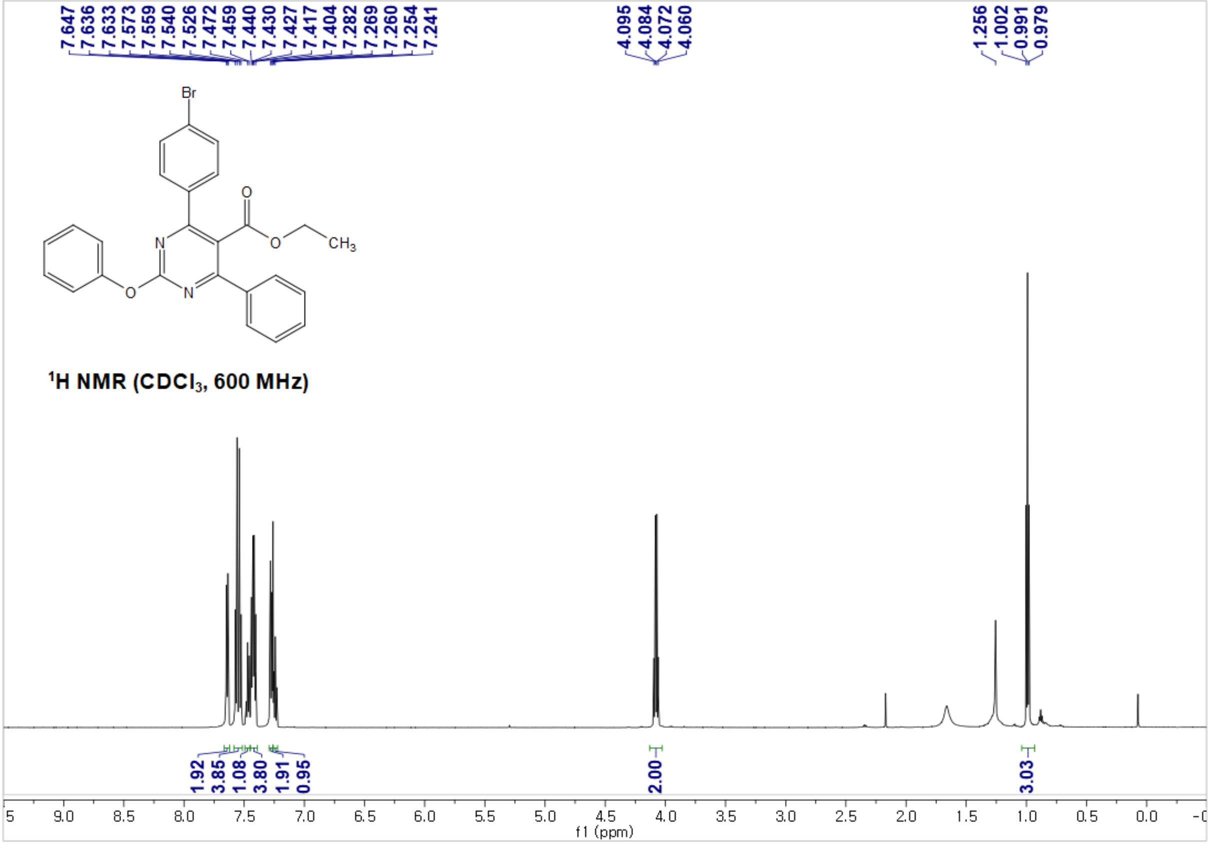

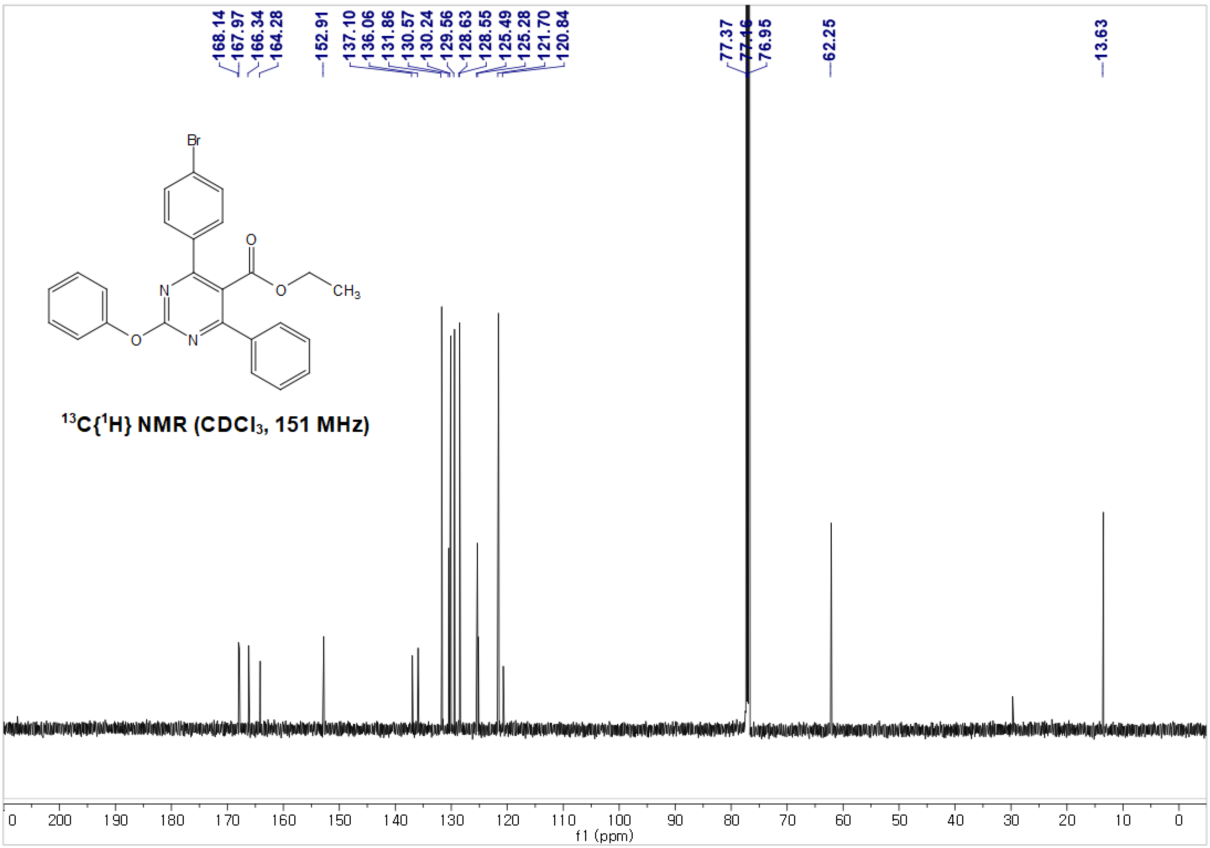

Ethyl 4-(4-nitrophenyl)-2-phenoxy-6-phenylpyrimidine-5-carboxylate (**3u**)

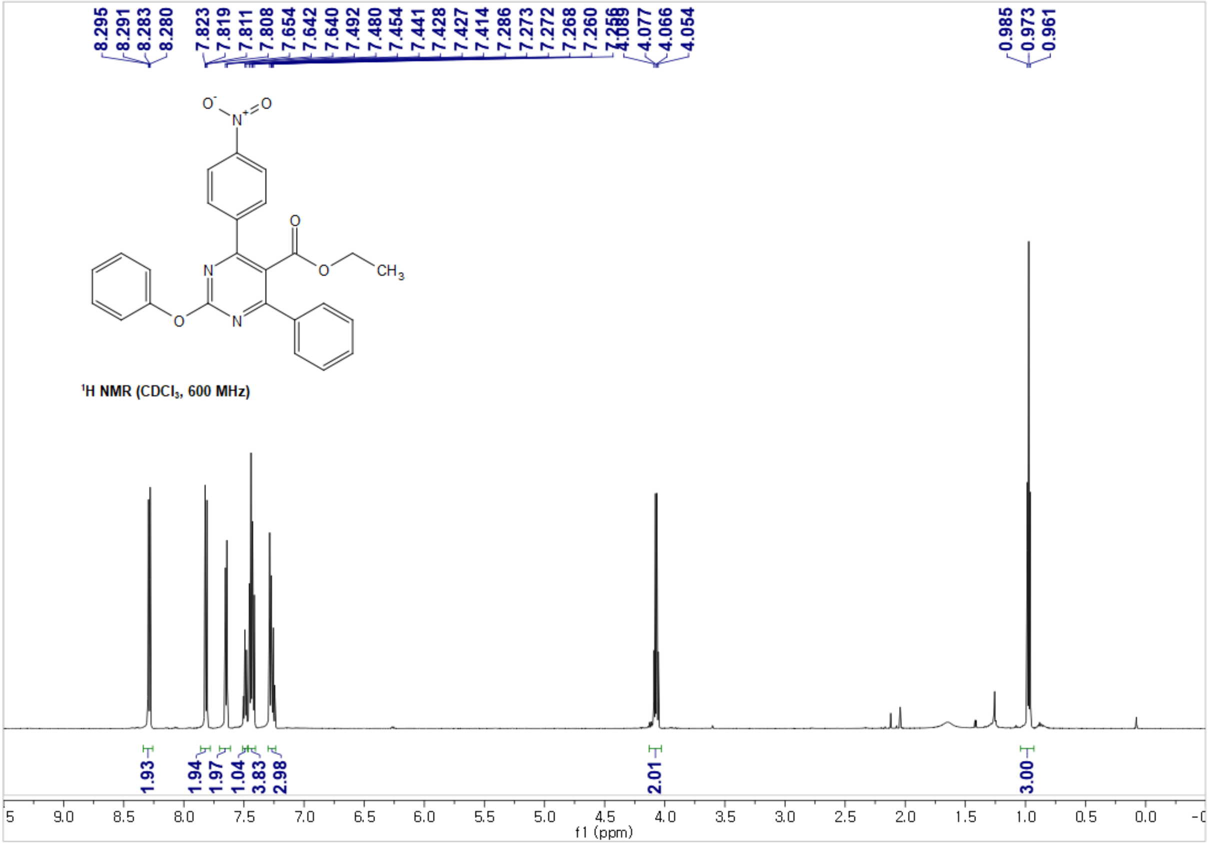

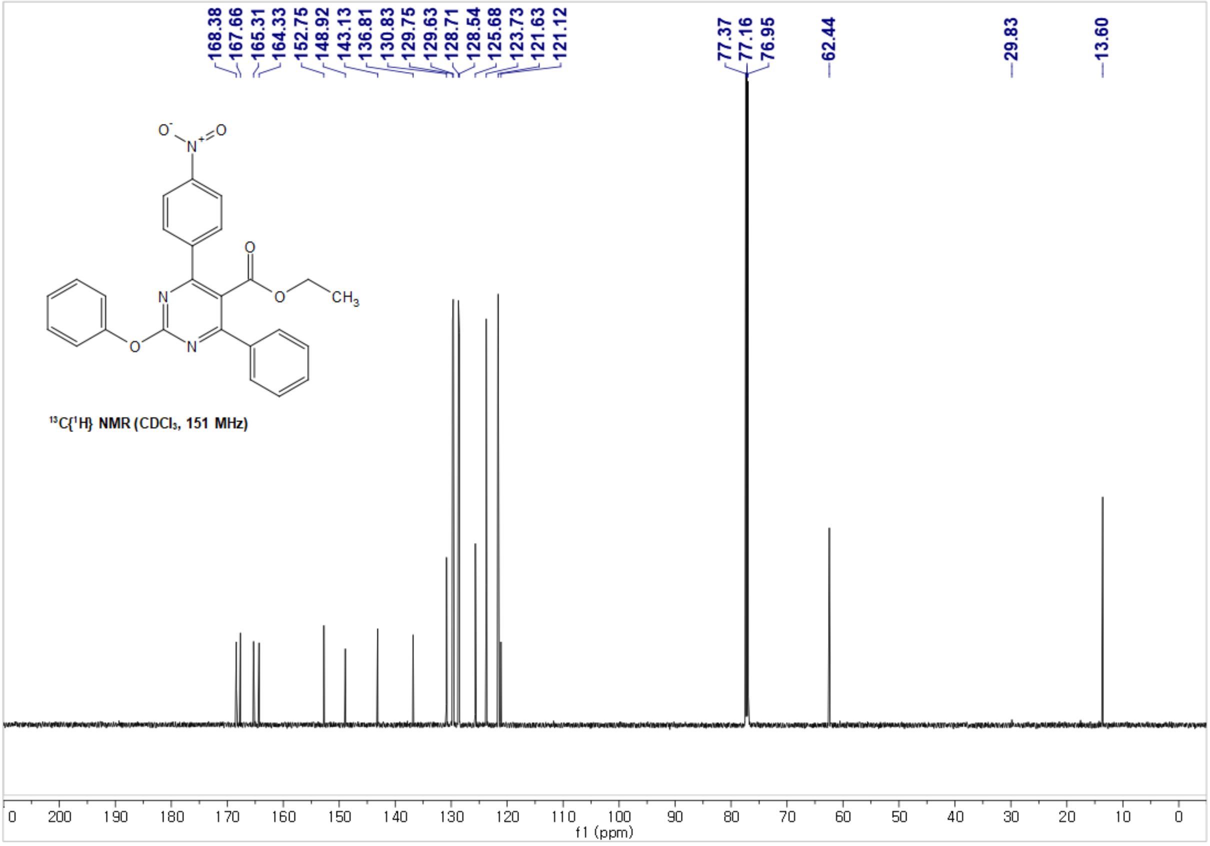

Ethyl 2-benzyl-4-(3,5-dimethylphenyl)-6-phenylpyrimidine-5-carboxylate (**3v**).

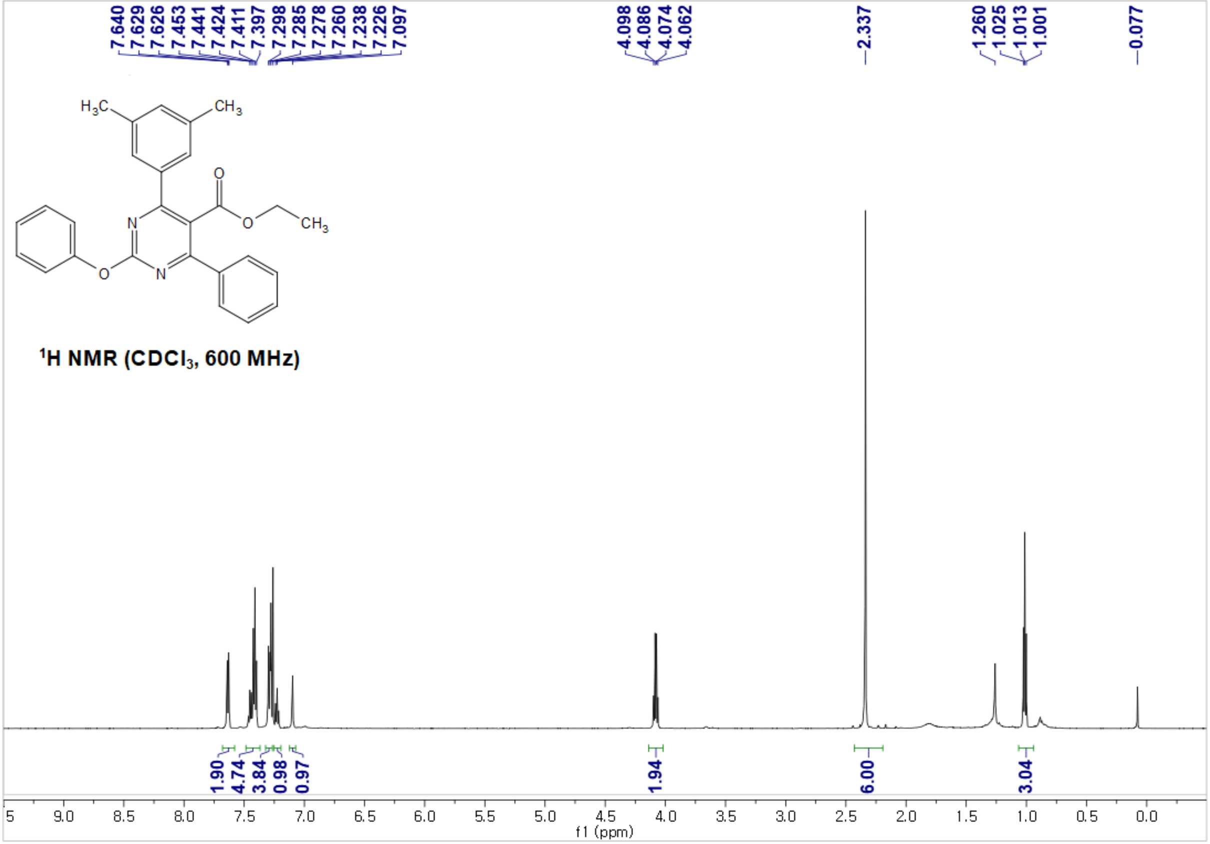

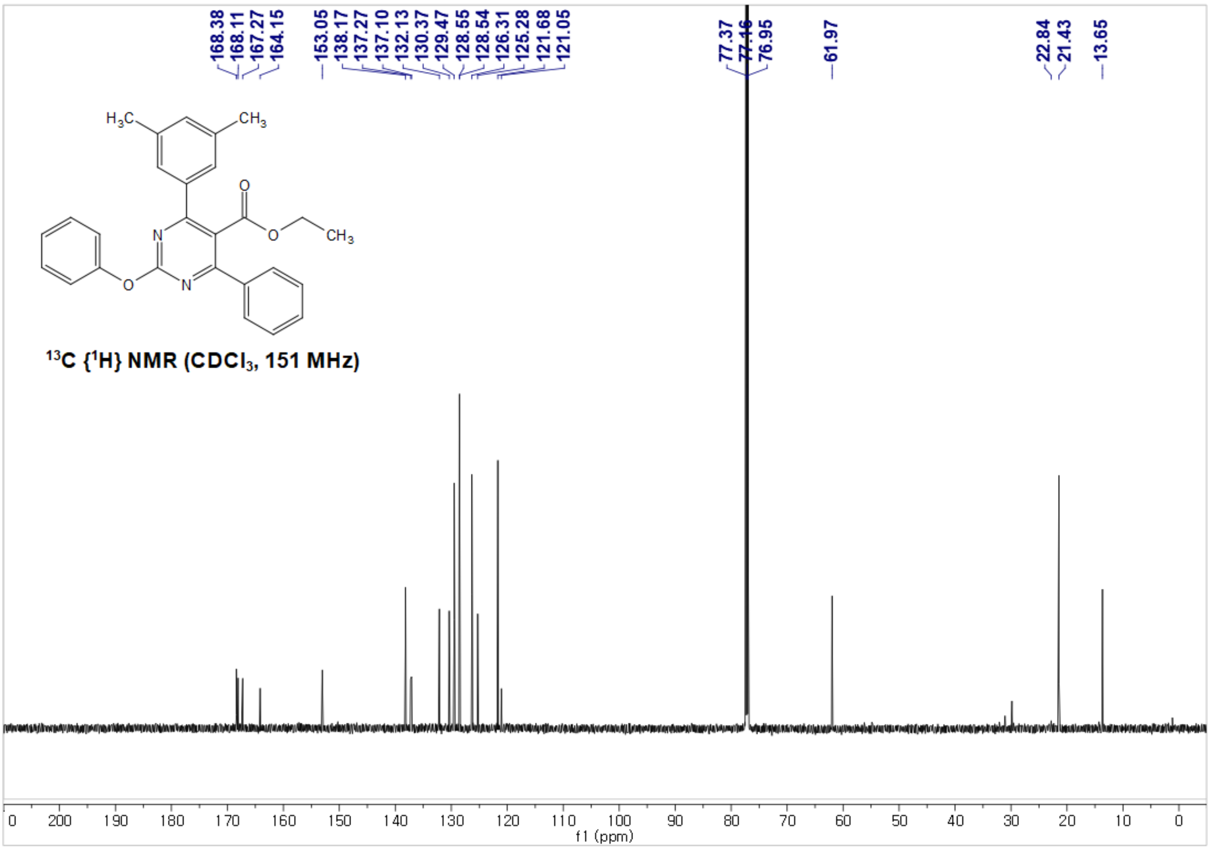

Ethyl (E)-4-methyl-2-phenoxy-6-(1-phenylprop-1-en-2-yl)pyrimidine-5-carboxylate (**3w**)

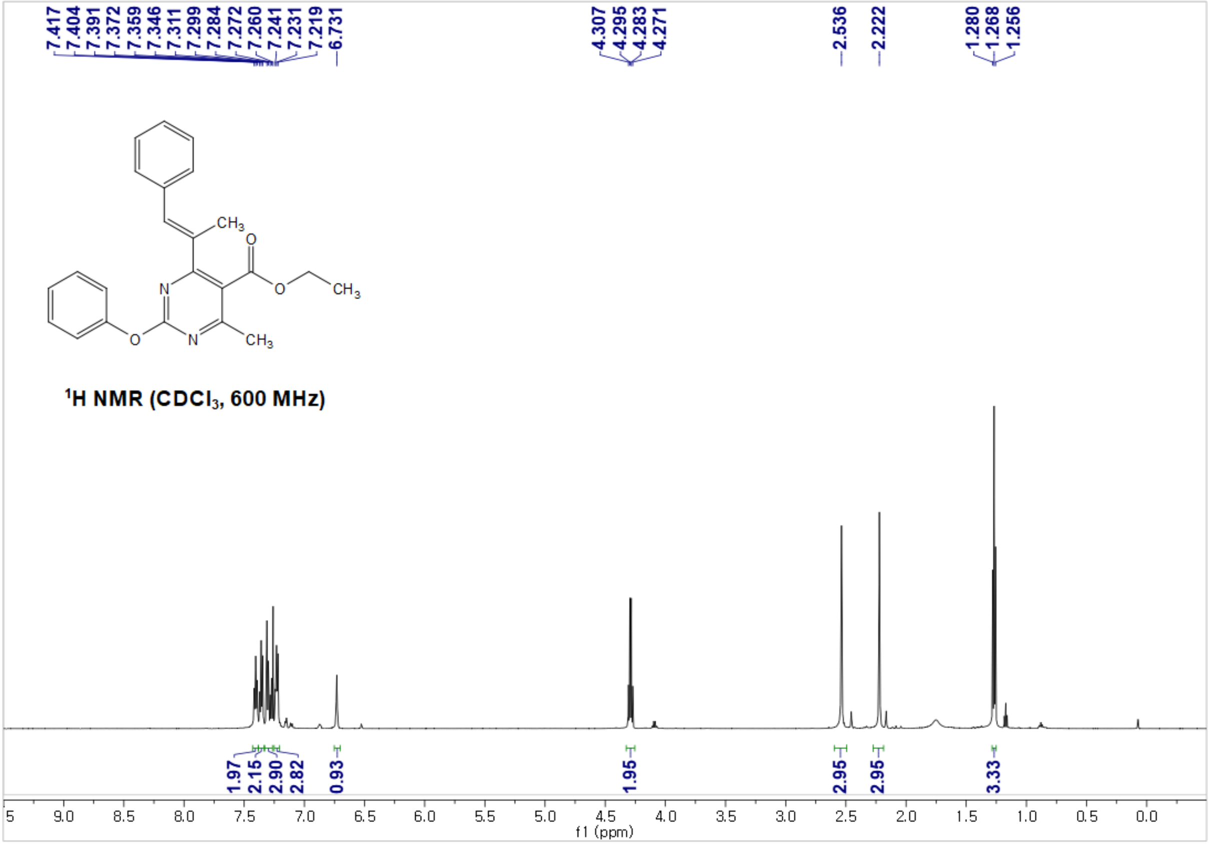

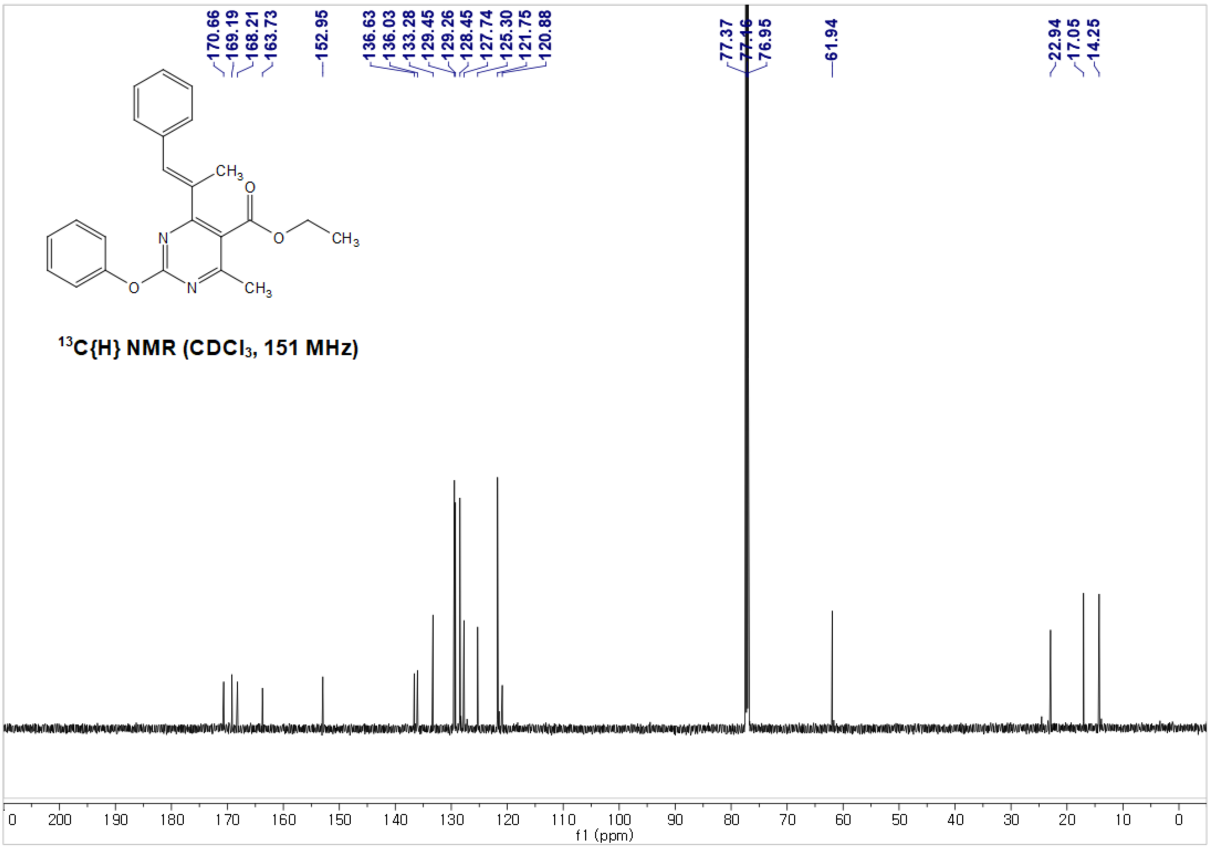

Ethyl 2-phenoxy-4-phenyl-6-(thiophen-2-yl)pyrimidine-5-carboxylate (**3x**)

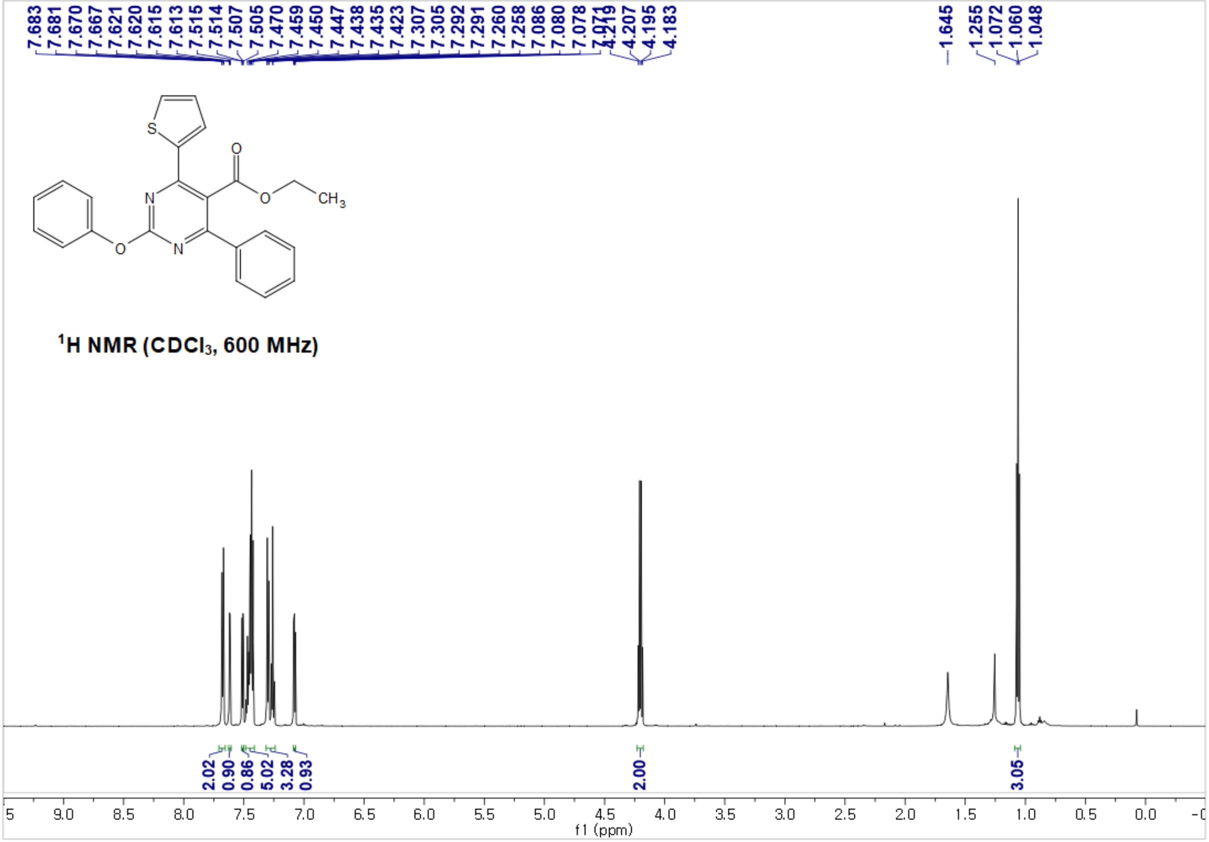

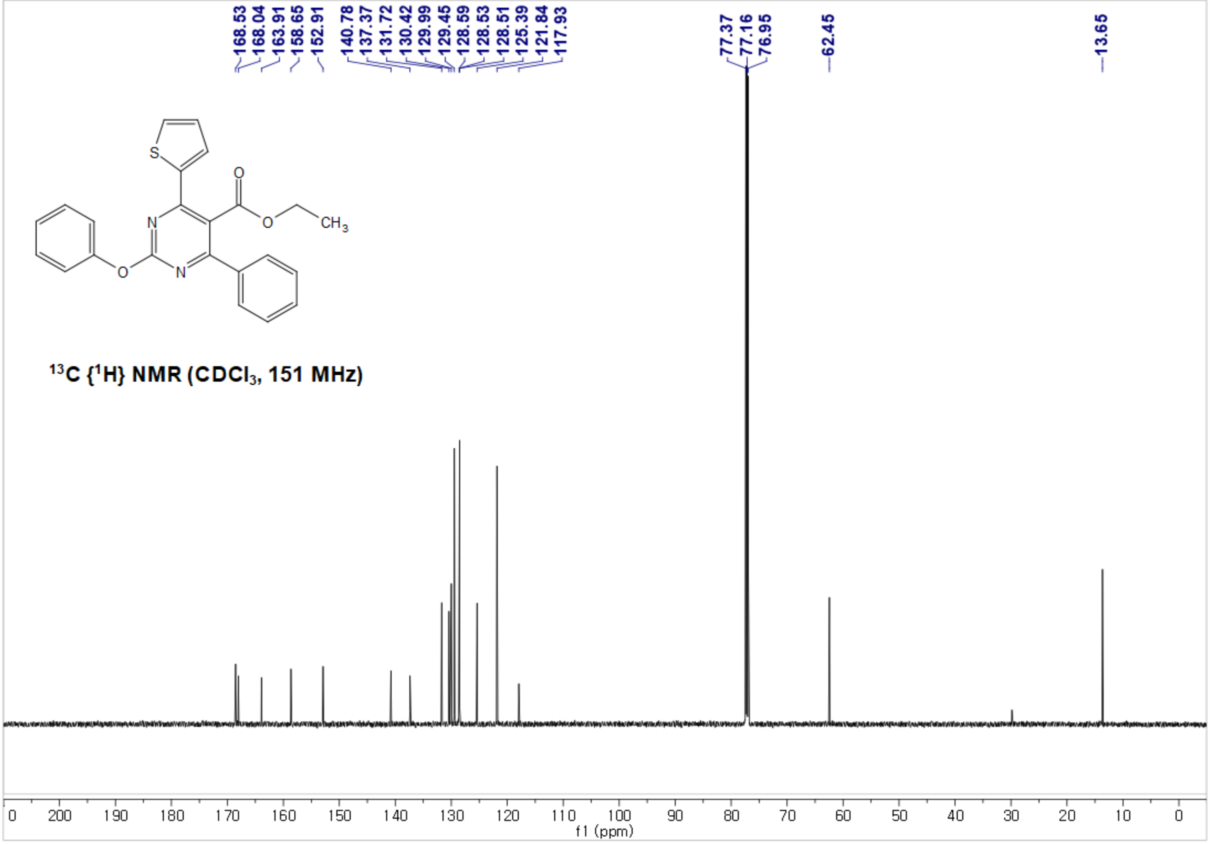

Ethyl 2-phenoxy-4-phenyl-6-(pyridin-3-yl)pyrimidine-5-carboxylate (**3y**)

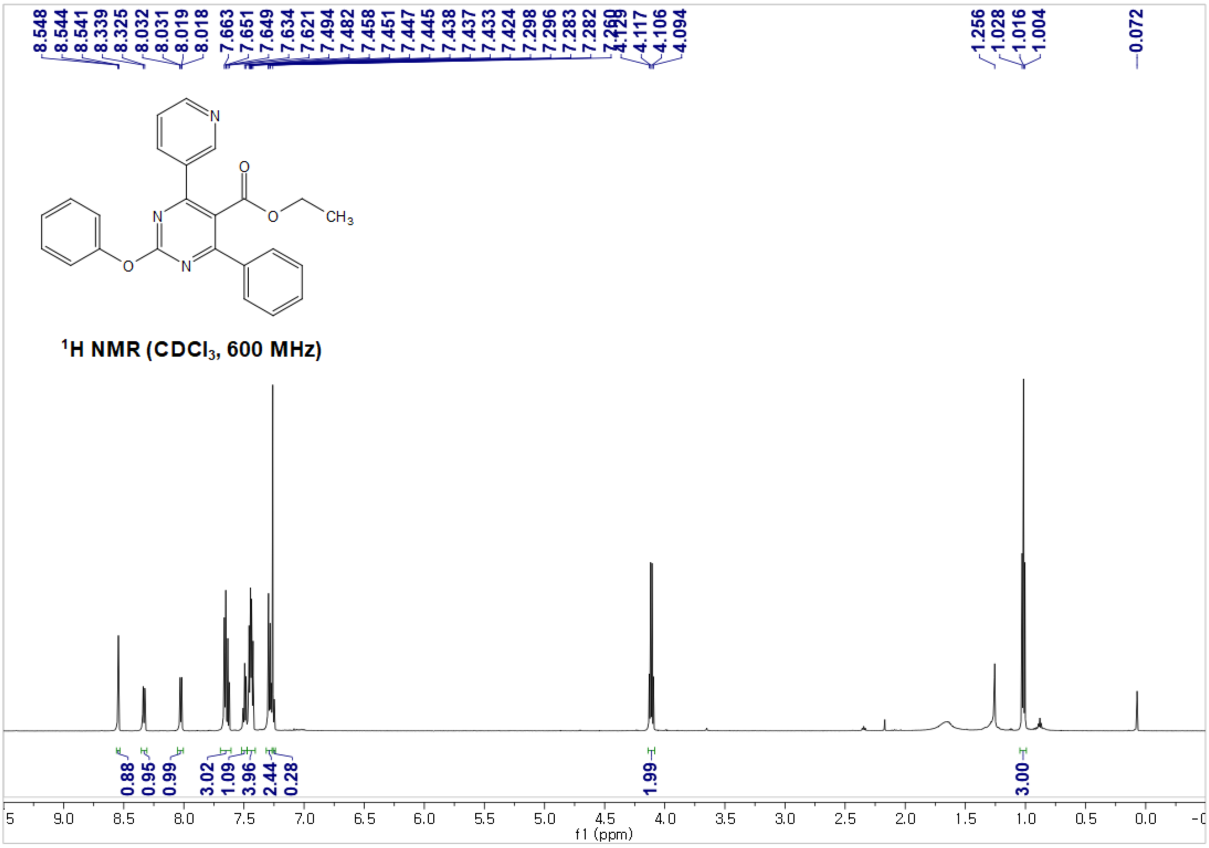

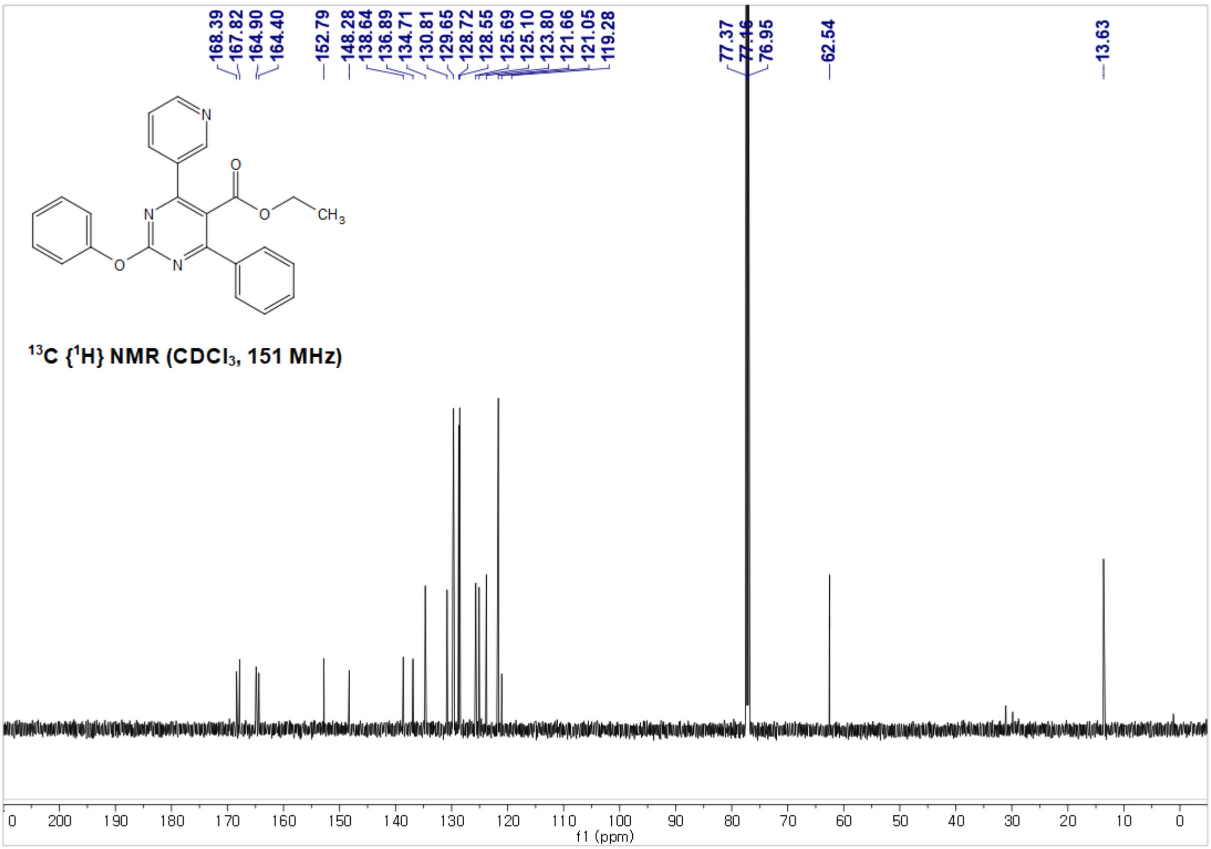

Ethyl 4-(naphthalen-1-yl)-2-phenoxy-6-phenylpyrimidine-5-carboxylate (**3z**)

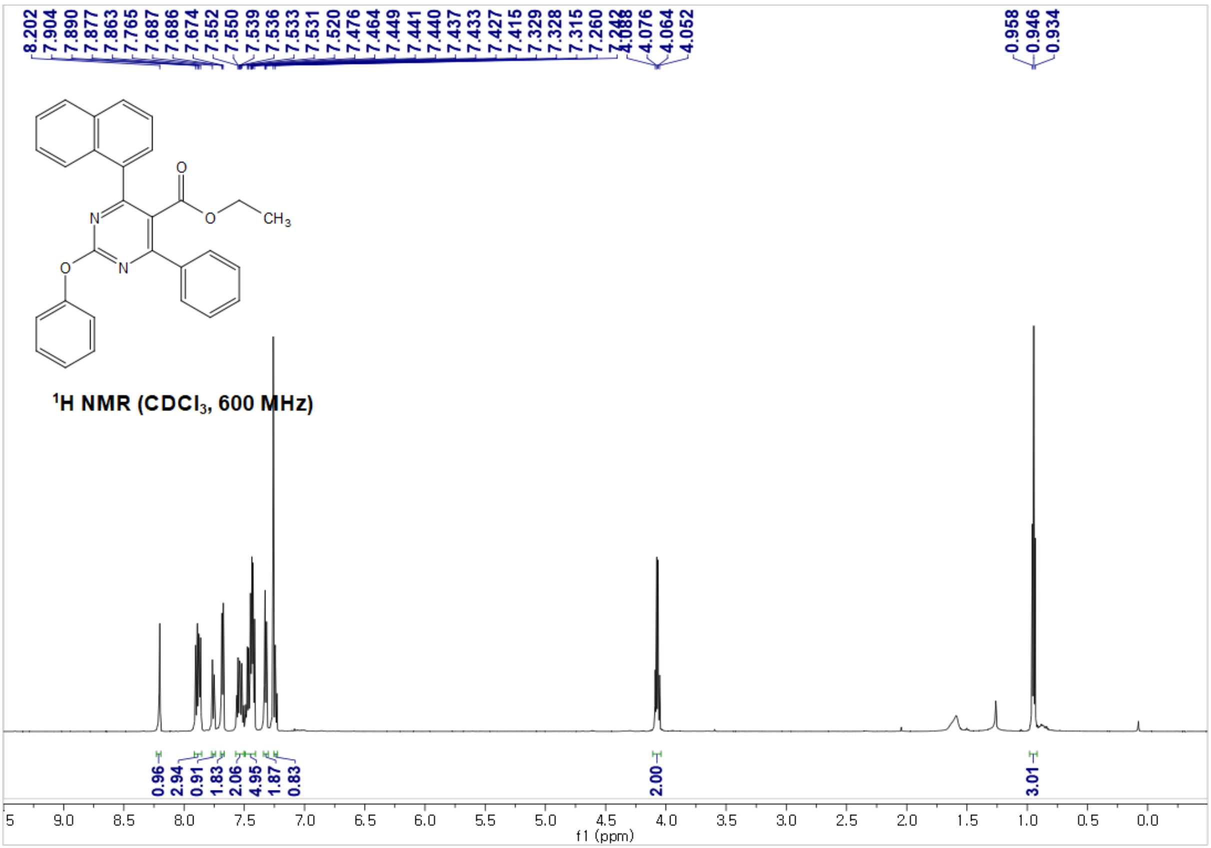

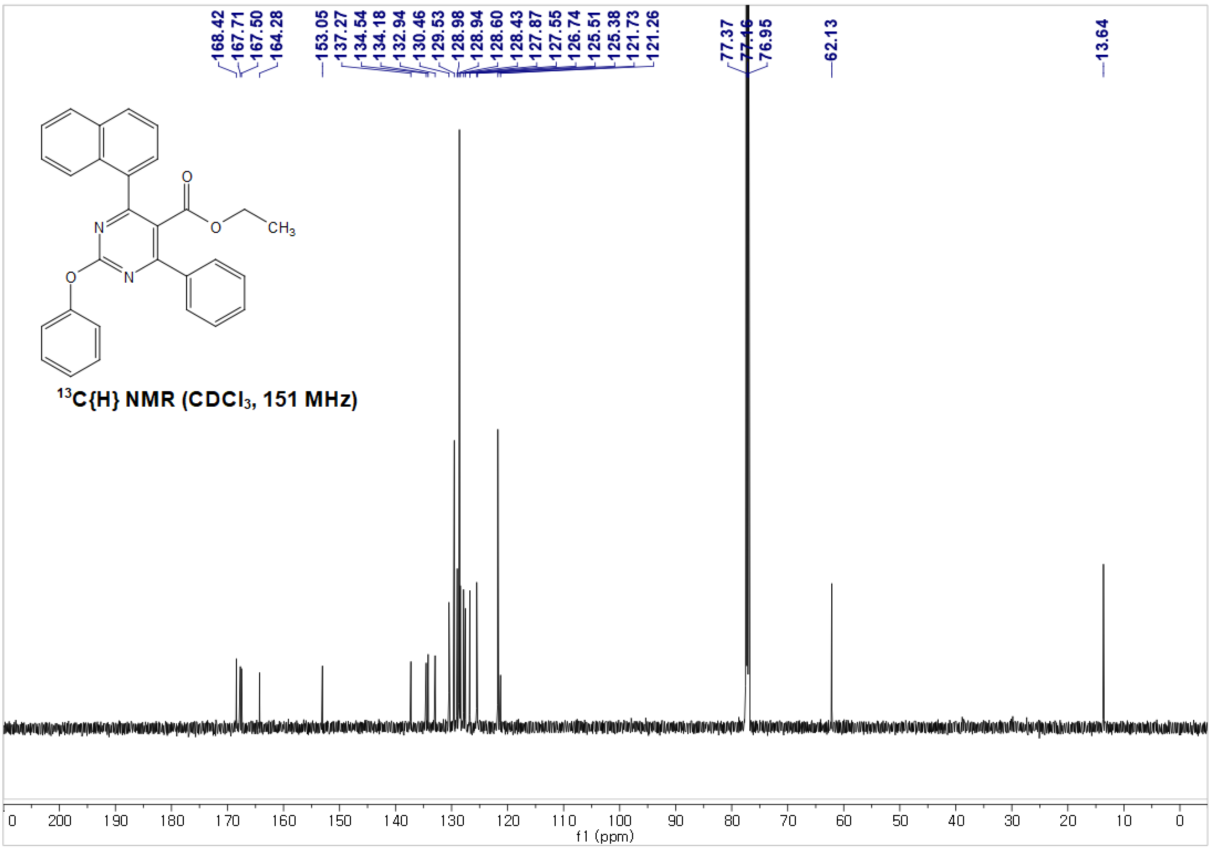

Ethyl 2-methoxy-4-methyl-6-phenylpyrimidine-5-carboxylate (**4a**)

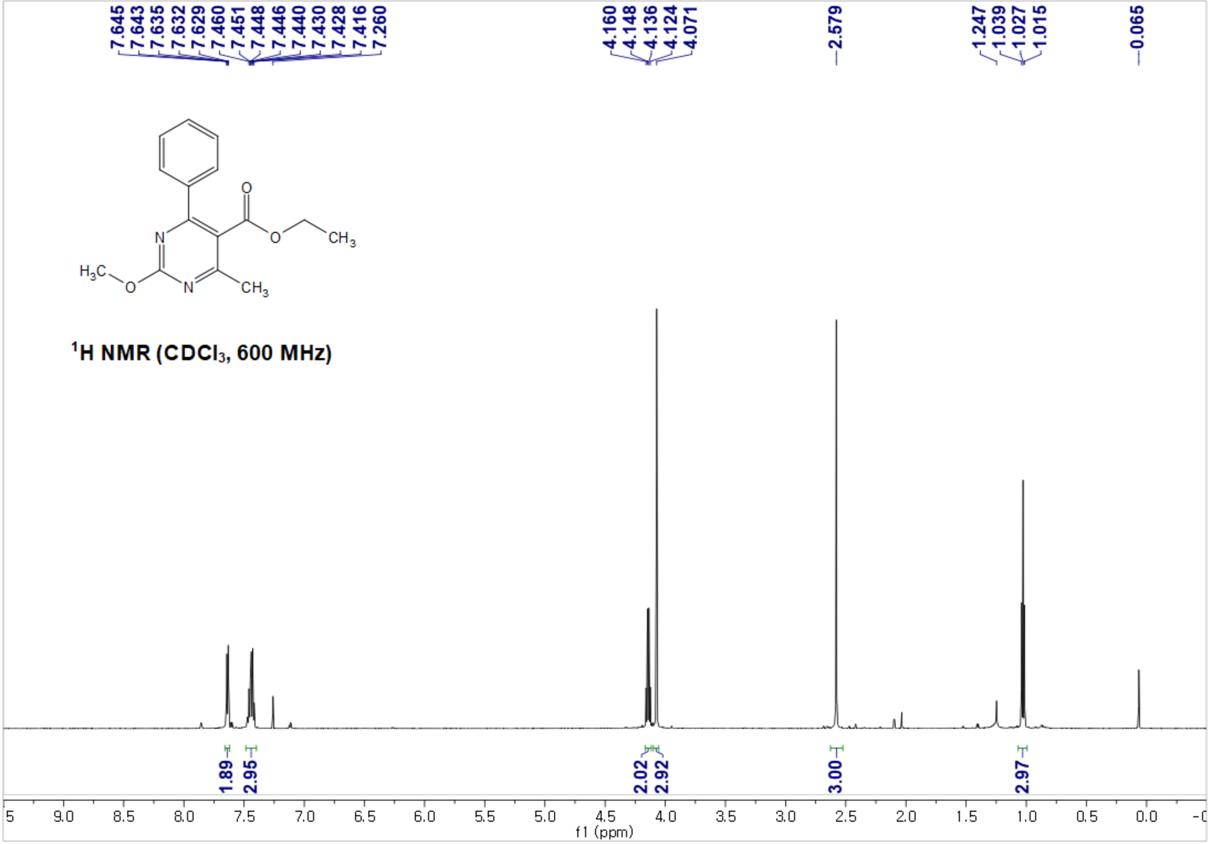

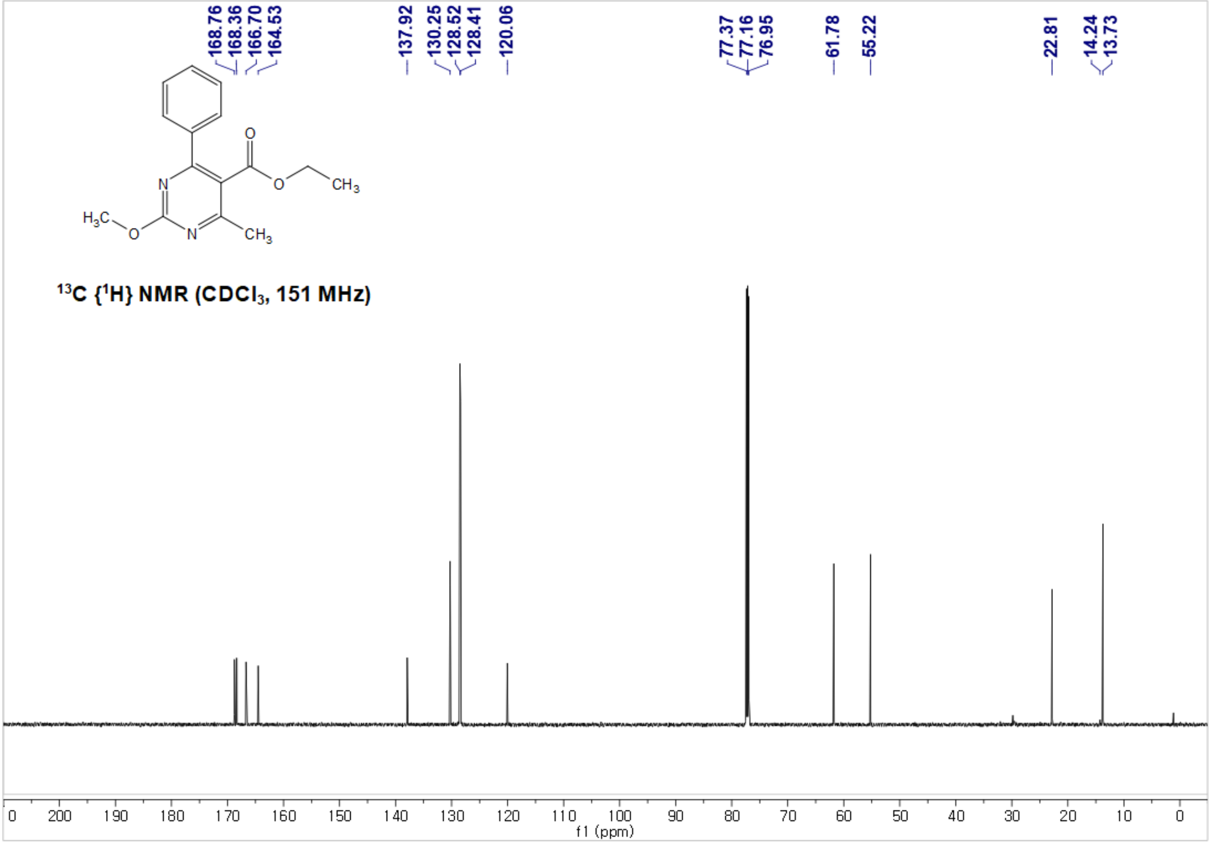

Ethyl 2-ethoxy-4-methyl-6-phenylpyrimidine-5-carboxylate (**4b**)

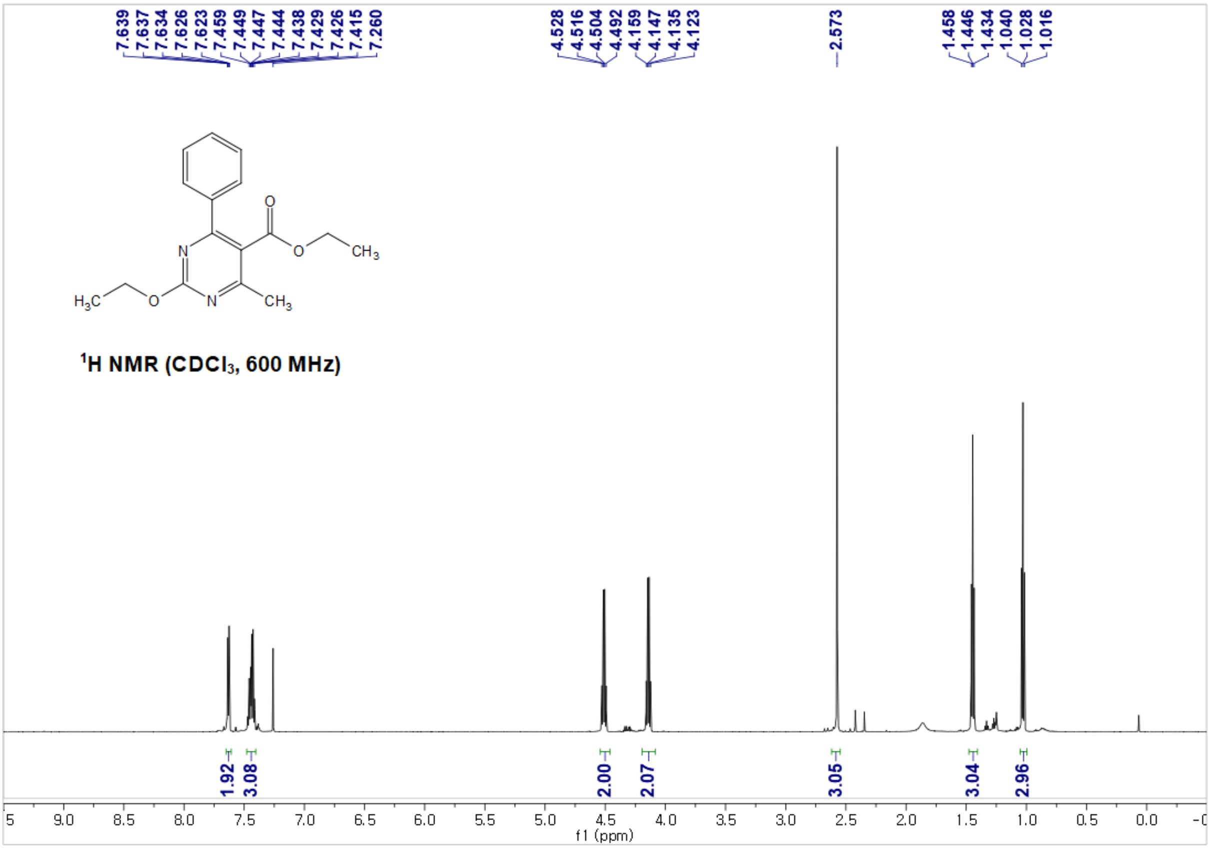

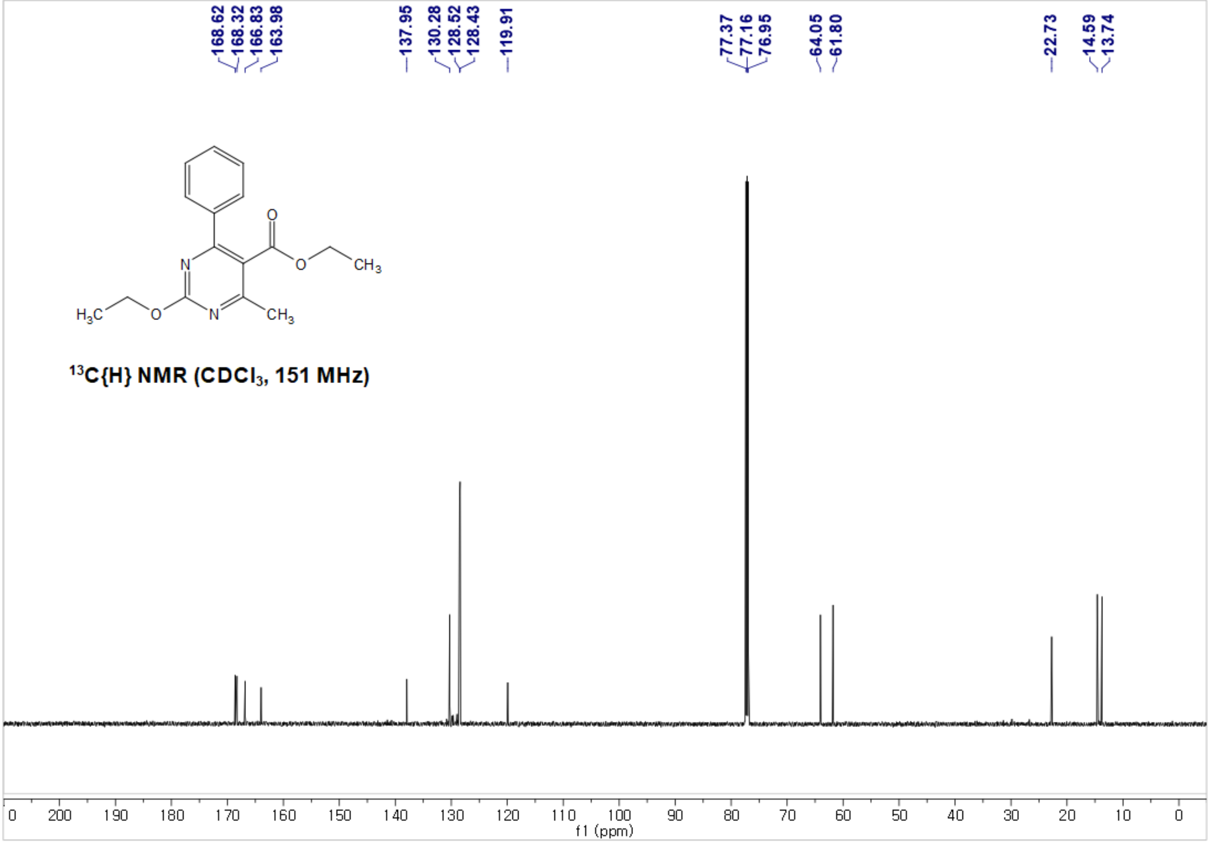

Ethyl 4-methyl-6-phenyl-2-propoxypyrimidine-5-carboxylate (**4c**)

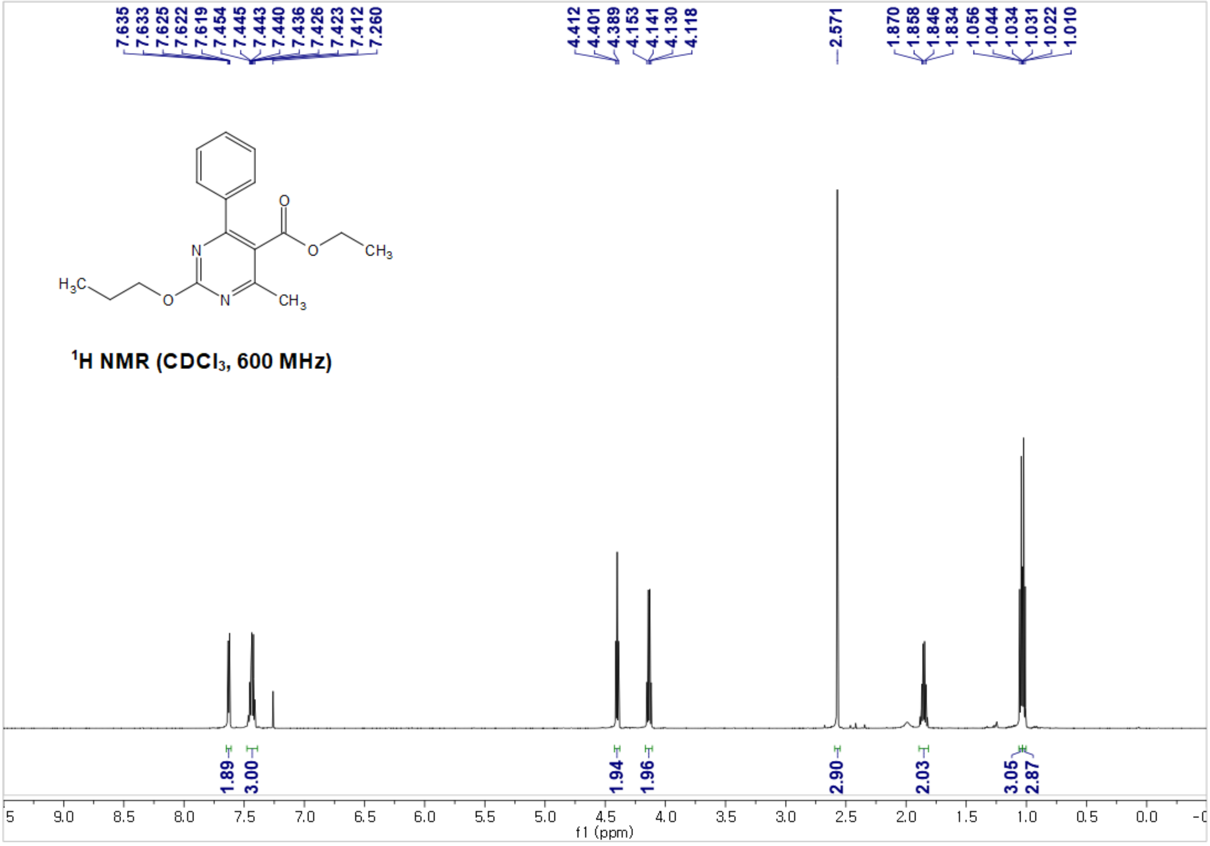

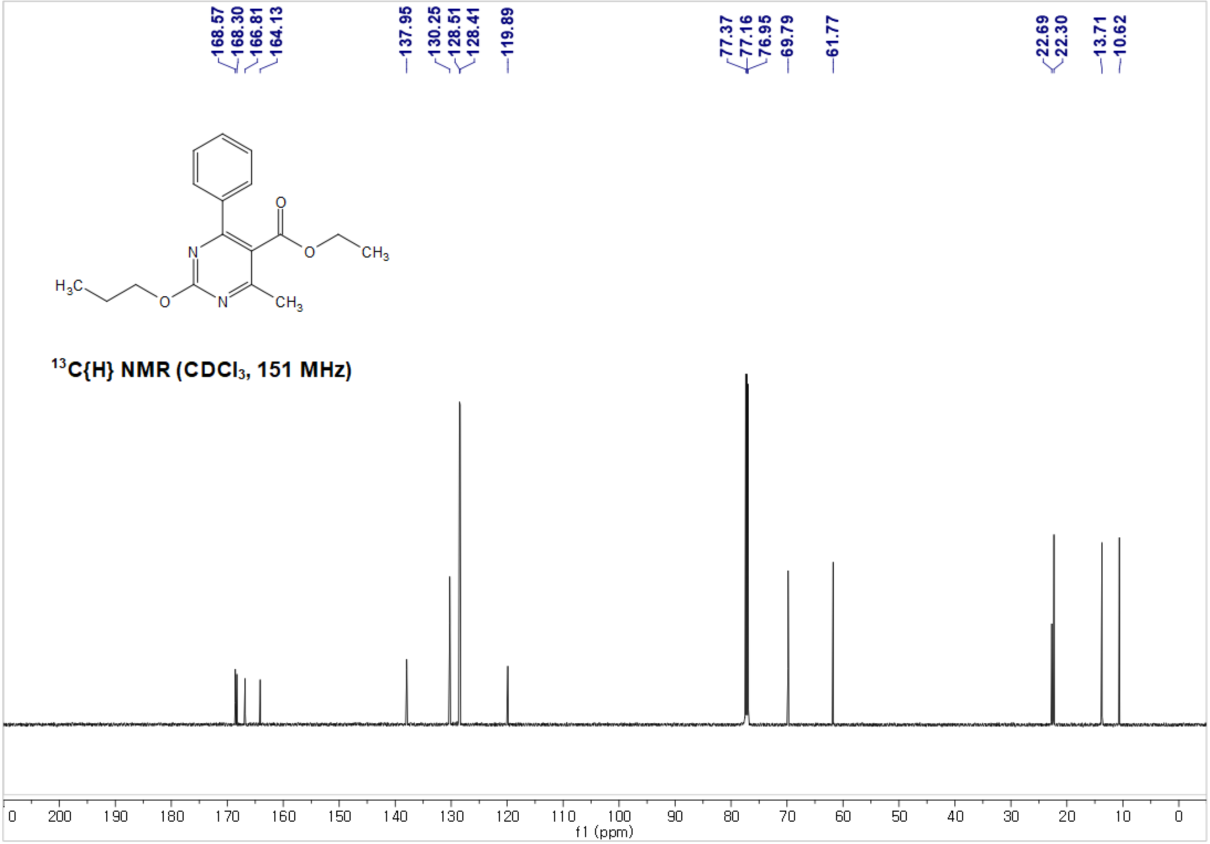

Ethyl 2-butoxy-4-methyl-6-phenylpyrimidine-5-carboxylate (**4d**)

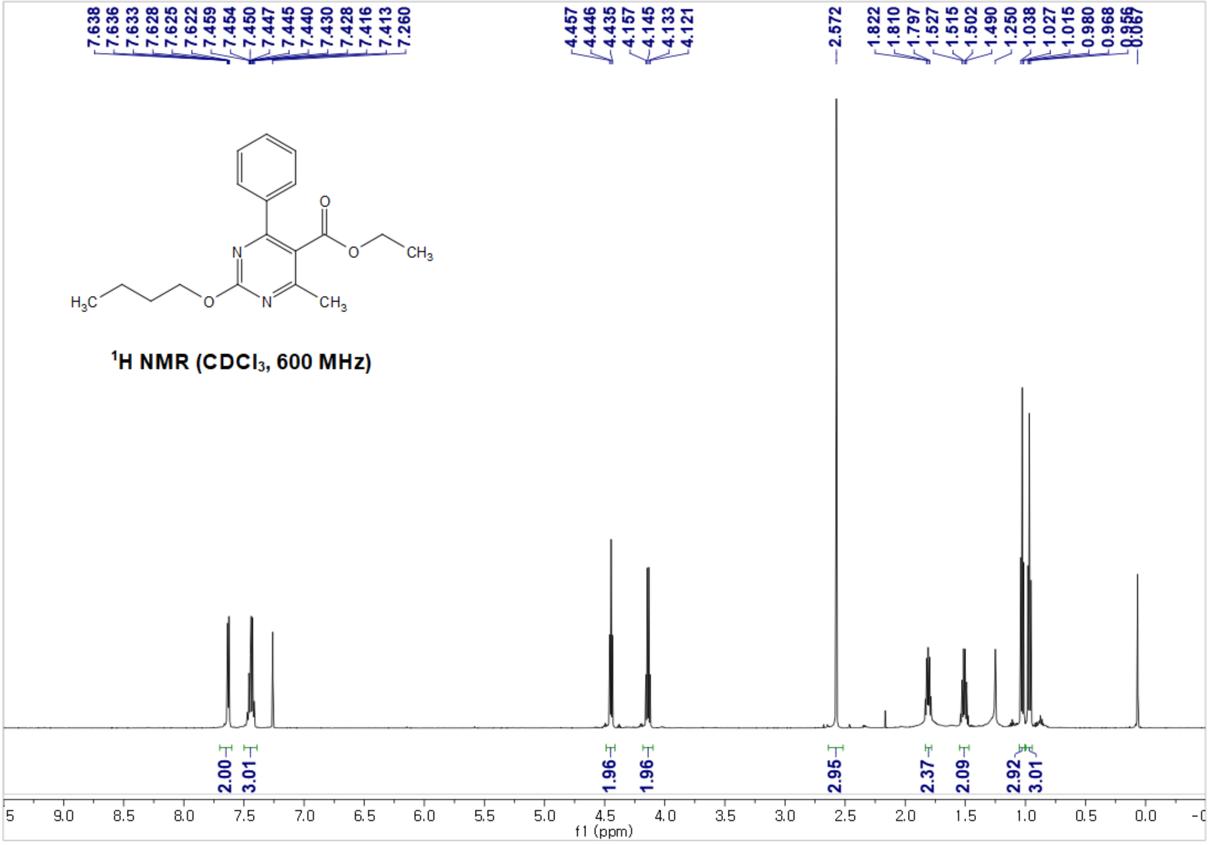

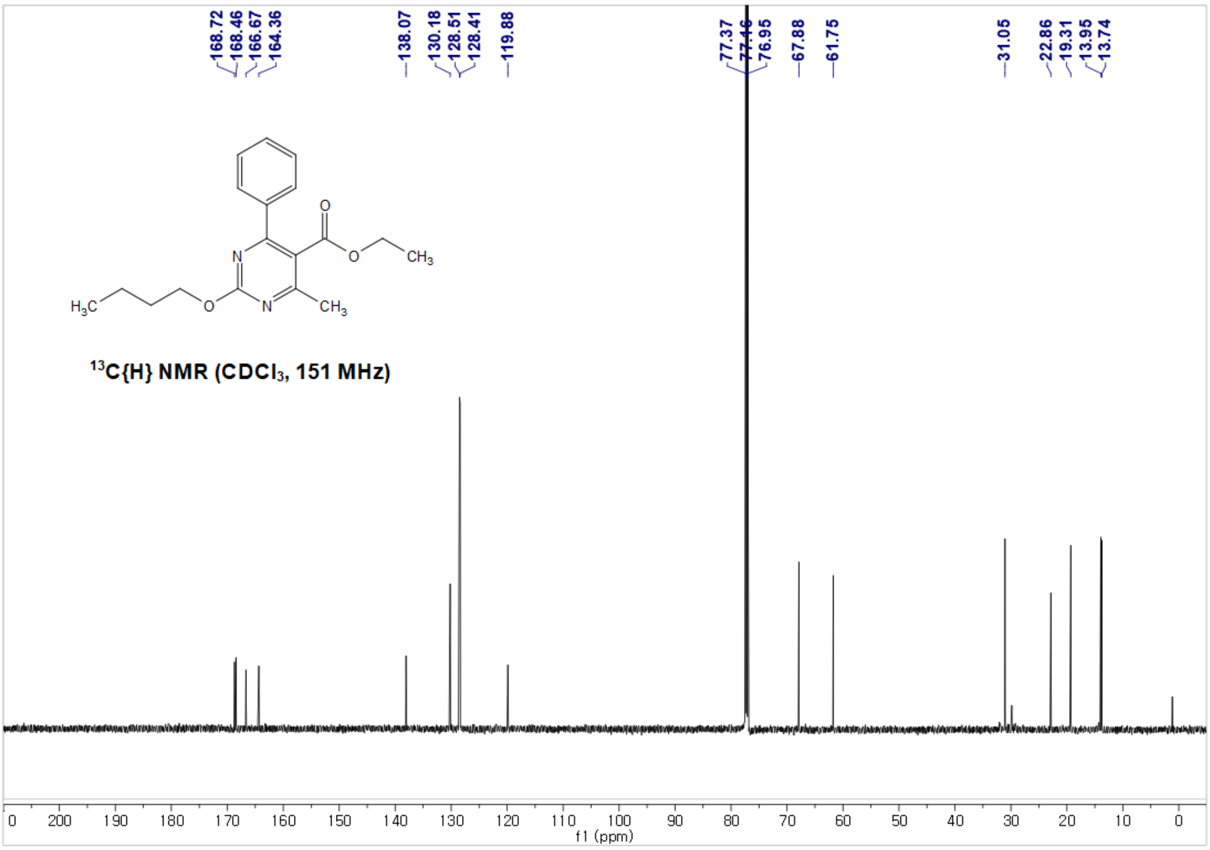

Ethyl 2-(benzyloxy)-4-methyl-6-phenylpyrimidine-5-carboxylate (**4e**)

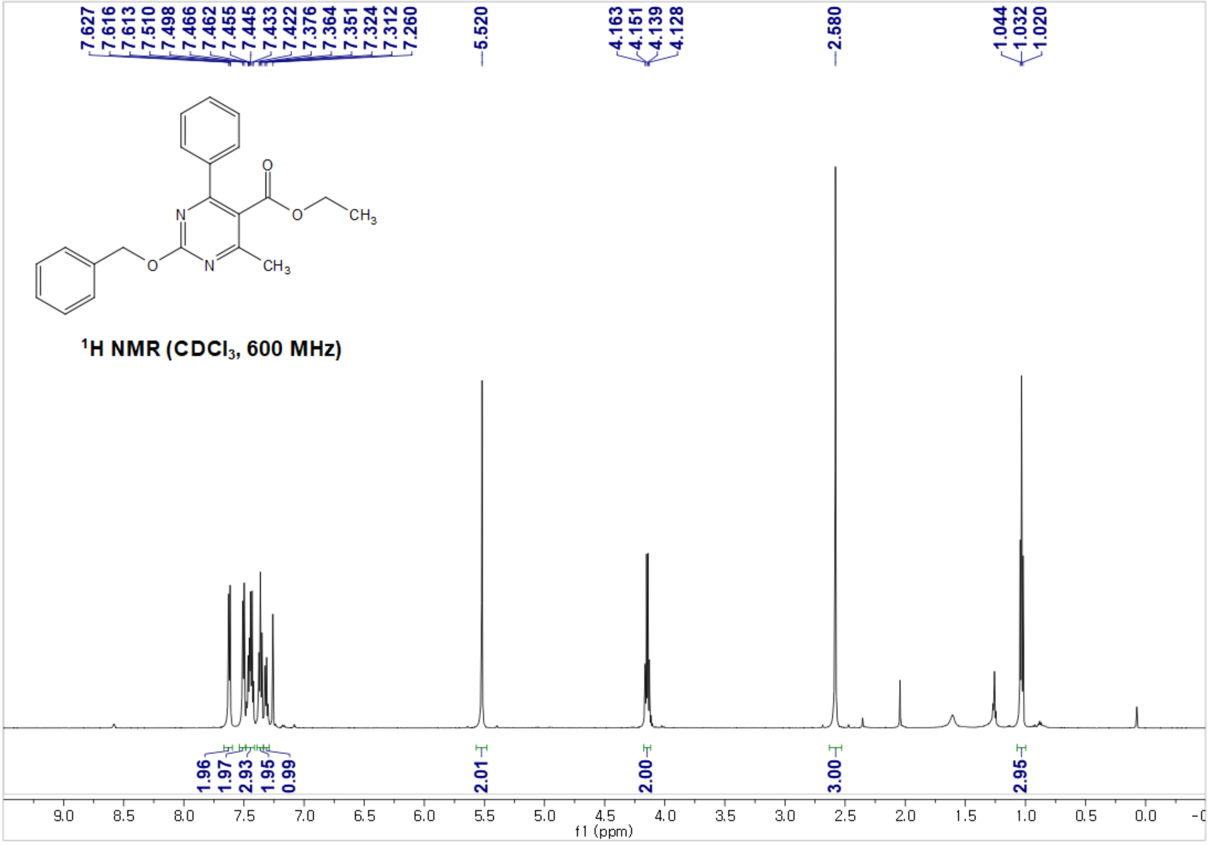

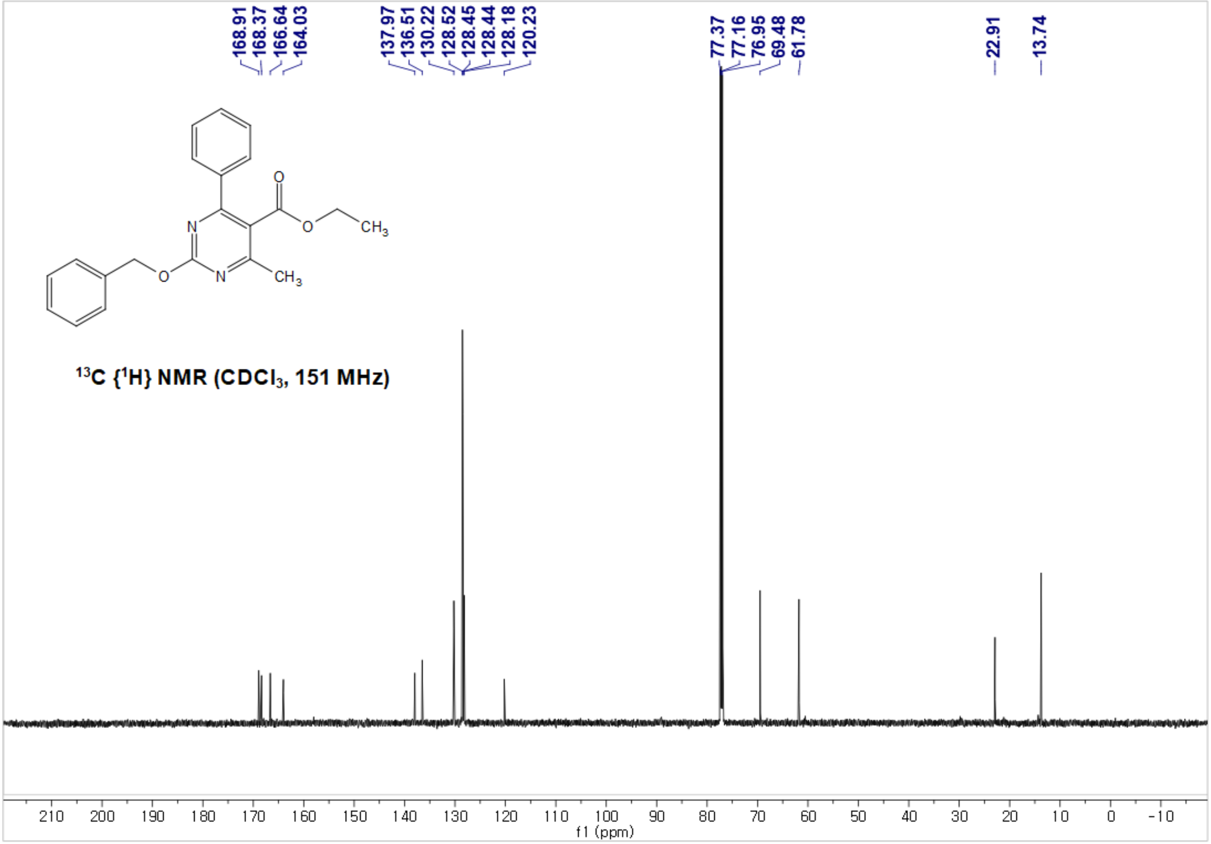

Ethyl 4-methyl-2-phenethoxy-6-phenylpyrimidine-5-carboxylate (**4f**)

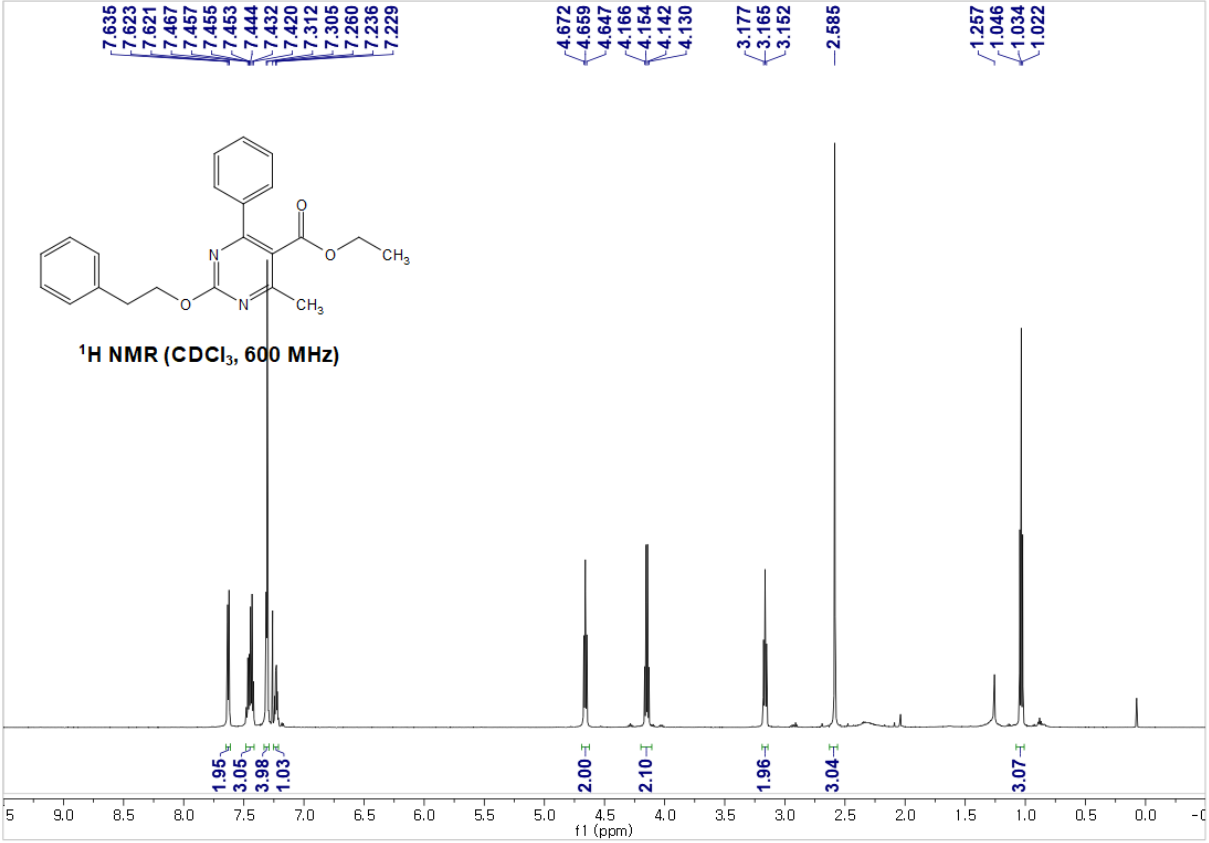

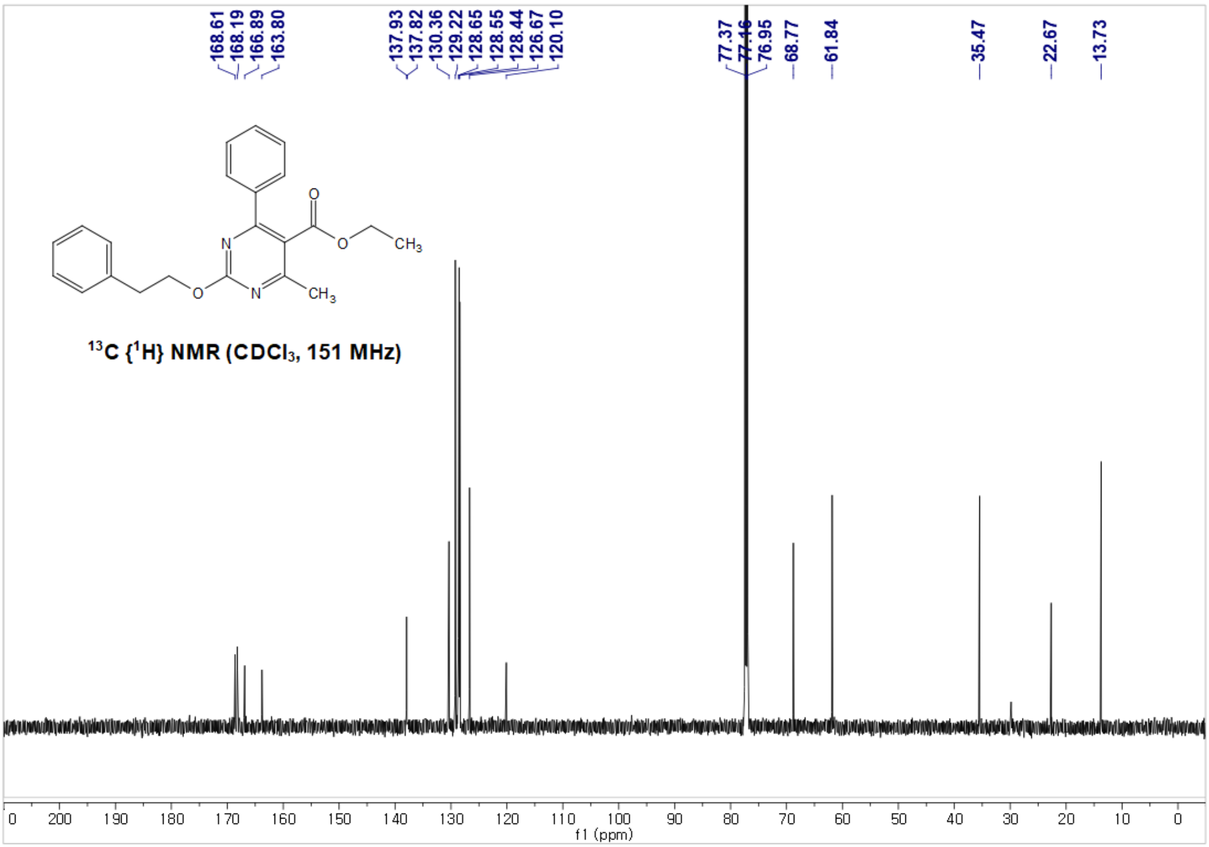

Ethyl 2-isopropoxy-4-methyl-6-phenylpyrimidine-5-carboxylate (**4g**)

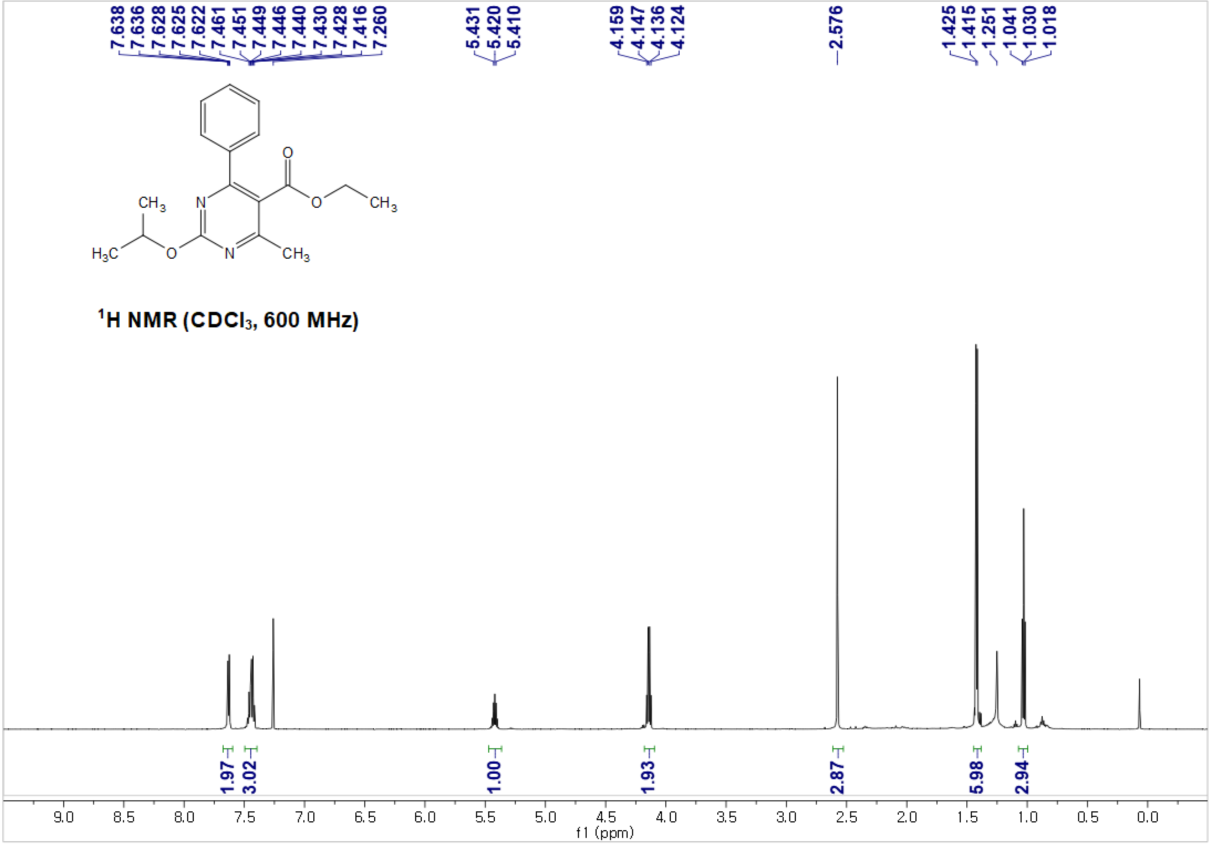

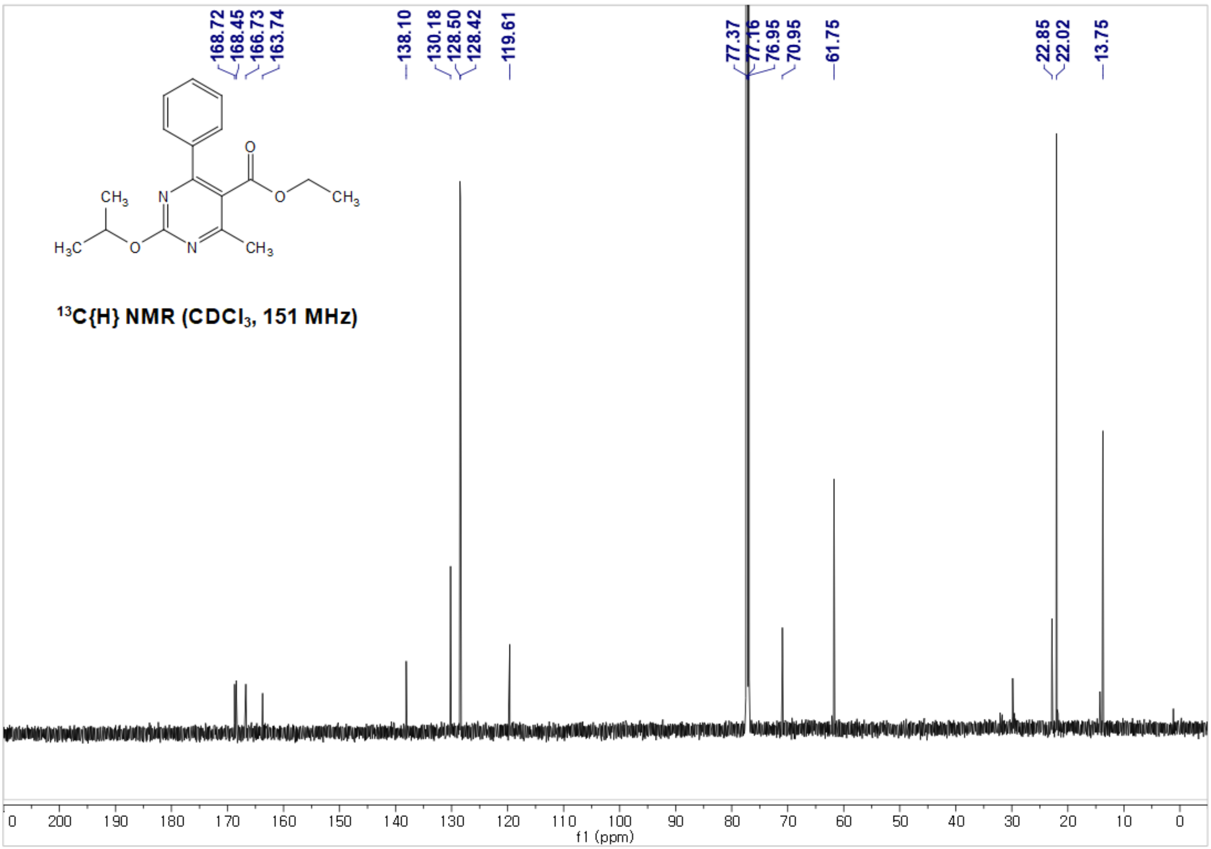

Ethyl 4-methyl-2-phenoxypyrimidine-5-carboxylate (**6**)

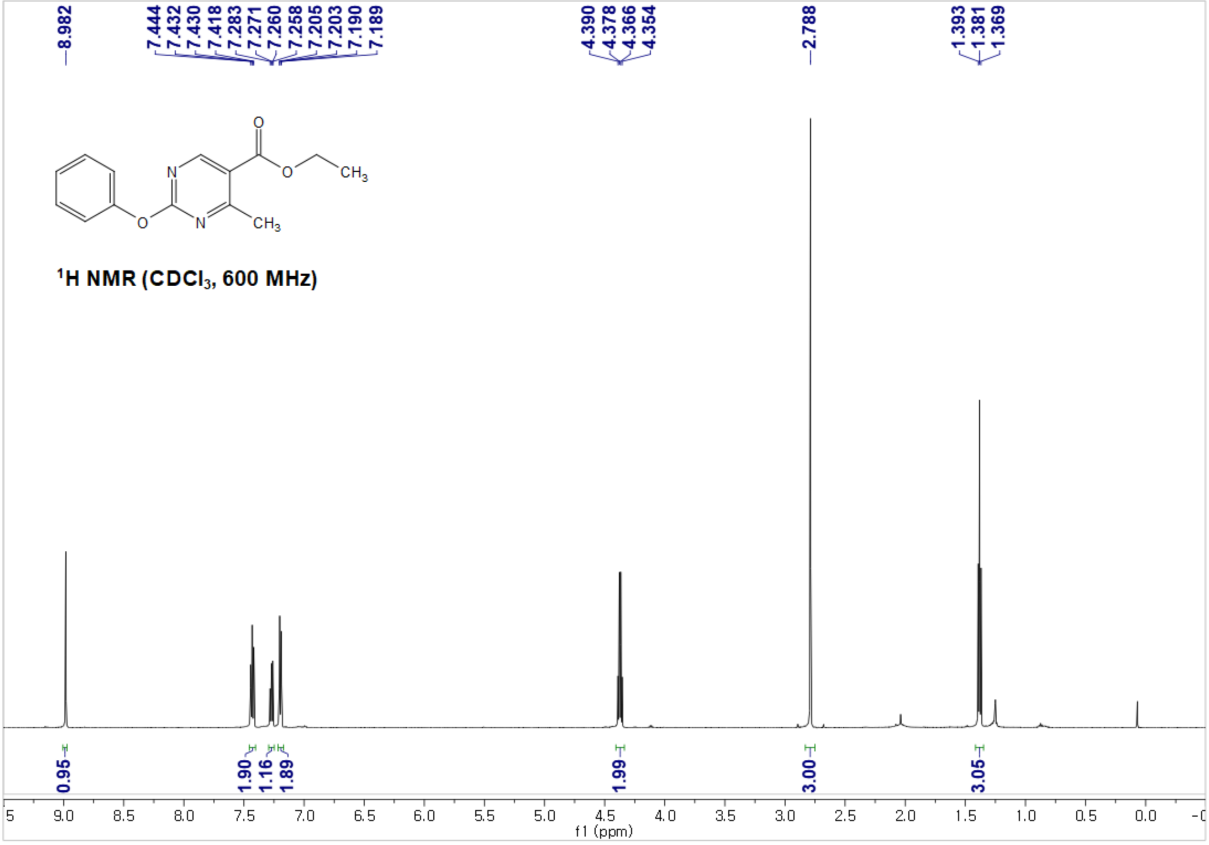

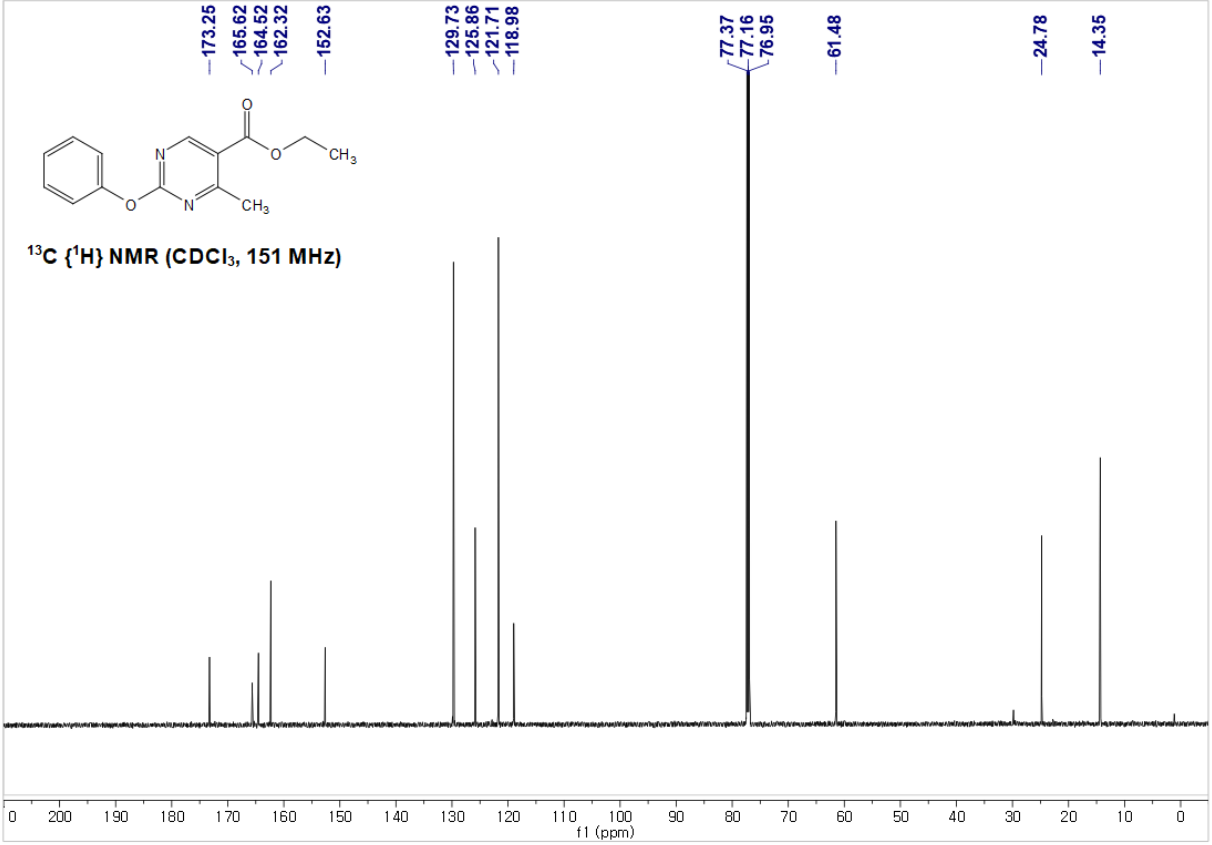

2-Phenoxypyrimidine (**8**)

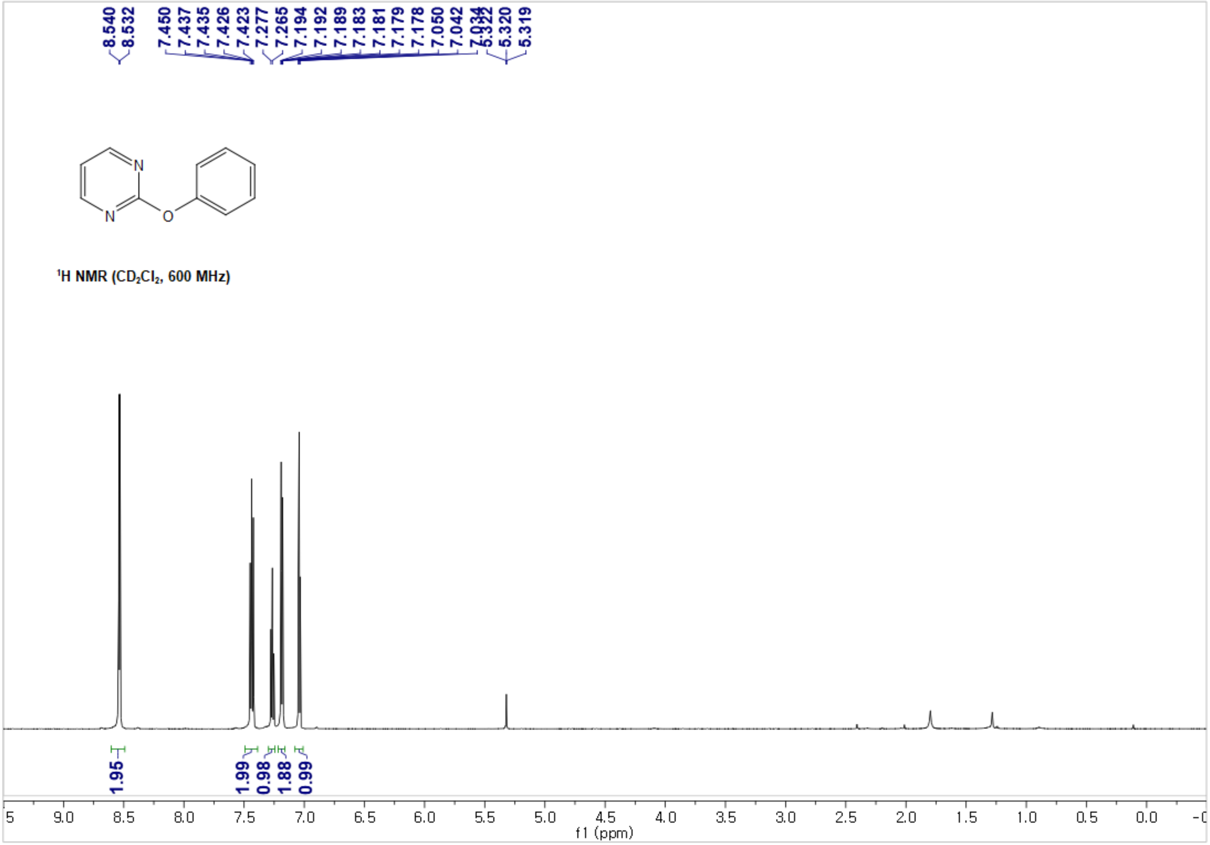

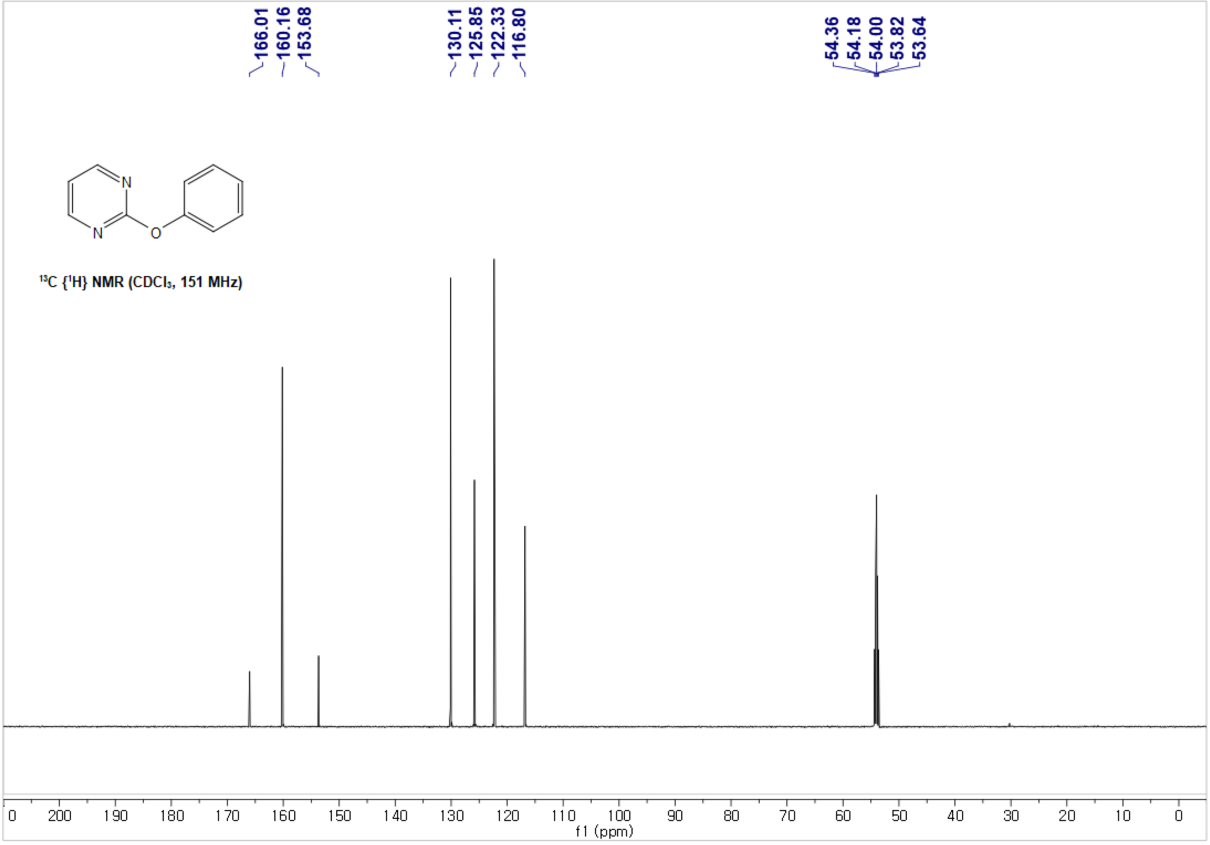

Ethyl 4-methyl-2-(methylthio)-6-phenylpyrimidine-5-carboxylate (**10**)

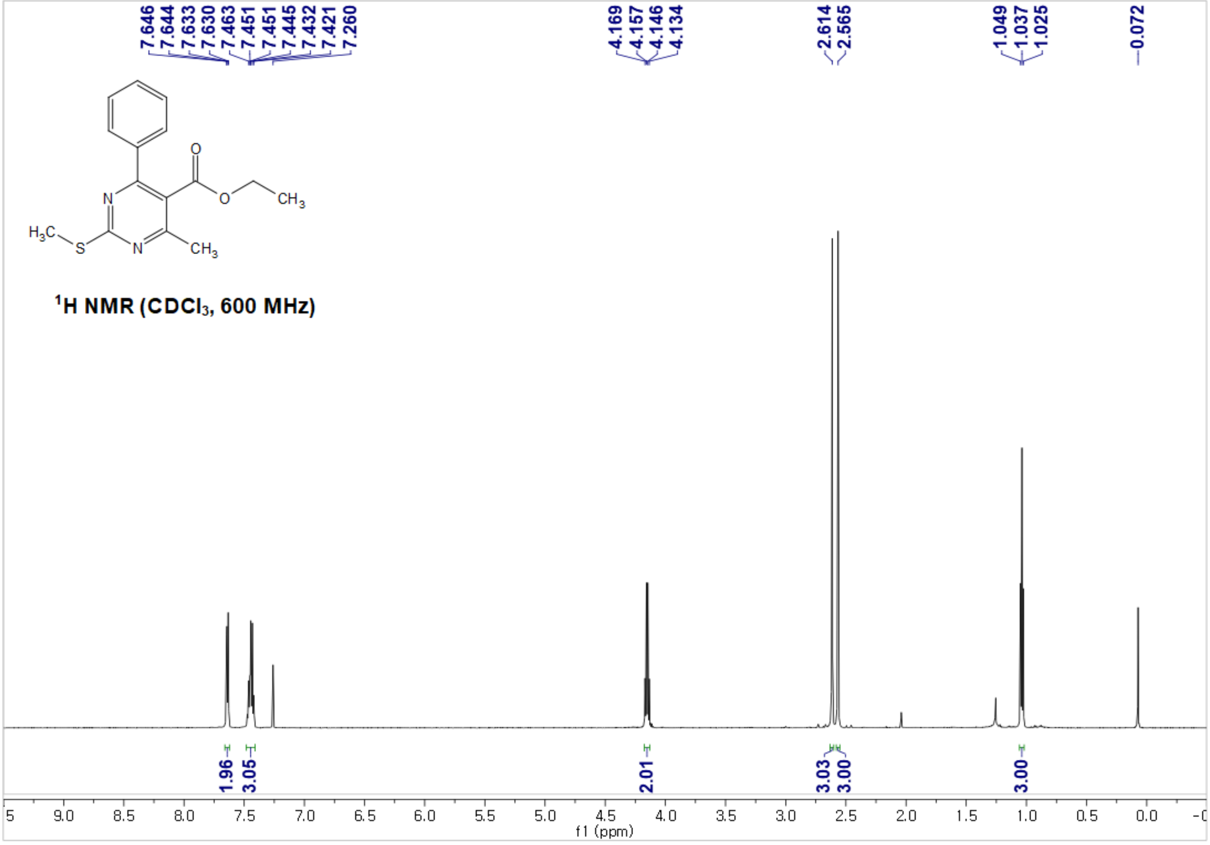

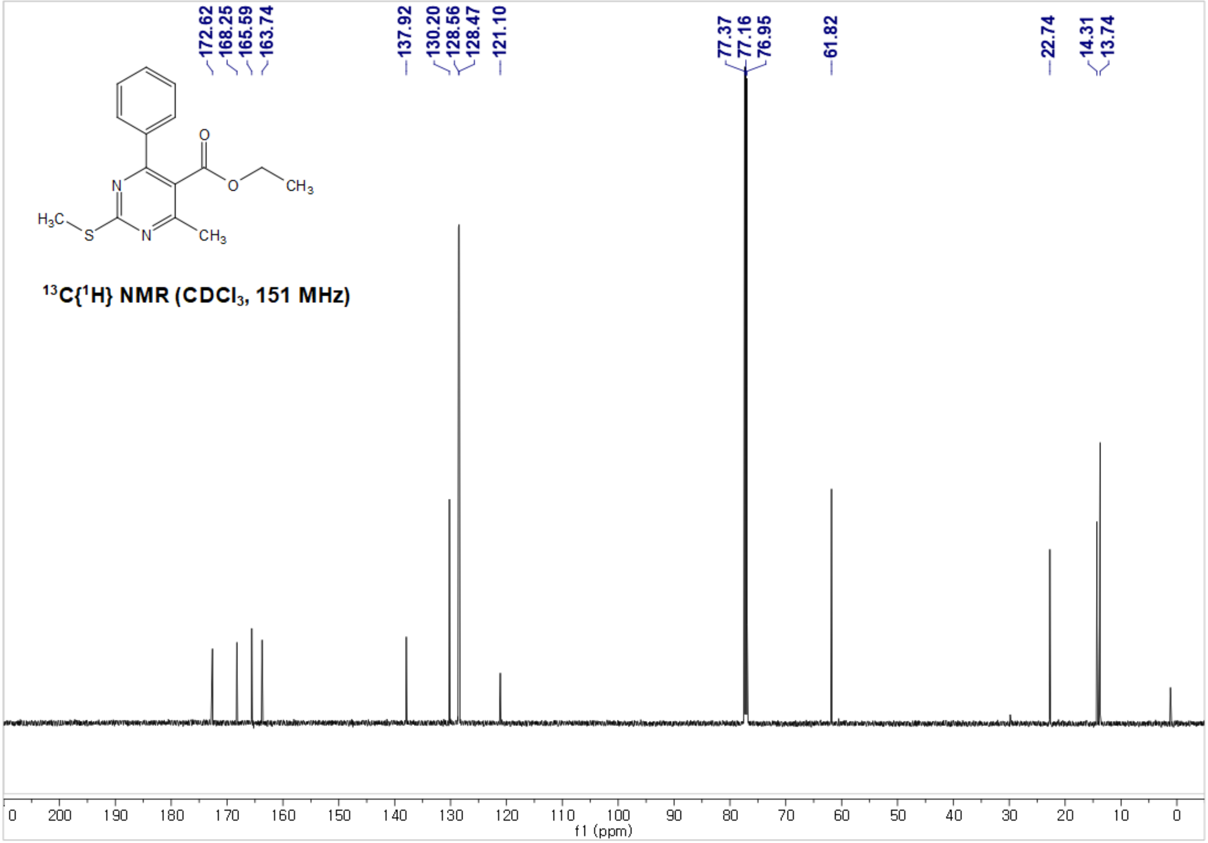

7. ^{19}F NMR spectrum of 3r

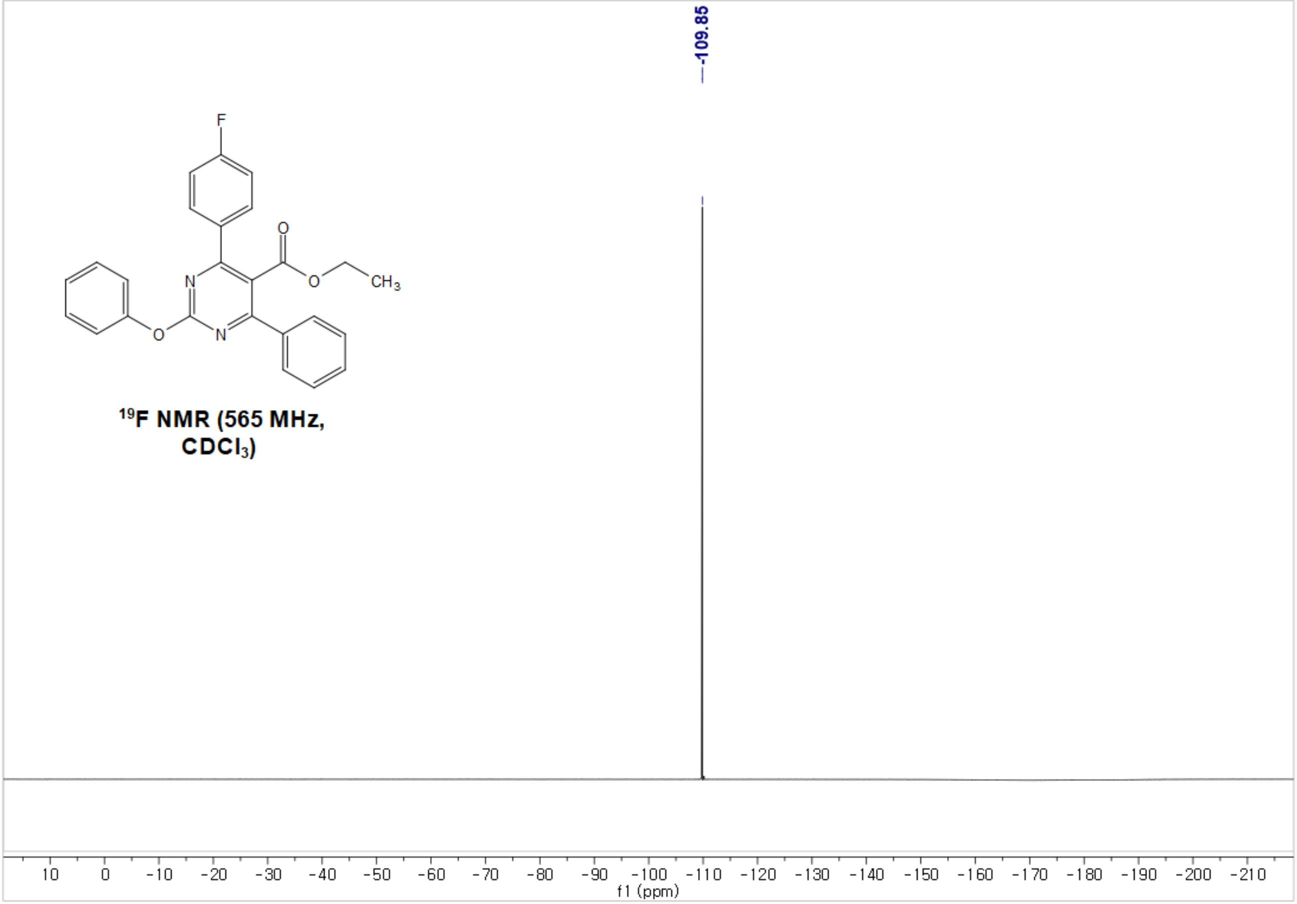

Supplement: RA-011-D1RA07713A-s001 [file RA-011-D1RA07713A-s001.pdf]
